# Calcium‐Catalyzed Arene C−H Bond Activation by Low‐Valent Al^I^

**DOI:** 10.1002/anie.201908978

**Published:** 2019-09-13

**Authors:** Steffen Brand, Holger Elsen, Jens Langer, Samuel Grams, Sjoerd Harder

**Affiliations:** ^1^ Inorganic and Organometallic Chemistry Universität Erlangen-Nürnberg Egerlandstrasse 1 91058 Erlangen Germany

**Keywords:** Aluminium, Calcium, C−H bond activation, Oxidative addition

## Abstract

The low‐valent ß‐diketiminate complex (^DIPP^BDI)Al is stable in benzene but addition of catalytic quantities of [(^DIPP^BDI)CaH]_2_ at 20 °C led to (^DIPP^BDI)Al(Ph)H (^DIPP^BDI=CH[C(CH_3_)N‐DIPP]_2_, DIPP=2,6‐diisopropylphenyl). Similar Ca‐catalyzed C−H bond activation is demonstrated for toluene or *p*‐xylene. For toluene a remarkable selectivity for *meta*‐functionalization has been observed. Reaction of (^DIPP^BDI)Al(*m*‐tolyl)H with I_2_ gave *m*‐tolyl iodide, H_2_ and (^DIPP^BDI)AlI_2_ which was recycled to (^DIPP^BDI)Al. Attempts to catalyze this reaction with Mg or Zn hydride catalysts failed. Instead, the highly stable complexes (^DIPP^BDI)Al(H)M(^DIPP^BDI) (M=Mg, Zn) were formed. DFT calculations on the Ca hydride catalyzed arene alumination suggest that a similar but more loosely bound complex is formed: (^DIPP^BDI)Al(H)Ca(^DIPP^BDI). This is in equilibrium with the hydride bridged complex (^DIPP^BDI)Al(*μ*‐H)Ca(^DIPP^BDI) which shows strongly increased electron density at Al. The combination of Ca‐arene bonding and a highly nucleophilic Al center are key to facile C−H bond activation.

## Graphical abstract

**The Al‐Ca team**: The oxidative addition of a low‐valent Al^I^ species to benzene at room temperature is catalyzed by a Ca hydride complex. The aromatic substrate is activated by π‐bonding to Ca, while hydride interaction with the empty Al p‐orbital boosts the nucleophilicity of the sp^2^ lone‐pair.

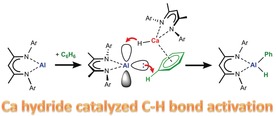

## Introduction

Efficient and selective C−H bond activation is one of the longstanding Holy Grails in chemistry.^1^ Being the subject of numerous reviews,^2^ it is a crucial technology towards the effective use of low‐cost feedstocks like simple alkanes or aromatics. Direct manipulation of the C−H bond enables synthetic pathways that avoid unnecessary functionalization steps, making it an attractive step‐economical goal in green chemistry.^3^ However, there are several major challenges, among which is the low reactivity of the strong, hardly polarized C−H bond. This explains why often harsh conditions are needed. In addition, the many C−H bonds present in organic molecules pose strict requirements on control over selectivity.

**Scheme 1:**
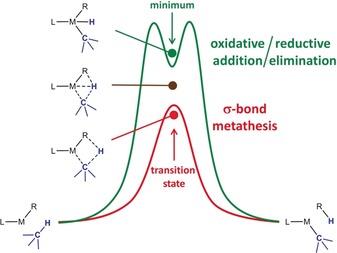
Two extreme pathways for C−H bond activation. Green: oxidative addition/reductive elimination (OA/RE). Red: σ‐bond metathesis (SBM), that is, deprotonation or metallation.

There are several different classifications of C−H bond cleavage reactions but the major pathways (Scheme 1) are divided between σ‐bond metathesis (SBM) and oxidative addition/reductive elimination (OA/RE).^4^ Transition between these extremes is smooth and depends on the strength of the metal⋅⋅⋅H interaction in the transition state or intermediate. For the SBM pathway as one extreme, it is non‐existent, while it is dominant for the OA/RE pathway as the other extreme.

**Scheme 2:**
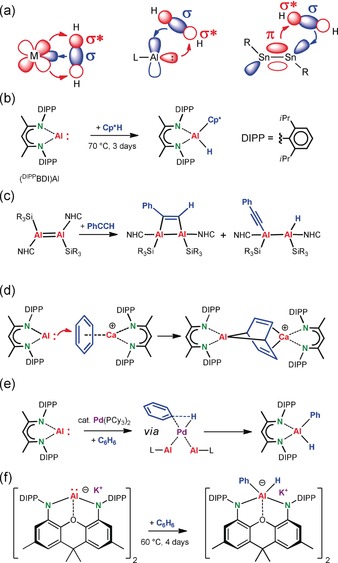
(a) Transition metal behavior of main group metals exemplary shown for H−H bond activation (from left to right: transition metal, mononuclear Al^I^ species or RSnSnR). (b,c) Examples for C−H bond activation at Al^I^ centers. (d) Activation and dearomatization of benzene by a cationic Ca complex and (^DIPP^BDI)Al. (e) Pd catalyzed benzene alumination. (f) Oxidative addition of an aluminyl anion to the C−H bond in benzene.

First examples of C−H bond activation dealt with aromatic substrates using precious platinum group metals like Ru or Pd.^5^ These pioneering studies have been fundamental to the development of Ir‐ or Rh‐mediated catalytic routes for borylation of arenes.^6^ Although C−H activation of unactivated arenes has gone a long way, examples of selective C−H bond activation without using directing groups are still limited and often need higher catalyst loadings.^7^ Apart from that, there is an increasing interest to replace the precious metals that are central to C−H bond activation for more abundant, less toxic, 3*d*‐metals.^8^ In this respect, main group metals would be highly advantageous. Directed or non‐directed, arene deprotonation by any strong *s*‐block metal base could be considered C−H bond activation through a SBM pathway (Scheme 1). Selectivity control in such metallation chemistry developed to a discipline of its own.^9^ It is, however, disputable whether the well‐established deprotonation reaction should be labeled C−H activation.^10^ Bond activation by oxidative addition, which is the general reaction found for most transition metals, is less common in main group chemistry. However, during the last decade it became clear that low‐valent main group metal complexes, despite their lack of *d*‐orbitals, can display transition metal‐like behavior in bond activation (Scheme 2 a).^11^ This is especially true for Al^I^ complexes, which show a very rich chemistry for activation of X−H bonds (X=H, B, Al, C, Si, Ge, Sn, N, P, As, O, S).^12^

Examples for C−H bond activation, however, are limited: Cp*H reacts under forced conditions (70 °C, 3 days) with (^DIPP^BDI)Al,12b a ß‐diketiminate complex with a rather high singlet‐triplet gap (30–40 kcal mol^−1^)^13^ introduced by Roesky and co‐workers (Scheme 2 b, ^[DIPP^BDI=CH[C(CH_3_)N‐DIPP]_2_, DIPP=2,6‐diisopropylphenyl).^14^ Similar, Schnöckel's Cp*Al^15^ has been shown to undergo reversible OA/RE with the activated diene C−H bond in Cp*H giving Cp*_2_AlH.^16^ Similar to this reactivity, Crimmin described cleavage of the activated allylic C−H bond in propene by (^DIPP^BDI)Al in benzene (80 °C, 14 h).^17^ Reaction of a dialumene with PhC≡CH also resulted in C−H activation (Scheme 2 c), apart from [2+2]‐cycloaddition.^18^ While similar dialuminene complexes react by [4+2]‐cycloaddition with benzene,^19^ calculations support the experimental verification that mononuclear (^DIPP^BDI)Al does not parallel such reactivity.^20^ Only after Lewis‐acid activation of benzene by a cationic Ca complex dearomatization is observed (Scheme 2 d).^21^ Despite this reactivity, C−H bond alumination of unactivated arenes is highly challenging. The Crimmin group recently reported the Pd‐catalyzed oxidative addition of (^DIPP^BDI)Al to C−H bonds in benzene, toluene, and xylenes and proposed a mechanism in which the C−H bond is activated at the Pd center (Scheme 2 e).^22^, ^23^ Hitherto, only a highly unusual nucleophilic Al anion was shown to activate the C−H bond in benzene (Scheme 2 f)^24^ but higher temperatures and long reaction times are needed. Most recently, it was shown that this reactivity can be topped by addition of a K^+^ selective cryptand and even cleavage of the C−C bond in benzene was achieved.^25^

## Results and Discussion

**Scheme 3:**
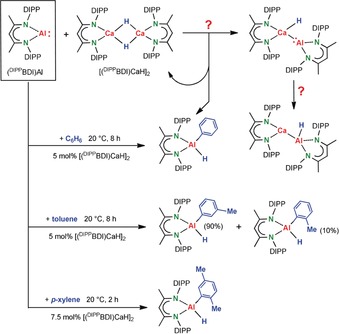
Attempted creation of a Ca−Al bond by stoichiometric reaction of (^DIPP^BDI)Al with [(^DIPP^BDI)CaH]_2_. Instead, calcium hydride catalyzed C−H bond activation of unactivated arenes by oxidative addition of (^DIPP^BDI)Al is observed.

Herein we report a serendipitous Ca hydride catalyzed C−H bond activation of several unactivated arenes by oxidative addition of (^DIPP^BDI)Al at room temperature (Scheme 3). As a follow‐up of our studies on the potential synthesis of heterobimetallic Al−Ca complexes (Scheme 2 d),^21^ we reacted (^DIPP^BDI)Al in C_6_D_6_ with the Ca hydride dimer [(^DIPP^BDI)CaH]_2_ in a 2/1 ratio. The latter hydride complex introduced by Hill and co‐workers^26^ is a Lewis base‐free form of the well‐established Ca hydride complex [(^DIPP^BDI)CaH⋅THF]_2_.^27^ Being more Lewis‐acidic, Al−Ca coordination could be envisioned (Scheme 3). Similar to earlier observed reactivity of (^DIPP^BDI)Al with metal‐hydride complexes,^12^ this may be followed by oxidative insertion of Al in the Ca−H bond. However, the reaction followed a completely different course.

While the Ca hydride complex was not converted, oxidative addition of (^DIPP^BDI)Al to the unactivated C−H bond of benzene was observed (Scheme 3). This reaction is smooth and, at room temperature, full conversion to (^DIPP^BDI)Al(Ph)H was reached within one hour. The latter Al^III^ complex was fully characterized and its crystal structure (Figure S21 in the Supporting Information) equals that reported by the Crimmin group.^22^ Since it is well‐known that (^DIPP^BDI)Al does not react with benzene^20^ and the Ca hydride complex remained fully intact, it seems reasonable that the latter is a catalyst for the observed oxidative addition. Indeed, using a catalyst loading of 5 mol % [(^DIPP^BDI)CaH]_2_ at room temperature led to full conversion within 8 hours (Table 1, entry 1) or within 3 hours at 60 °C (entry 2). Doubling the reaction time, the catalyst loading could be halved to 2.5 mol % (entry 3). In all cases only mono‐alumination was observed.

**Table 1:** Catalytic alumination of the C−H bond in unactivated arenes with (^DIPP^BDI)Al.^[a]^

Entry	Catalyst	mol %	Substrate	*T* [°C]	Time [h]	Conv. [%]
1	[(^DIPP^BDI)CaH]_2_	5	benzene	20	8	>99
2	[(^DIPP^BDI)CaH]_2_	5	benzene	60	3	>99
3	[(^DIPP^BDI)CaH]_2_	2.5	benzene	60	6	90
4	[(^DIPP^BDI)CaH]_2_	5	toluene	20	8	>99^[b]^
5	[(^DIPP^BDI)CaH]_2_	5	toluene	60	3	>99^[c]^
6	[(^DIPP^BDI)CaH]_2_	5	*p‐*xylene	20	8	73
7	[(^DIPP^BDI)CaH]_2_	7.5	*p‐*xylene	20	2	>99
8	[(^DIPP^BDI)CaH]_2_	5	mesitylene	60	8	0
9	(^DIPP^BDI)CaN(SiMe_3_)_2_	10	benzene	60	24	0
10	[(^DIPeP^BDI)SrH]_2_	5	benzene	20	168	>99

[a] [(^DIPP^BDI)Al]=0.04 m in C_6_D_6_. Reaction times for >99 % conversion (determined by ^1^H NMR monitoring in intervals of 30 minutes). [b] *o*/*m*/*p=*10/90/0. [c] *o/m*/*p=*17/83/0.

The smooth conversion of an unactivated aromatic substrate like benzene, poses the question whether electron‐rich methyl‐substituted arenes would be reactive towards Ca hydride catalyzed C−H alumination. Using 5 mol % catalyst loading, toluene could be converted equally efficient (entries 4–5), giving high selectivity for C−H bond activation in the *meta*‐position especially at room temperature. The raw product mixture did not show any conversion in the *para*‐position but a small quantity of *ortho*‐alumination is observed (*meta*/*ortho*=90/10). The crystal structure of (^DIPP^BDI)Al(*m*‐tolyl)H is shown in Figure S22. The high selectivity for C−H alumination in the *meta*‐position is unusual. Functionalization of mono‐substituted arenes is dominated by sterics and generally takes place in *meta*‐ and *para*‐positions roughly in a 2:1 ratio with at most trace amounts of *ortho*‐products.^28^ Using directing groups can give very high selectivity for *ortho*‐functionalization by the complex‐induced‐proximity‐effect (CIPE).^29^ Although in some cases the selectivity can be steered towards the *para*‐position by controlling electronics^30^ and/or sterics,^31^ selective *meta*‐functionalization remains very challenging^32^ and often needs catalysts with sophisticated directing ligands32d or templates.32e The high selectivity for *meta*‐alumination of toluene here observed also differs strongly from Pd catalyzed toluene alumination (*ortho*/*meta*/*para*=42/46/12).^22^ In the latter case, the unusually high percentage of *ortho*‐tolyl product has been explained by a weak attractive C−H⋅⋅⋅π interaction between the Me group of toluene and the aromatic ring of (^DIPP^BDI)Al.

*para*‐Xylene, an aromatic substrate with two Me‐substituents, was also fully converted albeit slightly slower (entry 6–7). It gave exclusively the fully characterized mono‐functionalized product (^DIPP^BDI)Al(2,5‐dimethylphenyl)H (see Figure S23 for crystal structure). Considering the *meta*‐directing influence of the Me group, strong activation of the *meta*‐CH bond in *meta*‐xylene was expected. However, *meta*‐ as well as *ortho*‐xylene did not show Ca hydride catalyzed C−H bond activation. Similar to the Pd catalyzed alumination as observed previously,^22^ the trisubstituted substrate mesitylene could not be converted (entry 8).

**Scheme 4:**
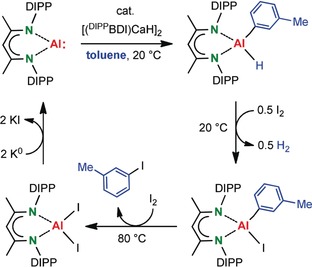
Cycle for the conversion of toluene and I_2_ to *m*‐tolyl iodide and H_2_ mediated by (^DIPP^BDI)Al and [(^DIPP^BDI)CaH]_2_.

Although not catalytically, we were able to run the reaction in a stoichiometric cycle by reaction of the alumination product with I_2_ (Scheme 4). The Al−H bond in (^DIPP^BDI)Al(*m*‐tolyl)H is considerably more reactive than the Al‐tolyl bond and reacts very fast at room temperature with 0.5 equivalent of I_2_ to give (^DIPP^BDI)Al(*m*‐tolyl)I and H_2_, apparent from vigorous gas development and ^1^H NMR monitoring (Figure S15). In a second, much slower step, (^DIPP^BDI)Al(*m*‐tolyl)I reacts with I_2_ to give *m*‐tolyl iodide and (^DIPP^BDI)AlI_2_ (Figure S16). To close the cycle, the latter Al iodide complex can be reduced with potassium to give (^DIPP^BDI)Al. Demonstration of this stoichiometric cycle is a first step towards the long‐term goal of making C−H bond activation a redox‐catalytic process that is also truly catalytic in the Al reagent.

Replacing catalyst [(^DIPP^BDI)CaH]_2_ with (^DIPP^BDI)CaN(SiMe_3_)_2_ (10 mol %) did not give any conversion, also not after heating to 60 °C (entry 9), implying the importance of a hydride functionality. In order to assess metal influences, we investigated the catalytic activity of the recently published Sr hydride complex [(^DIPeP^BDI)SrH]_2_.^33^ ^DIPeP^BDI is a bulkier version of the ^DIPP^BDI ligand in which *i*Pr groups have been replaced by isopentyl substituents. This highly reactive Sr hydride complex, which was previously shown to be active in H/D exchange with C_6_D_6_,^33^ gave full conversion in catalytic quantities but the reaction took one week for completion (entry 10). The considerable retardation of the catalytic reaction upon increasing the bulk of the BDI ligand underlines the importance of an open coordination site at the group 2 metal but could also be due to the lower Lewis‐acidity of Sr^2+^ vs. Ca^2+^.

**Figure 1:**
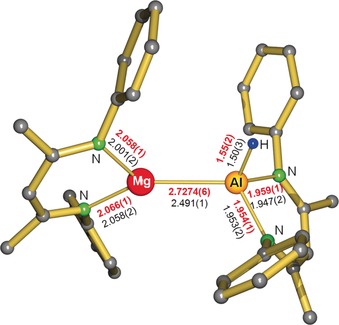
Crystal structure of (^DIPP^BDI)Al(H)Mg(^DIPP^BDI); H atoms, except the Al−H, and *i*Pr substituenst have been omitted for clarity. Selected bond distances (Å) for the Al−Mg complex are shown in red. Those for the isostructural Al−Zn complex (^DIPP^BDI)Al(H)Zn(^DIPP^BDI) are given in black.

Further influences of the catalyst metal have been studied by exchange of Ca for Mg or Zn, metals that show strong similiarities in their chemistry but are clearly more Lewis acidic than Ca. However, stoichiometric reaction of (^DIPP^BDI)Al with [(^DIPP^BDI)MgH]_2_ 34 or (^DIPP^BDI)ZnH^35^ in benzene followed a different course. Instead of benzene C−H alumination, insertion of Al in the metal hydride bond gave clean formation of (^DIPP^BDI)Al(H)M(^DIPP^BDI) complexes (M=Mg, Zn), which both have been fully characterized. While the crystal structure of the Zn complex showed major disorder (Figure S25), that of the Mg complex is well behaved (Figure 1). ^1^H and ^13^C NMR spectra for the Al−Zn complex show a unique signal for each C and H, 8 heptets and 16 doublets are observed for the *i*Pr‐substituents. The Al−Mg complex is more dynamic. Only after cooling of a [D_8_]toluene solution to −15 °C, a similar set of NMR signals was observed. These more pronounced dynamics are likely related to the weaker Al−Mg bond (vide infra).

The reactivity and crystal structures observed here show strong similarities with the insertion of (^DIPP^BDI)Al in the M‐R bond of (BDI)M‐R species (M‐R=Mg‐Me or Zn‐Et) reported earlier.^36^ The Mg−Al (2.7687(5) Å) and Zn−Al (2.488(1) Å) bond lengths in these complexes compare well to those depicted in Figure 1. The insertion of (^DIPP^BDI)Al in Mg−H or Zn−H bonds also shows parallels to the oxidative insertion of Cp*Al in the Zn−N bonds of Zn[N(SiMe_3_)_2_]_2_, which gave Zn[Al(Cp*)N(SiMe_3_)_2_]_2_ with Zn−Al bonds of 2.448(2) Å.^37^ Like previously reported Mg−Al and Zn−Al compounds, (^DIPP^BDI)Al(H)M(^DIPP^BDI) (M=Mg, Zn) complexes are also stable in refluxing benzene. Attempted crystallization of a similar Ca−Al complex from a solution of (^DIPP^BDI)Al and [(^DIPP^BDI)CaH]_2_ in methylcyclohexane as C−H bond activation inert solvent, only gave crystals of pure educts. This implies that a potential (^DIPP^BDI)Al(H)Ca(^DIPP^BDI) complex would be weakly bound. The ^1^H NMR spectrum of a 2/1 solution of (^DIPP^BDI)Al and [(^DIPP^BDI)CaH]_2_ in methylcyclohexane‐*d*_14_ as well only showed signals for separate educts. However, cooling to −50 °C led to the appearance of numerous additional signals that are reminiscent of (^DIPP^BDI)Al(H)M(^DIPP^BDI) (M=Mg, Zn): 8 new heptets for the *i*Pr‐CH group could be observed (Figure S17–18). Although structural identification of the product is hampered by the lack of single crystals, it is clear that (^DIPP^BDI)Al and [(^DIPP^BDI)CaH]_2_ are in equilibrium with at least one new species, which is present in small quantities of about 10 %. Indeed, DFT calculations (vide infra) show that the Ca−Al bond is much weaker than Mg−Al or Zn−Al bonds.

Further experimental investigation of the mechanism and the role of the Ca hydride catalyst in benzene alumination was found to be difficult. ^1^H NMR monitoring of the conversion of (^DIPP^BDI)Al into (^DIPP^BDI)Al(C_6_H_5_)H over time gave an untypical curve revealing an induction time indicative for an intermediate species (Figure S19–20). Indeed, during catalysis a set of signals for an unidentified species builds up and disappears towards the end of the reaction. Under all circumstances, we never detected the presence of H_2_, indicating that a deprotonation mechanism is unlikely. Deuterium labeling studies gave only limited insight. A solution of (^DIPP^BDI)Al and [(^DIPP^BDI)CaH]_2_ in C_6_D_6_ reacted to (^DIPP^BDI)Al(C_6_D_5_)D but H/D scrambling in the hydride position was found. Since a solution of [(^DIPP^BDI)CaH]_2_ and (^DIPP^BDI)Al(C_6_D_5_)D in C_6_H_6_ also led to rapid H/D exchange (Figure S12‐13), no further conclusions can be drawn. We also found that a solution of [(^DIPP^BDI)CaH]_2_ in C_6_D_6_ gave H/D exchange. This reactivity parallels the Et/D exchange found by Hill and co‐workers for a solution of [(^DIPP^BDI)CaEt]_2_ in C_6_D_6_ 26 but is much slower. While the origin of the hydride in the product remains questionable, it is clear that the C−H bond activation process is not reversible: a solution of (^DIPP^BDI)Al(C_6_D_5_)D in C_6_H_6_ in the presence of the Ca hydride catalyst gave at most hydride D/H scrambling but no C_6_D_5_/C_6_H_5_ exchange was observed.

**Scheme 5:**
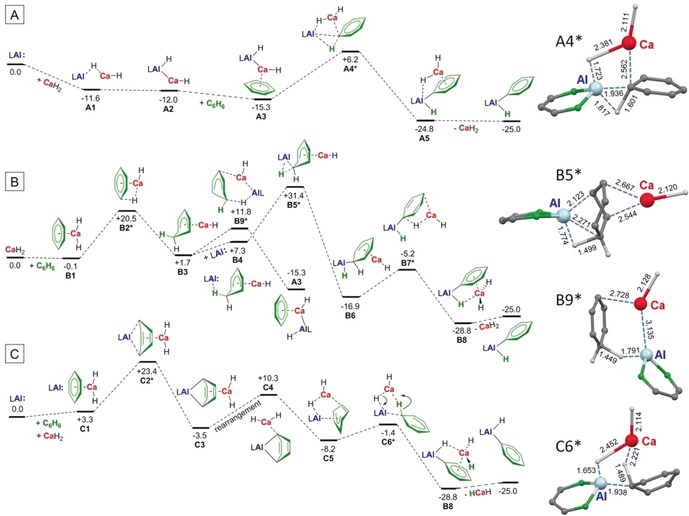
Three potential mechanisms calculated for the Ca hydride catalyzed alumination of benzene (ωB97XD/6‐311+G**(PCM=benzene)//ωB97XD/6‐31+G**, relative Δ*G* values at 298 K and 1 bar are given in kcal mol^−1^).

To provide a reasonable explanation for the herein reported facile C−H bond activation at room temperature, the reaction of (^DIPP^BDI)Al with benzene was investigated by DFT calculation (ωB97XD/6‐311+G**//ωB97XD/6‐31+G**, Δ*G* values in kcal mol^−1^ at 298 K and 1 bar are corrected for solvent effects in benzene using the PCM method). Since the reaction runs equally well in the dark and the singlet‐triplet gap in (^DIPP^BDI)Al is larger than 30 kcal mol^−1^,^13^ we did not consider radical mechanisms but evaluated three different closed shell processes in which, for simplicity and reduction of computation time, the Ca hydride catalyst was modeled by CaH_2_ (Scheme 5): **A** direct oxidative addition, **B** the Meisenheimer anion route and **C** the C_6_H_6_^2−^ route.

Pathway **A**: The direct oxidative addition of low‐valent (^DIPP^BDI)Al to the C−H bond in benzene yielding (^DIPP^BDI)Al(Ph)H is exergonic by −25.0 kcal mol^−1^. Without a catalyst, the activation energy for this process is 51.0 kcal mol^−1^ (Figure S27). However, the presence of CaH_2_ reduces this barrier significantly. First, a (^DIPP^BDI)Al(μ‐H)CaH complex (**A1**) is formed in which a hydride ligand binds to Al along its empty p‐orbital. An alternative minimum in which the hydride is fully transferred to Al under formation of an Al−Ca bond (**A2**) is 0.4 kcal mol^−1^ lower in energy. Complexation of **A1** with benzene also leads to hydride transfer and Al−Ca bond formation (**A3**). The latter Al−Ca bond is weak and not present in transition state A4*, in which Ca is fully bound to benzene. The low barrier for insertion (**A3**–A4*: 21.5 kcal mol^−1^) is in agreement with the room temperature reaction and originates from activation by Ca‐C_6_H_6_ π‐coordination and strong hydride‐to‐Al interaction. This hydride‐Al contact increases the nucleophilicity of the Al sp^2^ lone‐pair significantly and, similar to the Al anion in Scheme 2 f, C−H bond activation is facilitated.

Pathway **B**: The Meisenheimer anion route starts with the formation of a benzene‐CaH_2_ complex (**B1**), which is followed by an unusual nucleophilic aromatic attack (B2*). This reactivity is reminiscent to Et/D exchange in a solution of [(^DIPP^BDI)CaEt]_2_ in C_6_D_6_, recently reported by Hill and co‐workers,^26^ or to H/D exchange in a solution of [(^DIPeP^BDI)SrH]_2_ in C_6_D_6_ published by us.^33^ Assisted by Ca‐benzene complexation, the energy barrier for this reaction is only 20.6 kcal mol^−1^ and formation of Meisenheimer complex **B3** is slightly endergonic. Starting from **B3** there are two possibilities: (^DIPP^BDI)Al attacks the C_6_H_7_^−^ ion either from the Ca‐bound side or form the opposite direction. The latter leads to precomplexation (**B4**) and an unrealistically high transition state (B5*), after which conversion via **B6**–**B8** is essentially barrier‐free. Attack from the Ca‐bound side leads to facile hydride transfer from the C_6_H_7_^−^ ion to Al (B9*). Due to a stabilizing Al‐Ca interaction, the barrier for this transition state is considerably lower. However, the product of this pathway is the Al‐Ca complex **A3**. This means that pathway **B** represents an alternative, but more difficult route for formation of **A3,** which further follows pathway **A3**→A4*→**A5**.

Pathway **C**: The route that involves an anti‐aromatic C_6_H_6_^2−^ intermediate is reminiscent of our earlier work on Ca/Al assisted benzene reduction (Scheme 2 d).^21^ The uncatalyzed reaction of (^DIPP^BDI)Al with benzene to give the Al^III^ complex (^DIPP^BDI)Al(C_6_H_6_) is endergonic by +6.5 kcal mol^−1^ and an activation energy of 33.0 kcal mol^−1^ is required (Figure S28). In contrast, the highest barrier calculated for nucleophilic addition to a benzene‐CaH_2_ complex (**B1**) is only 23.4 kcal mol^−1^ (C2*) and formation of **C3** is exergonic. The further course of the reaction is a rearrangement of the weakly bound CaH_2_ to the bridgehead carbon (**C4**) which further rearranges to **C5**, a complex in which CaH_2_ bridges between Al and one of the C=C bonds. Starting from here, the concerted double H‐transfer process via C6* is essentially barrier‐free.

**Figure 2:**
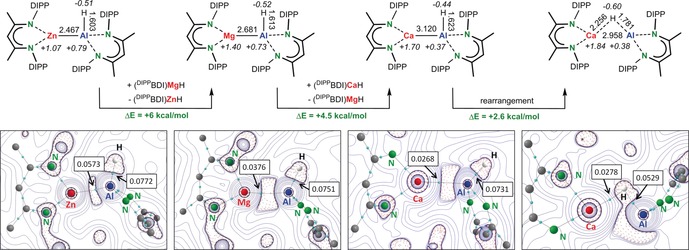
Top: Calculated structures of (^DIPP^BDI)Al(H)M(^DIPP^BDI) (M=Zn, Mg, Ca) at the ωB97XD/6‐311+G**//ωB97XD/6‐31+G** level; bond distances in Å and NPA charges shown in italic. The stability of the complexes decreases from M=Zn > Mg > Ca. Bottom: Contour plots of the Laplacian of the electron density (AIM); electron density in the bond‐critical‐point given in au.

**Scheme 6:**
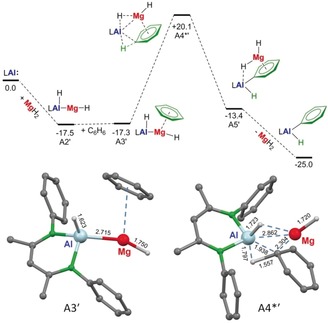
Pathway A recalculated for MgH_2_ instead of CaH_2_ as catalyst (ωB97XD/6‐311+G**(PCM=benzene)//ωB97XD/6‐31+G**, Δ*G* values at 298 K and 1 bar given in kcal mol^−1^) clearly showing a much higher activation energy: A3′→A4*′ +37.8 kcal mol^−1^.

Using D‐labeling, one should be able to discriminate between pathways **A**/**B** and **C**. However, facile hydride exchange between Ca and Al (vide supra) does not allow for any further experimental verification. Although the model system chosen for this computational study may be simple, it indicates that route **A** seems to be the most likely pathway. It is also in agreement with the experimentally observed induction time and is in line with the formation of a stable intermediate (**A1**–**A2**) as well as its non‐reversibility: conversion **A5**→A4* would require 31.0 kcal mol^−1^. Interestingly, recalculating pathway **A** with Mg instead of Ca gave a much higher activation barrier of +37.4 kcal mol^−1^ for the C−H bond braking step (Scheme 6). This explains the stability of (^DIPP^BDI)Al(H)Mg(^DIPP^BDI) in refluxing benzene. We therefore believe that the key to Ca‐catalyzed benzene alumination is the very weak Ca⋅⋅⋅Al interaction which precluded isolation of a mixed Ca/Al species. Although labile, we were able to compute the minimum for (^DIPP^BDI)Al(H)Ca(^DIPP^BDI) which shows an extraordinary long Ca⋅⋅⋅Al contact of 3.120 Å (Figure 2). It has to be note that this Ca⋅⋅⋅Al contact is of similar length as that in the model system (^DIPP^BDI)Al(H)CaH (**A2**) of 3.165 Å, confirming that CaH_2_, although small, is a reasonable substitute for (^DIPP^BDI)CaH in the computational study.

It is instructive to compare calculated M‐Al distances in the row of complexes (^DIPP^BDI)Al(H)M(^DIPP^BDI) with M=Zn 2.491(1) Å, Mg 2.681 Å and Ca 3.120 Å. While the calculated values for the Zn−Al and Mg−Al bonds compare reasonably well with experimental values, the increments from Zn to Mg (0.19 Å) and Mg to Ca (0.439 Å) are much larger than the differences in ionic radii (Zn^2+^ 0.74 Å, Mg^2+^ 0.72 Å, Ca^2+^ 1.00 Å).^38^ The long Ca⋅⋅⋅Al contact is likely due to its substantial electrostatic character. According to Allred‐Rochow electronegativities (Al 1.47, Zn 1.66, Mg 1.23 and Ca 1.04)^39^ bond polarities increase along the row Zn−Al < Mg−Al < Ca−Al. Bond weakening along the row Zn−Al > Mg−Al > Ca−Al is also clear from metal exchange energies given in Figure 2. The Zn−Al bond is more than 10 kcal mol^−1^ more stable than the Ca−Al bond. Atoms‐In‐Molecules (AIM) analyses of the complexes show increasing bond‐critical‐point electron densities, and therefore increasing bond strength and covalency, along the row Ca−Al < Mg−Al < Zn−Al. The lability of the Ca−Al bond is demonstrated by localization of a second minimum with a bridging hydride, (^DIPP^BDI)Al(*μ*‐H)Ca(^DIPP^BDI), which is only 2.6 kcal mol^−1^ higher in energy. Although the Ca−Al bond distance of 2.958 Å is considerably shorter than the 3.120 Å distance in the Ca−Al bound species, there is no bond‐critical‐point along the Ca⋅⋅⋅Al axis. The contour plot of the Laplacian of the electron density clearly shows that the electron pair is located at Al. Similar minima for Zn(*μ*‐H)Al or Mg(*μ*‐H)Al species could not be located.

The largely different nature of the Zn−Al, Mg−Al and Ca−Al bonds is also clear from NPA charges (Figure 2). The most remarkable difference is found for the NPA charges: the Al center in the Ca−Al complex is clearly less positive, the charge of +0.37 is unusually low and also considerably less than that on Al in (^DIPP^BDI)Al of +0.82. Therefore, the Ca−Al complex could be regarded as the ion‐pair [(^DIPP^BDI)Ca]^+^ [(^DIPP^BDI)Al(H)]^−^. This is supported by NPA charges of +0.769/−0.769 calculated for these ions. The aluminyl anion [(^DIPP^BDI)Al(H)]^−^ in this ion pair is reminiscent of the C−H bond activation of the anionic Al complex reported by the Aldrich and Goicoechea groups.^24^, ^25^ It is proposed that the [(^DIPP^BDI)Ca]^+^ cation and [(^DIPP^BDI)Al(H)]^−^ anion operate in concert. The combination of Ca⋅⋅⋅C_6_H_6_ complexation and strong nucleophilicity of the aluminyl anion are crucial to the herein presented facile C−H bond activation.

## Conclusion

Cleavage of the sp^2^ C−H bond in unactivated arenes (benzene, toluene, xylene) by the low valent Al^I^ complex (^DIPP^BDI)Al has been achieved at room temperature. In contrast to previously reported Pd‐catalyzed benzene alumination,^22^, ^23^ this challenging reaction can also be mediated by the early main group metal hydride complex [(^DIPP^BDI)CaH]_2_. This observation contributes to the growing awareness that d‐orbital participation is not an essential requirement for oxidative addition to unactivated C−H bonds.

For toluene, a remarkable selectivity for *meta*‐functionalization has been observed. Functionalization with elemental I_2_ gave conversion to *m*‐tolyl iodide. Although this reaction is not catalytic in Al, a protocol for recycling to the starting material (^DIPP^BDI)Al is presented. However, the challenge to use both, the Ca hydride and low‐valent Al^I^ complexes, in a true catalytic sense still remains a long‐term goal.

The mechanism for the Ca hydride catalyzed alumination of benzene has been investigated by experiment and theory. Conversion vs. time does not follow simple kinetics but suggests the formation of an intermediate. From D‐labeling studies, it is unclear whether the source of the hydride in the product is the arene or the catalyst. Since the product (^DIPP^BDI)Al(C_6_D_5_)D and the [(^DIPP^BDI)CaH]_2_ catalyst show D/H scrambling, no further conclusions could be drawn. Replacing Ca for Mg or Zn fully deactivated the catalyst and the mixed metal products (^DIPP^BDI)Al(H)M(^DIPP^BDI) (M=Mg, Zn) have been characterized by single crystal X‐ray diffraction. The latter are extremely stable and do not react with benzene, even under reflux conditions.

Three different pathways have been evaluated by DFT calculations. This study indicates that the first reaction step is the formation of a loosely bound Ca−Al complex, (^DIPP^BDI)Al(H)Ca(^DIPP^BDI), which is in equilibrium with the hydride bridged complex (^DIPP^BDI)Al(*μ*‐H)Ca(^DIPP^BDI). Bonding of the hydride along Al's empty p‐orbital axis leads to increased nucleophilicity of the Al center. This is supported by calculation of an unusually low positive charge on Al. Similar as for the first aluminyl anions,^24^, ^25^ the mixed metal Ca−Al species is activated for facile oxidative addition to a C−H bond in an arene. Since it is the combined action of a nucleophilic Al center and arene activation by π‐coordination to a Lewis acidic Ca center, parallels to frustrated Lewis pair chemistry may be drawn.^40^

The herein observed activation of Al^I^ centers by metal hydride interaction is unique. Replacing Pd with a Ca‐based catalyst for a challenging reaction like the C−H bond activation in unactivated arenes represents another jewel in the crown of the rapidly developing field of *s*‐block metal catalysis.^41^ The concept of activation of (^DIPP^BDI)Al by addition of a polar reagent can likely be extended much further and is currently comprehensively investigated.

## Conflict of interest

The authors declare no conflict of interest.

## Supporting information

As a service to our authors and readers, this journal provides supporting information supplied by the authors. Such materials are peer reviewed and may be re‐organized for online delivery, but are not copy‐edited or typeset. Technical support issues arising from supporting information (other than missing files) should be addressed to the authors.

Supplementary
