# Supplementary material for: Calcium‐Catalyzed Arene C−H Bond Activation by Low‐Valent AlI
Source: Angew Chem Int Ed Engl. 2019 Sep 13;58(43):15496–503. doi: 10.1002/anie.201908978 (PMC6856855; doi:10.1002/anie.201908978)
Supplement: Supplementary file 1 — Supplementary [file ANIE-58-15496-s001.pdf]

Angewandte Chemie
Eine Zeitschrift der Gesellschaft Deutscher Chemiker

Supporting Information

Calcium-Catalyzed Arene C–H Bond Activation by Low-Valent Al^{I}

*Steffen Brand, Holger Elsen, Jens Langer, Samuel Grams, and Sjoerd Harder**

anie_201908978_sm_miscellaneous_information.pdf

SUPPORTING INFORMATION

Contents

1. Supporting Experimental Data S1

1.1. General Experimental Procedures S1

1.2. Synthesis S1

1.3 NMR Spectra of Synthesized Compounds S6

1.4 Catalysis S11

1.5 H/D Exchange Experiments S12

1.6 Functionalization and Regeneration Cycle S14

1.7 Variable temperature ^{1}H NMR of $[(^{DIPP}BDI)CaH]_2$ and $(^{DIPP}BDI)Al$ in methylcyclohexane-d_{14} S15

1.8 Kinetic Studies by NMR Spectroscopy S17

1.9 Single Crystal X-ray Diffraction S19

2. Computational Details S28

3. References S86

1. Supporting Experimental Data

1.1. General Experimental Procedures

All experiments were conducted under an inert nitrogen atmosphere using standard Schlenk and glovebox techniques (MBraun, Labmaster SP). Toluene, *n*-hexane and benzene were degassed with nitrogen, dried over activated aluminum oxide (Solvent Purification System: Pure Solv 400−4−MD, Innovative Technology) and stored over 3Å molecular sieves. o-, m-, p-Xylene, mesitylene and trimethyl(phenyl)silane were dried over calcium hydride, distilled under N_2 atmosphere and stored over molecular sieves 3Å. C_6D_6, methylcyclohexane-d_{14} and toluene-d_8 were dried over 3Å molecular sieves. BDI-H (BDI = HC{(Me)CN(2,6-$i$$Pr_2C_6H_3$)}$_2$),[S1] (^{DIPP}BDI)Al,[S2] [(^{DIPP}BDI)CaH]$_2$,[S3] (^{DIPP}BDI)ZnH[S4] and [(^{DIPP}BDI)MgH]$_2$[S5] were synthesized according to a literature procedure. NMR spectra were recorded with a Bruker Avance III HD 400 MHz or a Bruker Avance III HD 600 MHz spectrometer. The spectra were referenced to the respective residual signals of the deuterated solvents. Elemental analysis was performed with a Euro EA 3000 (Euro Vector) analyzer. All crystal structures have been measured on a SuperNova (Agilent) diffractometer with dual Cu and Mo microfocus sources and an Atlas S2 detector. GC/MS measurements were performed on a Thermo Scientific™ Trace™ 1310 gas chromatography system (carrier gas Helium) with detection by a Thermo Scientific™ ISQ™ LT Single Quadrupole mass spectrometer. A Phenomenex® Zebron™ ZB-5 column of the dimensions 0.25 mm x 30 m with a film thickness of 0.25 µm was used. The samples (1 µl) were injected with an Instant Connect-SSL Module in the split mode (Injector Temperature: 280 °C). Temperature programs were started at 40 °C followed by heating ramps, optimized for the separation problem, until 280°. Baseline separation of each analyte was achieved by choosing the different temperature programs. The molecular identity was confirmed by comparison with entries in the NIST/EPA/NIH mass spectral library (version 2.2, built June 10 2014).

1.2. Synthesis

Synthesis of (^{DIPP}BDI)Al(Ph)H

(^{DIPP}BDI)Al (110.6 mg, 0.249 mmol) and [(^{DIPP}BDI)CaH]$_2$ (11.3 mg, 12.3 µmol) were dissolved in 2 ml of benzene. The resulting orange red mixture was stirred at room temperature for one day. During this time the color of the solution changed to yellow. All volatiles were removed *in vacuo.* The resulting yellow foam was redissolved in a minimum amount of hexane (~500 µl) and crystallized at -30°C. The obtained yellow crystals were dried *in vacuo*. Yield: 67%,

87.6 mg, 0.168 mmol. The NMR data match those of material obtained by the literature known method.[S6]

^{1}H NMR (400 MHz, C_6D_6, 298K): δ/ppm 7.36 (d, $^{3}J_{HH}$ = 6.5 Hz, 2H, Ar*H*), 7.21-7.00 (m, 9H, Ar*H*, partly omitted by solvent signal), 4.98 (s, 1H, C*H*), 4.74 (bs, 1H, Al*H*), 3.63 (hept, $^{3}J_{HH}$ = 6.6 Hz, 2H, C*H*Me_2), 3.18 (hept, $^{3}J_{HH}$ = 6.6 Hz, 2H, C*H*Me_2), 1.63 (s, 6H, 2x C*Me*), 1.46 (d, $^{3}J_{HH}$ = 6.7 Hz, 6H, CHMe_2), 1.21 (d, $^{3}J_{HH}$ = 6.8 Hz, 6H, CHMe_2), 1.12 (d, $^{3}J_{HH}$ = 6.8 Hz, 6H, CHMe_2), 0.63 (d, $^{3}J_{HH}$ = 6.7 Hz, 6H, CHMe_2). ^{13}C{^{1}H} NMR (101 MHz, C_6D_6, 298K): δ/ppm 170.6 (2x *C*(CMe)=NAr), 144.8 (2x *C*-Ar), 144.4 (2x *C*-Ar), 140.4 (2x *C*-Ar), 138.1 (2x *C*H-Ar), 127.9 (*C*H-Ar), 127.6 (2x *C*H-Ar), 127.1 (2x *C*H-Ar), 124.8 (2x *C*H-Ar), 124.7 (2x *C*H-Ar), 97.4 (*C*H), 28.9 (2x *C*HMe_2), 28.6 (2x *C*HMe_2), 25.4 (CHMe_2), 24.7 (CHMe_2), 24.7 (CHMe_2), 24.1 (CHMe_2), 23.3 (2x C*Me*). C_{ipso} was not observed. ^{27}Al NMR (104 MHz, C_6D_6, 298K): δ/ppm not observed. Anal. Calcd. for $C_{35}H_{47}AlN_2$ (MW= 522.76 g/mol): C, 80.42; H, 9.06; N, 5.36. Found: C, 80.80; H, 9.48; N, 5.07.

Synthesis of (^{DIPP}BDI)Al(*m*-tolyl)H

(^{DIPP}BDI)Al (58.8 mg, 0.132 mmol) and [(^{DIPP}BDI)CaH]$_2$ (6.0 mg, 6.5 μmol) were dissolved in 2 ml of toluene. The resulting orange red mixture was stirred at room temperature for one day. During this time the color of the solution changed to yellow. All volatiles were removed *in vacuo*. The resulting yellow foam was redissolved in a minimum amount of hexane (~600 μl) and crystallized at -30°C. The obtained yellow crystals were dried *in vacuo*. Another batch of crystals could be obtained by reducing the volume of the mother liquor. Total yield: 61%, 42.8 mg, 0.080 mmol. The NMR data match those of material obtained by the literature known method.[S6]

^{1}H NMR (400 MHz, C_6D_6, 298K): δ/ppm (only major isomer will be described) 7.18-7.13 (m, 4H, Ar*H*, partly omitted by solvent signal)), 7.08-6.92 (m, 6H, Ar*H*), 4.97 (s, 1H, C*H*), 4.73 (bs, 1H, Al*H*), 3.63 (hept, $^{3}J_{HH}$ = 6.8 Hz, 2H, C*H*Me_2), 3.20 (hept, $^{3}J_{HH}$ = 6.7 Hz, 2H, C*H*Me_2), 2.04 (s, 3H, (CH_3)C_6H_4), 1.63 (s, 6H, 2x C*Me*), 1.46 (d, $^{3}J_{HH}$ = 6.8 Hz, 6H, CHMe_2), 1.21 (d, $^{3}J_{HH}$ = 6.9 Hz, 6H, CHMe_2), 1.12 (d, $^{3}J_{HH}$ = 6.9 Hz, 6H, CHMe_2), 0.66 (d, $^{3}J_{HH}$ = 6.7 Hz, 6H, CHMe_2). ^{13}C{^{1}H} NMR (101 MHz, C_6D_6, 298K): δ/ppm (only major isomer will be described) 170.5 (2x *C*(CMe)=NAr), 144.7 (2x *C*-Ar), 144.5 (2x *C*-Ar), 140.5 (2x *C*-Ar), 139.1 (*C*H-Ar), 135.4 (*C*-Ar), 135.2 (*C*H-Ar), 128.4 (*C*H-Ar, omitted by solvent signal), 127.5 (2x *C*H-Ar), 127.0 (*C*H-Ar), 124.8 (2x *C*H-Ar), 124.7 (2x *C*H-Ar), 97.4 (*C*H), 28.8 (2x *C*HMe_2), 28.7 (2x *C*HMe_2), 25.5 (CHMe_2), 24.7 (CHMe_2), 24.7 (CHMe_2), 24.0 (CHMe_2), 23.3 (2x C*Me*), 21.6 ((*Me*)C_6H_4). C_{ipso} was not observed. ^{27}Al NMR (104 MHz, C_6D_6, 298K): δ/ppm not observed. Anal. Calcd. for $C_{36}H_{49}AlN_2$ (MW= 536.78 g/mol): C, 80.55; H, 9.20; N, 5.22. Found: C, 80.48; H, 9.27; N, 4.94.

Synthesis of (^{DIPP}BDI)Al(2,5-dimethylphenyl)H

(^{DIPP}BDI)Al (108.0 mg, 0.243 mmol) and [(^{DIPP}BDI)CaH]$_2$ (11.0 mg, 12.0 μmol) were dissolved in 2 ml of toluene. The resulting orange red mixture was stirred at room temperature for one day. During this time the color of the solution changed to yellow. All volatiles were removed *in vacuo*. The resulting yellow foam was redissolved in a minimum amount of hexane (~800 μl) and crystallized at -30°C. The obtained yellow crystals were dried *in vacuo*. Another batch of crystals could be obtained by reducing the volume of the mother liquor. Total yield: 69%, 92.2 mg, 0.167 mmol. The NMR data match those of material obtained by the literature known method.[S6]

^{1}H NMR (400 MHz, C_6D_6, 298K): δ/ppm 7.50 (s, 1H, Ar*H*), 7.15-7.07 (m, 5H, Ar*H*), 7.01-6.95 (m, 3H, Ar*H*), 4.97 (s, 1H, C*H*), 4.60 (bs, 1H, Al*H*), 3.58 (hept, $^{3}J_{HH}$ = 6.8 Hz, 2H, C*H*Me$_2$), 3.14 (hept, $^{3}J_{HH}$ = 6.7 Hz, 2H, C*H*Me$_2$), 2.63 (s, 3H, (CH$_3$)C$_6$H$_3$(C*H$_3$*)), 1.97 (s, 3H, (C*H$_3$*)C$_6$H$_3$(CH$_3$)), 1.56 (s, 6H, 2x C*Me*), 1.50 (d, $^{3}J_{HH}$ = 6.8 Hz, 6H, CH*Me$_2$*), 1.19 (d, $^{3}J_{HH}$ = 6.9 Hz, 6H, CH*Me$_2$*), 1.11 (d, $^{3}J_{HH}$ = 6.9 Hz, 6H, CH*Me$_2$*), 0.43 (d, $^{3}J_{HH}$ = 6.7 Hz, 6H, CH*Me$_2$*). ^{13}C{^{1}H} NMR (101 MHz, C_6D_6, 298K): δ/ppm 170.2 (2x *C*(CMe)=NAr), 145.5 (2x *C*-Ar), 144.1 (2x *C*-Ar), 142.3 (*C*-Ar), 140.9 (*C*H-Ar), 140.3 (2x *C*-Ar), 132.7 (*C*-Ar), 129.1 (*C*H-Ar), 128.7 (*C*H-Ar), 127.6 (2x *C*H-Ar), 124.9 (2x *C*H-Ar), 124.7 (2x *C*H-Ar), 97.6 (*C*H), 29.1 (2x *C*HMe$_2$), 28.0 (2x *C*HMe$_2$), 25.8 ((Me)C$_6$H$_3$(*Me*)), 25.1 (CH*Me$_2$*), 24.9 (CH*Me$_2$*), 24.8 (CH*Me$_2$*), 24.0 (CH*Me$_2$*), 23.4 (2x C*Me*), 20.9 ((*Me*)C$_6$H$_3$(Me)). C_{ipso} was not observed. ^{27}Al NMR (104 MHz, C_6D_6, 298K): δ/ppm not observed. Anal. Calcd. for $C_{37}H_{51}AlN_2$ (MW= 550.81 g/mol): C, 80.68; H, 9.33; N, 5.09. Found: C, 80.37; H, 9.39; N, 4.97.

Synthesis of (^{DIPP}BDI)Al(H)Zn(^{DIPP}BDI)

(^{DIPP}BDI)Al (95.3 mg, 0.214 mmol) and (^{DIPP}BDI)ZnH (104.5 mg, 0.216 mmol) were dissolved in 5 ml of benzene. The resulting orange red mixture was stirred at room temperature over night. During this time the color of the solution changed to yellow. All volatiles were removed *in vacuo*. The resulting yellow residue was redissolved in 7 ml of toluene, layered with hexane and stored at -30°C. The obtained yellow crystals were dried *in vacuo*. These crystals contained one molecule of co-crystalized toluene and were used for elemental analysis but were not sufficient for X-ray analysis. The solvent free product could be obtained by redissolving these crystals in benzene and removing all volatiles *in vacuo* in 82% yield (164.1 mg, 176.7 mmol). Crystals suitable for X-ray analysis could be obtained by storing a saturated solution of [(^{DIPP}BDI)Al(H)Zn(^{DIPP}BDI)] in hexane at -30°C.

^{1}H NMR (600 MHz, C_6D_6, 298K): δ/ppm 7.31 (dd, $^{3}J_{HH}$ =7.6 Hz, $^{4}J_{HH}$ =1.5 Hz, 1H, Ar*H*), 7.26-7.18 (m, 3H, Ar*H*), 7.15-7.00 (m, 6H, Ar*H*), 6.93 (dd, $^{3}J_{HH}$ =7.3 Hz, $^{4}J_{HH}$ =1.7 Hz, 1H, Ar*H*), 6.86 (dd, $^{3}J_{HH}$ =7.0 Hz, $^{4}J_{HH}$ =1.9 Hz, 1H, Ar*H*), 4.92 (s, 1H, C*H*), 4.66 (s, 1H, C*H*), 4.20 (bs, 1H, Al*H*), 4.04 (hept, $^{3}J_{HH}$ = 6.9 Hz, 1H, C*H*Me$_2$), 3.62-3.49 (m, 3H, 3x C*H*Me$_2$), 3.26 (hept,

$^3J_{HH}$ = 6.6 Hz, 1H, C*H*Me$_2$), 3.09 (hept, $^3J_{HH}$ = 6.7 Hz, 1H, C*H*Me$_2$), 2.83 (hept, $^3J_{HH}$ = 6.8 Hz, 1H, C*H*Me$_2$), 2.77 (hept, $^3J_{HH}$ = 6.8 Hz, 1H, C*H*Me$_2$), 1.79 (s, 3H, C*Me*), 1.74 (d, $^3J_{HH}$ = 6.9 Hz, 3H, CH*Me$_2$*), 1.60 (s, 3H, C*Me*), 1.47 (d, $^3J_{HH}$ = 6.9 Hz, 3H, CH*Me$_2$*), 1.44 (d, $^3J_{HH}$ = 6.9 Hz, 3H, CH*Me$_2$*), 1.42 (s, 3H, C*Me*), 1.40 (d, $^3J_{HH}$ = 6.8 Hz, 3H, CH*Me$_2$*), 1.36 (d, $^3J_{HH}$ = 6.8 Hz, 3H, CH*Me$_2$*), 1.32 (s, 3H, C*Me*), 1.29 (d, $^3J_{HH}$ = 6.8 Hz, 3H, CH*Me$_2$*), 1.27 (d, $^3J_{HH}$ = 6.8 Hz, 3H, CH*Me$_2$*), 1.21 (d, $^3J_{HH}$ = 6.7 Hz, 3H, CH*Me$_2$*), 1.17 (d, $^3J_{HH}$ = 6.9 Hz, 3H, CH*Me$_2$*), 1.15 (d, $^3J_{HH}$ = 6.8 Hz, 3H, CH*Me$_2$*), 1.07 (d, $^3J_{HH}$ = 7.0 Hz, 3H, CH*Me$_2$*), 1.04 (d, $^3J_{HH}$ = 6.7 Hz, 3H, CH*Me$_2$*), 1.01 (d, $^3J_{HH}$ = 6.8 Hz, 3H, CH*Me$_2$*), 0.61 (d, $^3J_{HH}$ = 6.9 Hz, 3H, CH*Me$_2$*), 0.52 (d, $^3J_{HH}$ = 6.8 Hz, 3H, CH*Me$_2$*), 0.47 (d, $^3J_{HH}$ = 6.8 Hz, 3H, CH*Me$_2$*). ^{13}C{^{1}H} NMR (151 MHz, C_6D_6, 298K): δ/ppm 169.3, 169.3, 165.6, 165.1, 147.4, 145.6, 145.4, 145.3, 144.8, 144.4 (2x), 143.6, 142.0, 141.9, 141.0, 140.3, 128.6, 127.3, 126.9, 125.3, 125.0, 125.0, 124.8, 124.8, 124.6, 124.5, 124.1, 123.7, 122.7, 97.5, 97.2, 29.1, 28.7, 28.6, 28.3, 28.3, 28.2, 27.9, 27.9, 27.1, 26.4, 26.2, 25.6, 25.3, 25.2, 25.2, 25.1, 25.0, 25.0, 25.0, 24.4, 24.1, 24.0, 23.9 (2x), 23.6, 23.2, 22.9. ^{27}Al NMR (156 MHz, C_6D_6, 298K): δ/ppm not observed. Anal. Calcd. for $C_{65}H_{91}AlN_4Zn$(MW= 1020.83 g/mol): C, 76.48; H, 8.99; N, 5.49. Found: C, 76.94; H, 8.99; N, 5.13.

Synthesis of (^{DIPP}BDI)Al(H)Mg(^{DIPP}BDI)

(^{DIPP}BDI)Al (84.3 mg, 0.190 mmol) and [(^{DIPP}BDI)MgH]$_2$ (84.2 mg, 0.95 mmol) were suspended in 1 ml of benzene. The resulting orange red mixture was stirred at room temperature overnight. During this time all solid dissolved and the color of the solution changed to dark red. All volatiles were removed *in vacuo*. The resulting yellow residue was redissolved in 0.5 ml of toluene, layered with hexane and stored at -30°C for several days. The obtained red crystals were dried *in vacuo*. These crystals contained one molecule of co-crystalized toluene. The solvent free product could be obtained by dissolving these crystals in benzene and removing all volatiles *in vacuo* in 15% yield (24.7 mg, 27.8 mmol). Crystals suitable for x-ray analysis could be obtained by storing a saturated solution of (^{DIPP}BDI)Al(H)Mg(^{DIPP}BDI) in hexane at -30°C.

^{1}H NMR (600 MHz, C_6D_6, 258K): δ/ppm 7.27-7.18 (m, 5H, Ar*H*), 7.10 – 7.07 (m, 1H), 7.00-6.96 (m, 2H, Ar*H*), 6.90 (d, $^3J_{HH}$ = 7.4 Hz, 1H, Ar*H*), 6.84 (t, $^3J_{HH}$ = 7.6 Hz, 1H, Ar*H*), 6.80 (d, $^3J_{HH}$ = 7.5 Hz, 1H, Ar*H*), 6.76 (d, $^3J_{HH}$ = 7.4 Hz, 1H, Ar*H*), 4.90-4.76 (m, 2H, C*H*, Al*H*), 4.56 (s, 1H, C*H*), 3.86-3.74 (m, 2H, 2x C*H*Me$_2$), 3.66-3.56 (m, 2H, 2x C*H*Me$_2$), 3.24 (hept, $^3J_{HH}$ = 6.6 Hz, 1H, C*H*Me$_2$), 3.15-3.05 (m, 2H, 2x C*H*Me$_2$), 2.87 (hept, $^3J_{HH}$ = 6.6 Hz, 1H, C*H*Me$_2$), 1.80 (d, $^3J_{HH}$ = 6.7 Hz, 3H, CH*Me$_2$*), 1.71 (s, 3H, C*Me*), 1.56 (d, $^3J_{HH}$ = 6.8 Hz, 3H, CH*Me$_2$*), 1.48 (d, $^3J_{HH}$ = 6.5 Hz, 3H, CH*Me$_2$*), 1.45 (s, 3H, C*Me*), 1.42 (d, $^3J_{HH}$ = 6.7 Hz, 3H, CH*Me$_2$*), 1.39 (s, 3H, C*Me*), 1.35 (d, $^3J_{HH}$ = 6.4 Hz, 6H, 2x CH*Me$_2$*), 1.30 (s, 3H, C*Me*), 1.24-1.20 (m, 9H, 3x CH*Me$_2$*), 1.12 (d, $^3J_{HH}$ = 6.9 Hz, 3H, CH*Me$_2$*), 1.08 (d, $^3J_{HH}$ = 6.7 Hz, 3H, CH*Me$_2$*), 1.05

(d, $^3J_{HH}$ = 6.7 Hz, 3H, CH*Me*$_2$), 1.00 (d, $^3J_{HH}$ = 6.8 Hz, 3H, CH*Me*$_2$), 0.83 (d, $^3J_{HH}$ = 7.7 Hz, 3H, CH*Me*$_2$), 0.81 (d, $^3J_{HH}$ = 7.2 Hz, 3H, CH*Me*$_2$), 0.63 (d, $^3J_{HH}$ = 6.6 Hz, 3H, CH*Me*$_2$). ^{13}C{^{1}H} NMR (151 MHz, C_6D_6, 258K): δ/ppm 168.7, 168.2, 168.1, 168.1, 146.6, 145.7, 144.9 (2x), 144.8, 144.7, 143.6, 143.5, 142.8, 142.1, 141.4, 141.3, 127.2, 127.1, 126.1, 125.8, 125.6, 125.3, 124.9, 124.8, 124.7, 124.4, 124.3, 123.7, 97.1, 97.0, 30.1, 29.1, 29.0, 29.0, 28.9, 28.8, 28.8, 28.7, 28.4, 28.0, 27.7, 26.3, 25.9, 25.6, 25.6 (2x), 25.5, 25.5, 25.4, 25.3, 24.9, 24.9, 24.7, 24.6, 24.1, 24.0, 23.8, 23.6. ^{27}Al NMR (156 MHz, C_6D_6, 258K): δ/ppm not observed. Anal. Calcd. for $C_{65}H_{91}AlMgN_4$ (MW= 979.76 g/mol): C, 79.68; H, 9.36; N, 5.72. Found: C, 79.39; H, 9.48; N, 5.39.

1.3. NMR Spectra of Synthesized Compounds

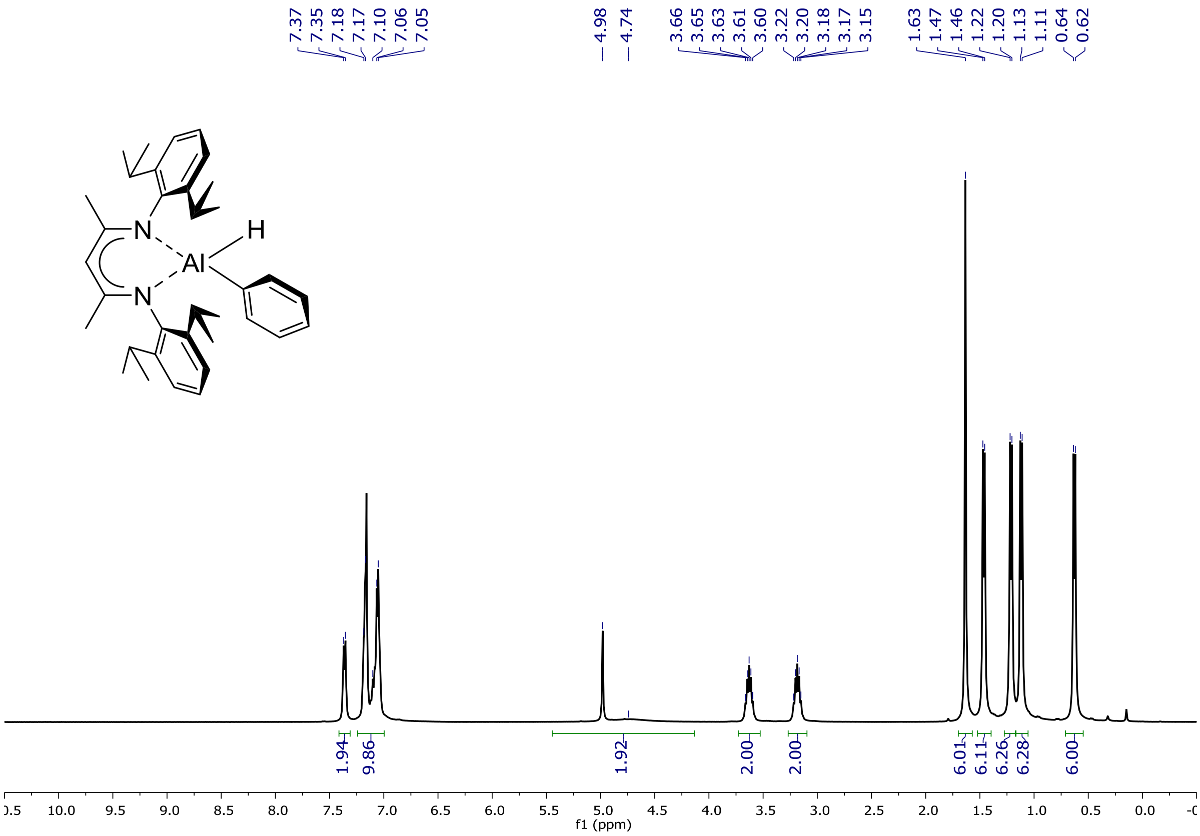

Figure S1: ^{1}H NMR spectrum of (^{DIPP}BDI)Al(Ph)H in C_6D_6 at 298 K.

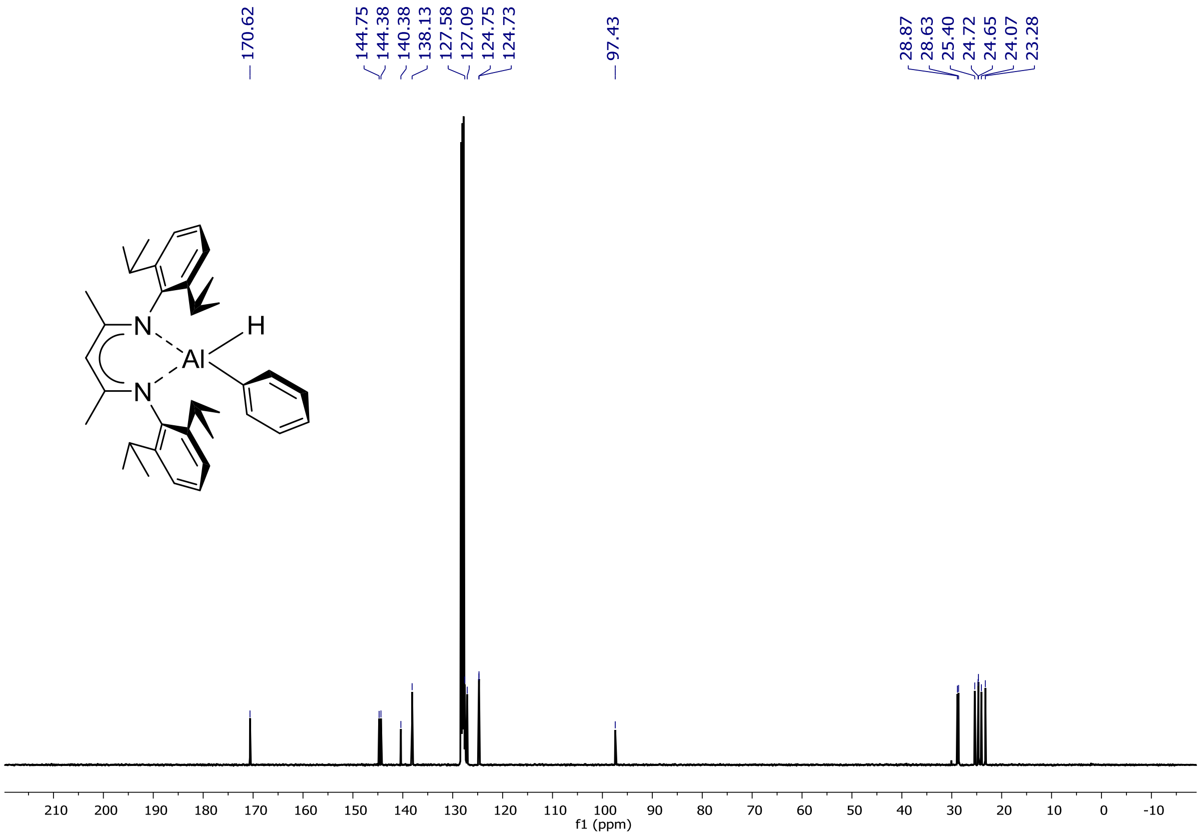

Figure S2: $^{13}C\{^{1}H\}$ NMR spectrum of (^{DIPP}BDI)Al(Ph)H in C_6D_6 at 298 K.

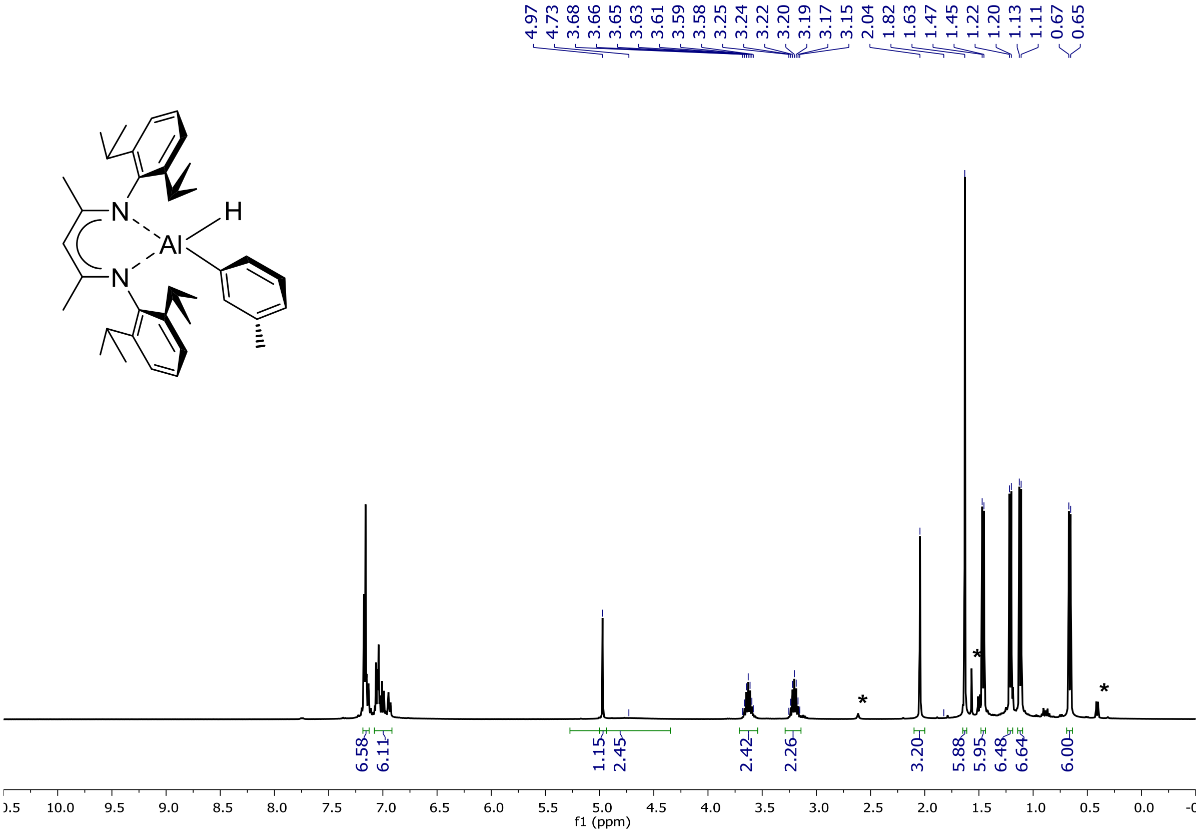

Figure S3: ^{1}H NMR spectrum of (^{DIPP}BDI)Al(m-tolyl)H in C_6D_6 at 298 K. * indicates small amounts of the *o*-tolyl impurity.

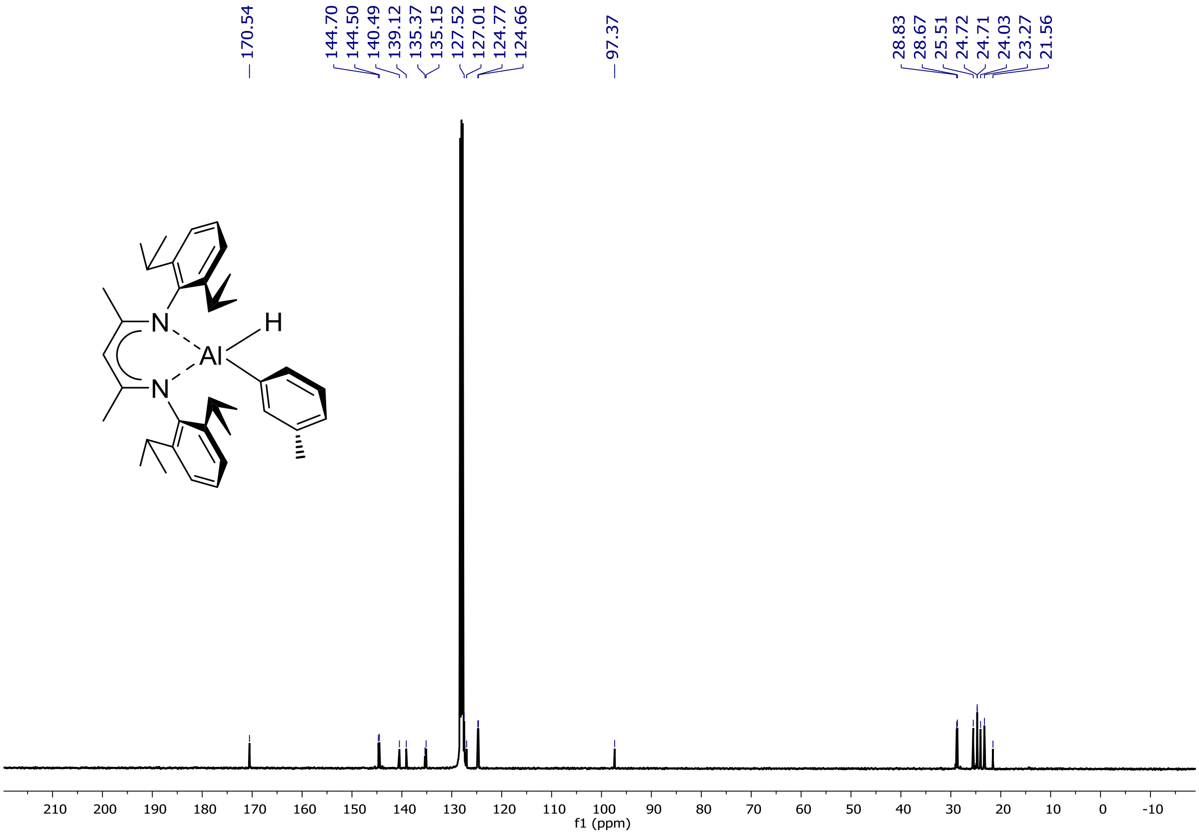

Figure S4: ^{13}C{^{1}H} NMR spectrum of (^{DIPP}BDI)Al(m-tolyl)H in C_6D_6 at 298 K.

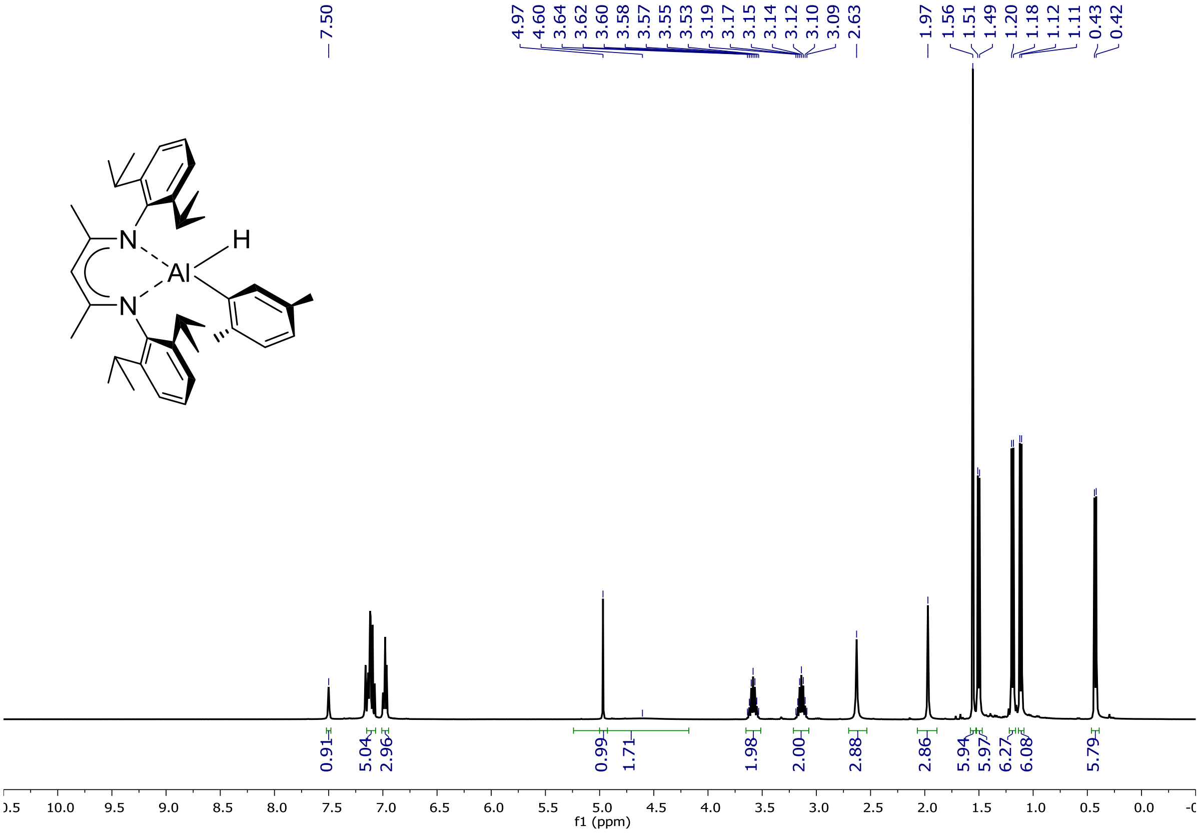

Figure S5: ^{1}H NMR spectrum of (^{DIPP}BDI)Al(2,5-dimethylphenyl)H in C_6D_6 at 298 K.

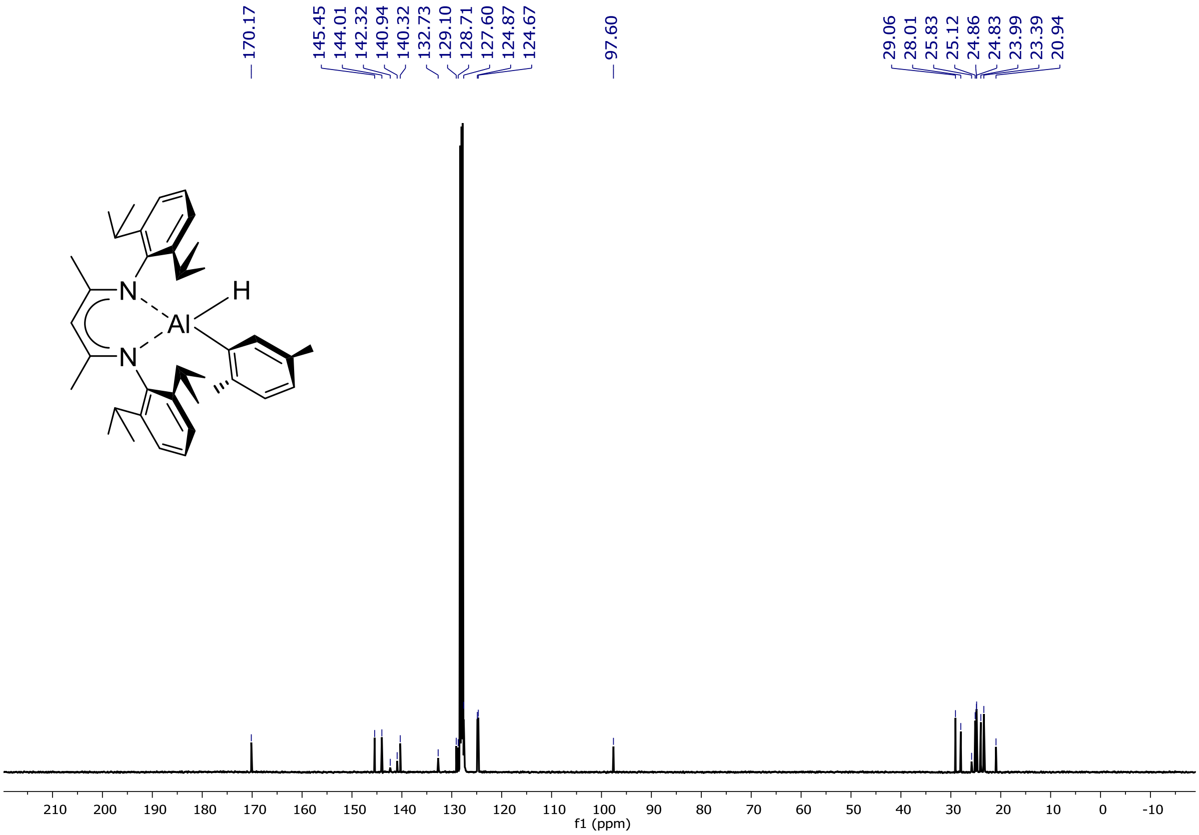

Figure S6: $^{13}C\{^{1}H\}$ NMR spectrum of (^{DIPP}BDI)Al(2,5-dimethylphenyl)H in C_6D_6 at 298 K.

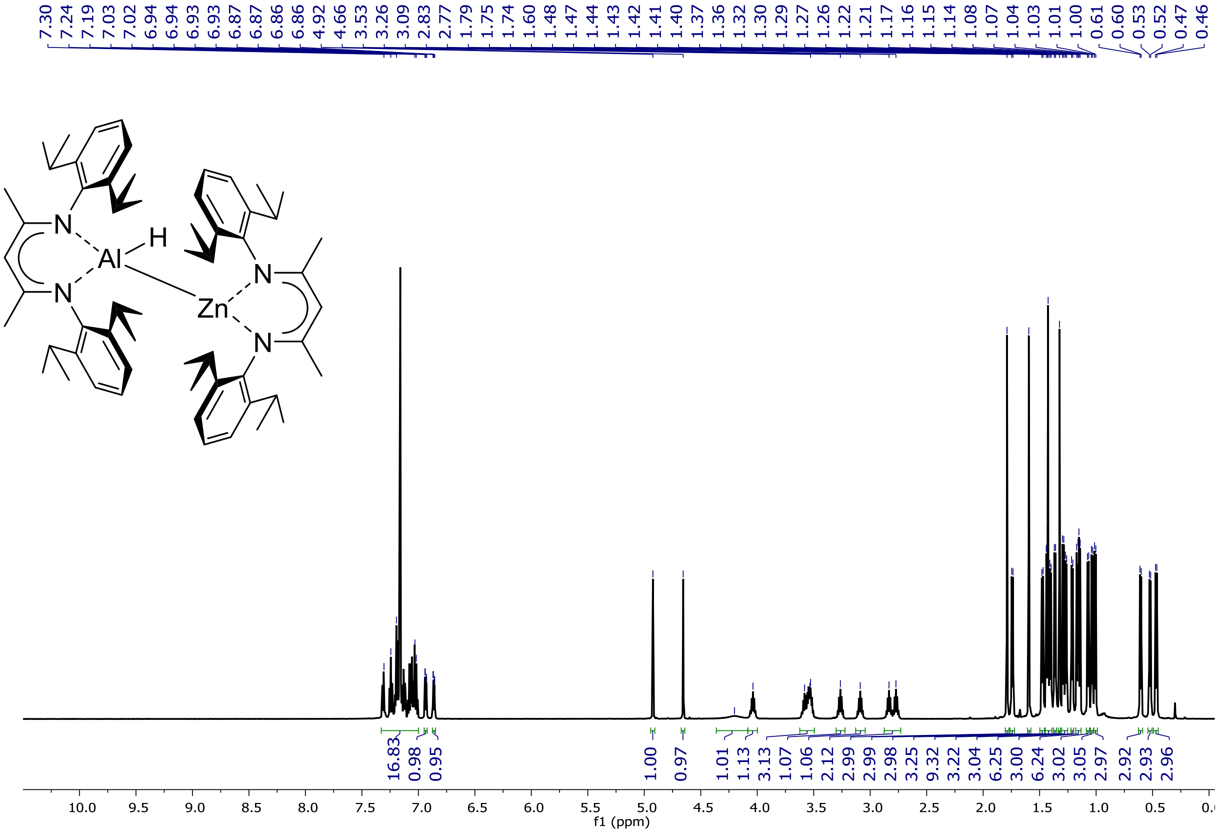

Figure S7: ^{1}H NMR spectrum of (^{DIPP}BDI)Al(H)Zn(^{DIPP}BDI) in C_6D_6 at 298 K.

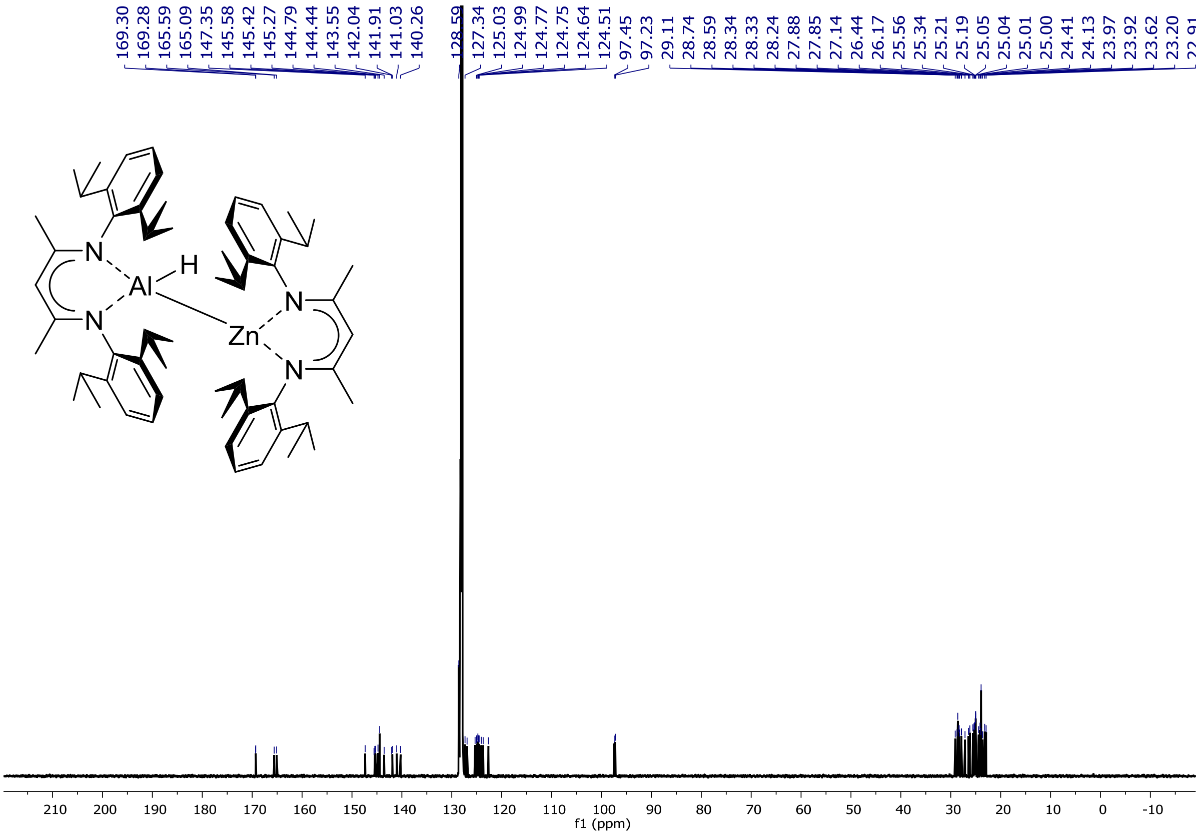

Figure S8: $^{13}C\{^{1}H\}$ NMR spectrum of (^{DIPP}BDI)Al(H)Zn(^{DIPP}BDI) in C_6D_6 at 298 K.

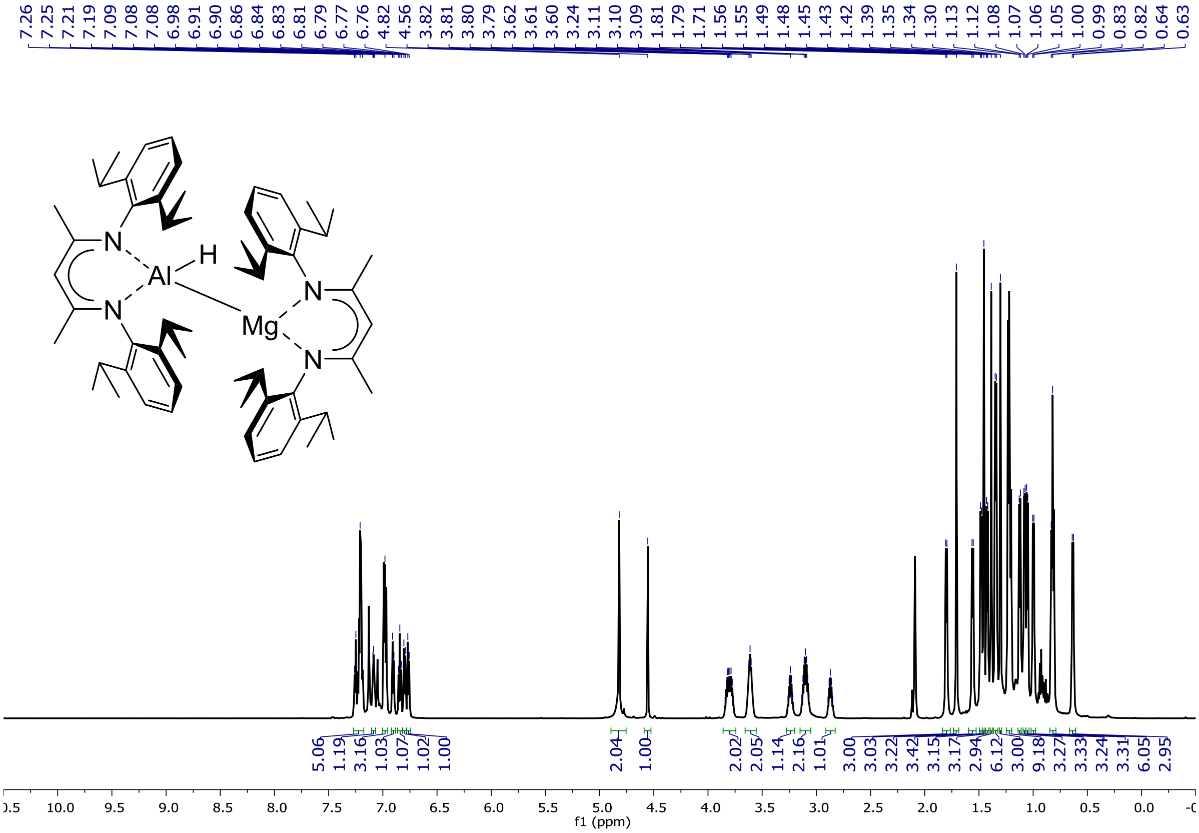

Figure S9: ^{1}H NMR spectrum of (^{DIPP}BDI)Al(H)Mg(^{DIPP}BDI) in toluene-d$_8$ at 258 K.

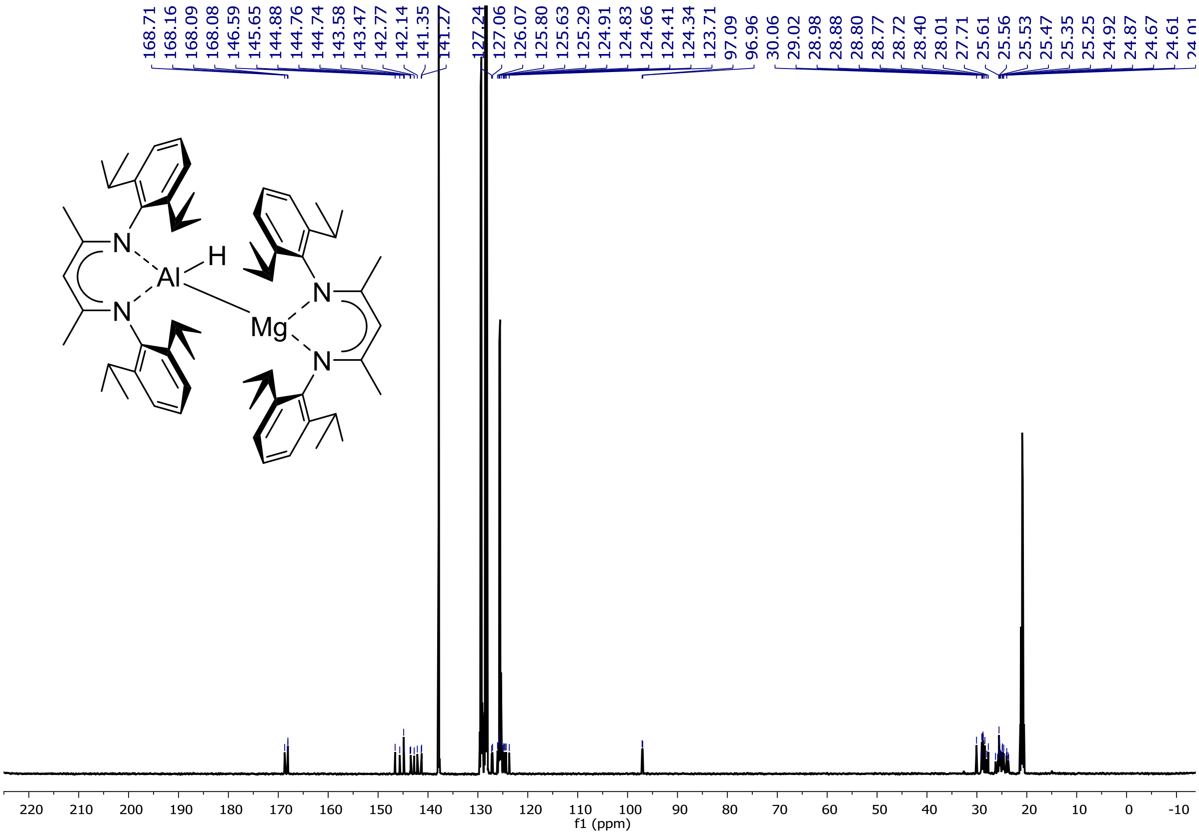

Figure S10: ^{13}C NMR spectrum of (^{DIPP}BDI)Al(H)Mg(^{DIPP}BDI) in toluene-d$_8$ at 258 K.

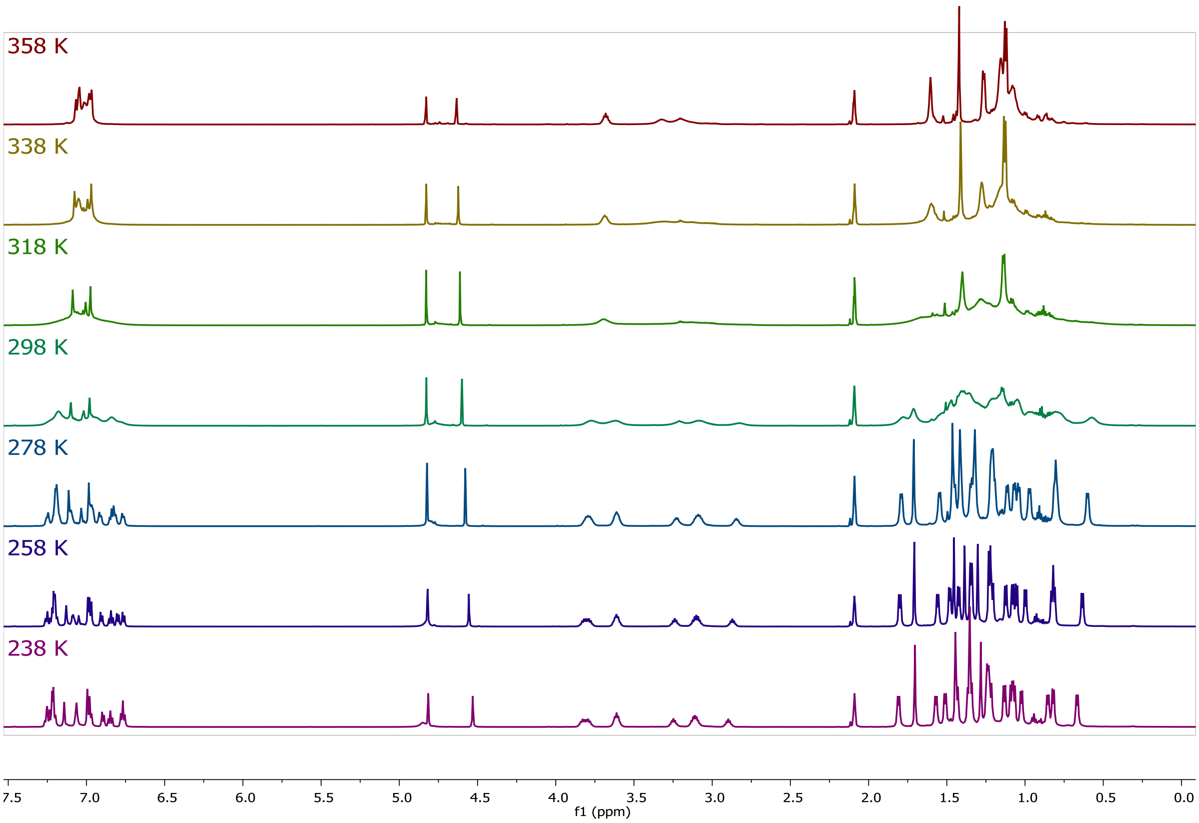

Figure S11: Variable temperature ^{1}H NMR of (^{DIPP}BDI)Al(H)Mg(^{DIPP}BDI) in toluene-d_8.

1.4 Catalysis

10.9 mM stock solutions of [(^{DIPP}BDI)CaH]$_2$ in the dry substrates were prepared (substrates were used as solvent). The desired quantity of the [(^{DIPP}BDI)CaH]$_2$ catalyst stock solution (2.5 mol%: 46.4 μL, 5 mol%: 92.8 μL, 7.5 mol%: 139.3 μL) was added to a solution of (^{DIPP}BDI)Al (9.0 mg, 20.2 μmol) in the substrate used as a solvent (2.5 mol%: 454 μL, 5 mol%: 407 μL, 7.5 mol%: 361 μL). The reaction was followed by proton NMR in 30 min intervals.

1.5 H/D Exchange Experiments

A solution of (^{DIPP}BDI)Al(C_6D_5)D (7.0 mg, 0.013 mmol) and [(^{DIPP}BDI)CaH]$_2$ (0.7 mg, 0.002 mmol) in 0.5 ml C_6H_6 were heated to 60°C for 3.5 days. The reaction was followed by ^{1}H and ^{2}D NMR spectroscopy. A control experiment without [(^{DIPP}BDI)CaH]$_2$ was done under the same conditions and also followed by ^{1}H and ^{2}D NMR spectroscopy.

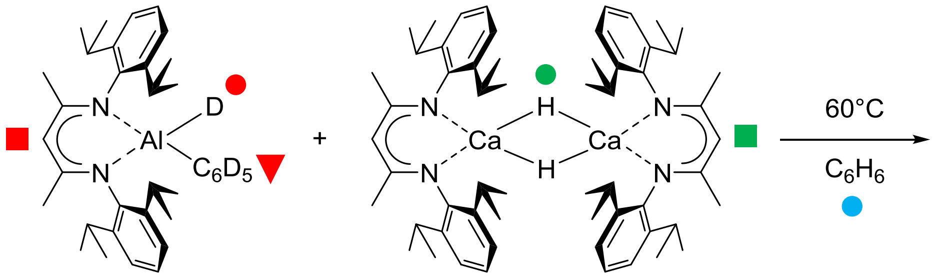

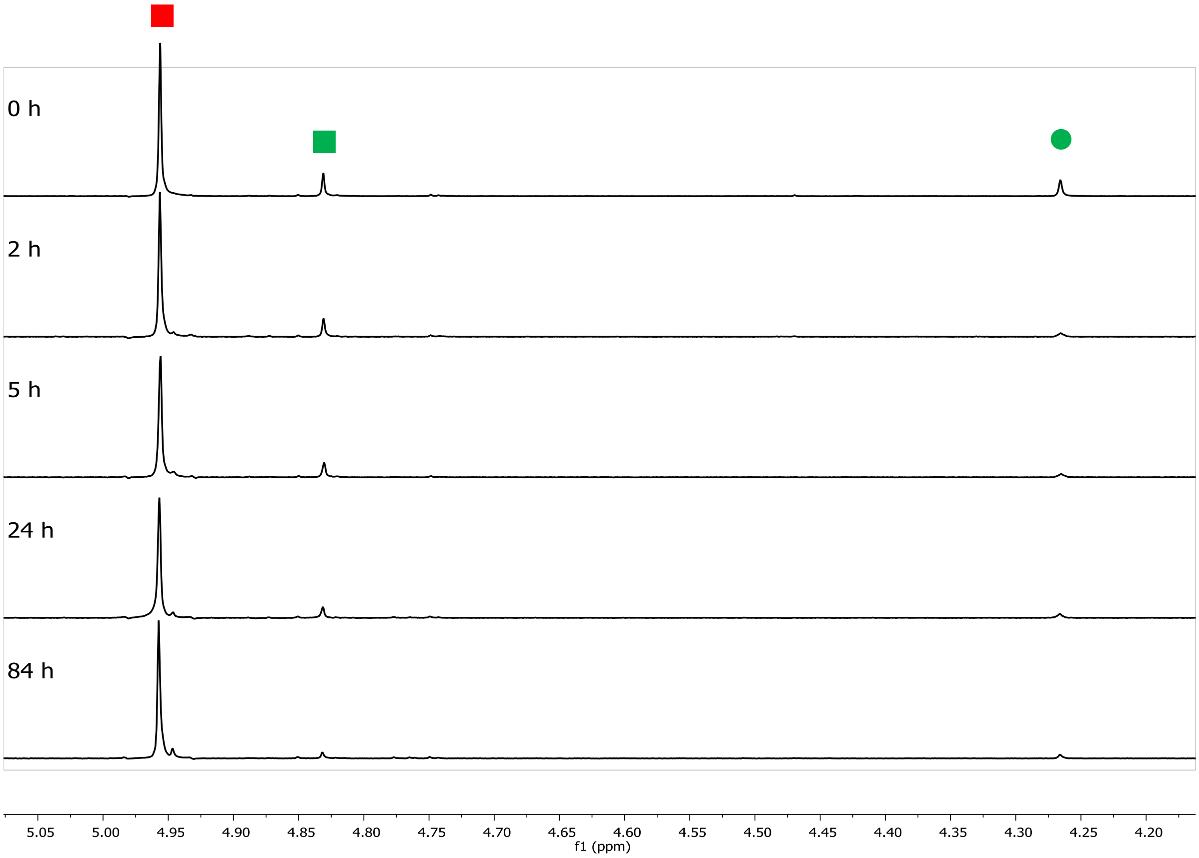

Figure S12: ^{1}H NMR spectra of (^{DIPP}BDI)Al(C_6D_5)D and [(^{DIPP}BDI)CaH]$_2$ in C_6H_6.

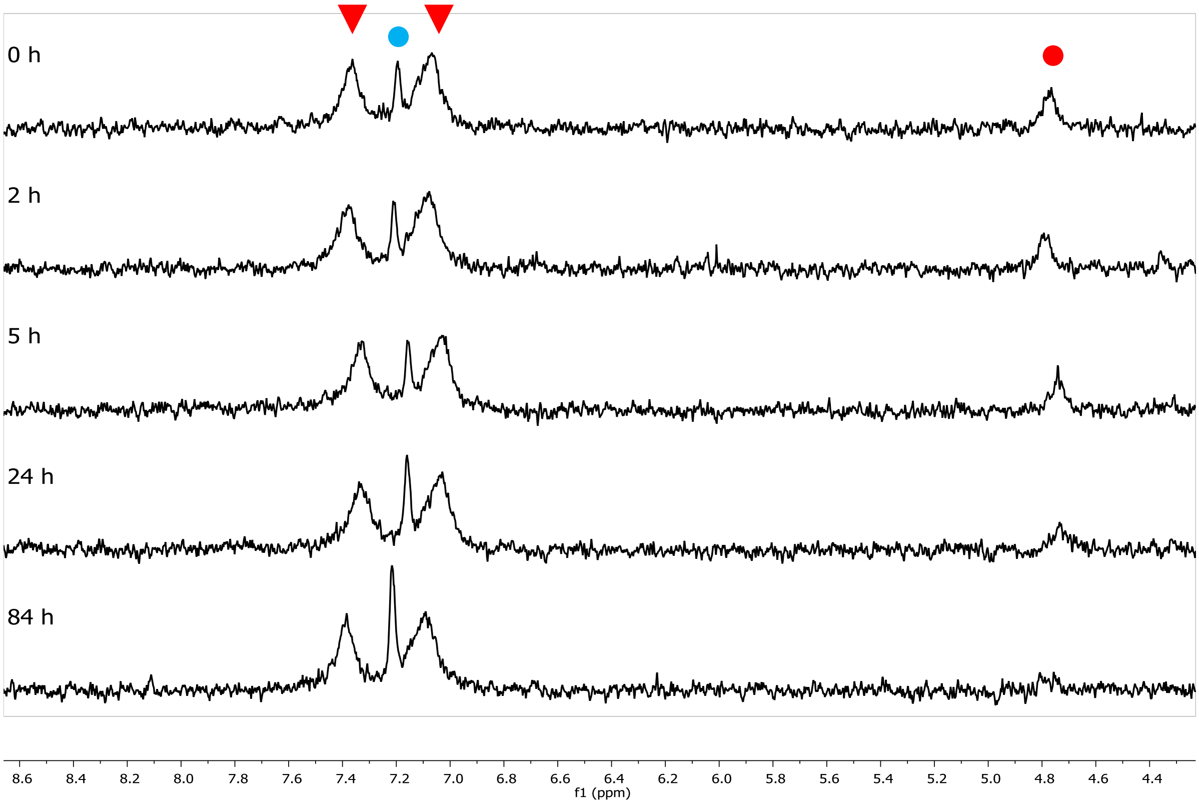

Figure S13: ^{2}D NMR spectra of (^{DIPP}BDI)Al(C_6D_5)D and [(^{DIPP}BDI)CaH]$_2$ in C_6H_6 (with natural abundancy of D).

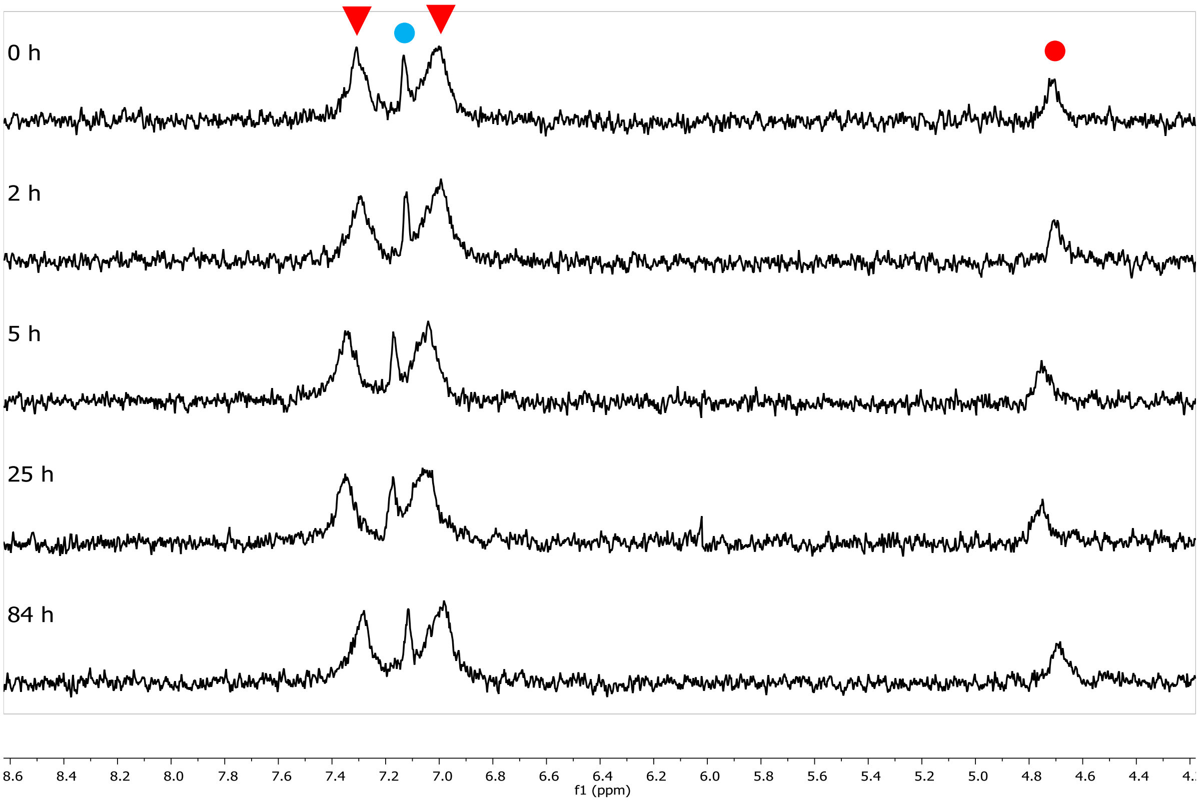

Figure S14: ^{2}D NMR spectra of (^{DIPP}BDI)Al(C_6D_5)D in C_6H_6 (with natural abundancy of D).

1.6 Functionalization and Regeneration Cycle

Solid (^{DIPP}BDI)Al(*m*-tolyl)H (3.8 mg, 0.007 mmol) was carefully placed in a J. Young-tube with a solution of I_2 (3.6 mg, 0.014 mmol) in 0.5 ml C_6D_6 and the tube was sealed before both components were mixed. Upon mixing an evolution of gas was observed and a ^{1}H NMR spectrum was recorded after 10 min. The sample was then heated to reflux for 24 h and another ^{1}H NMR was measured. Comparison of the chemical shifts showed the formation of (^{DIPP}BDI)AlI_2 and *m*-tolyl iodide. The latter was also identified by GC/MS analysis.

***m*-Tolyl iodide.** GC–MS (EI-MS, 70eV): m/z (%): RT: 6.57 min: 218.02 (49) [M^+], 91.11 (100), 65.10 (52).

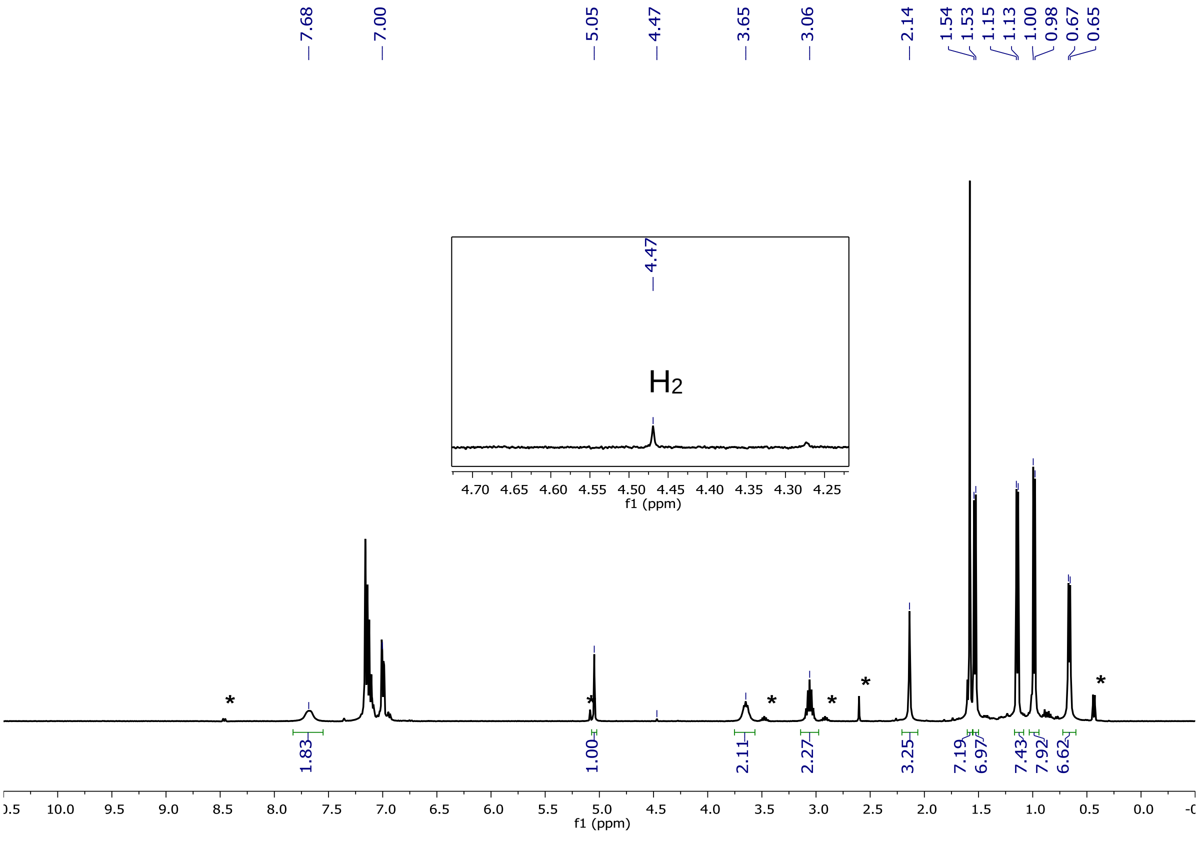

Figure S15: ^{1}H NMR spectrum 10 min after the addition of (^{DIPP}BDI)Al(m-tolyl)H to I_2 in C_6D_6. * indicates small amounts of the *o*-tolyl impurity. Clean conversion to (^{DIPP}BDI)Al(m-tolyl)I is observed.

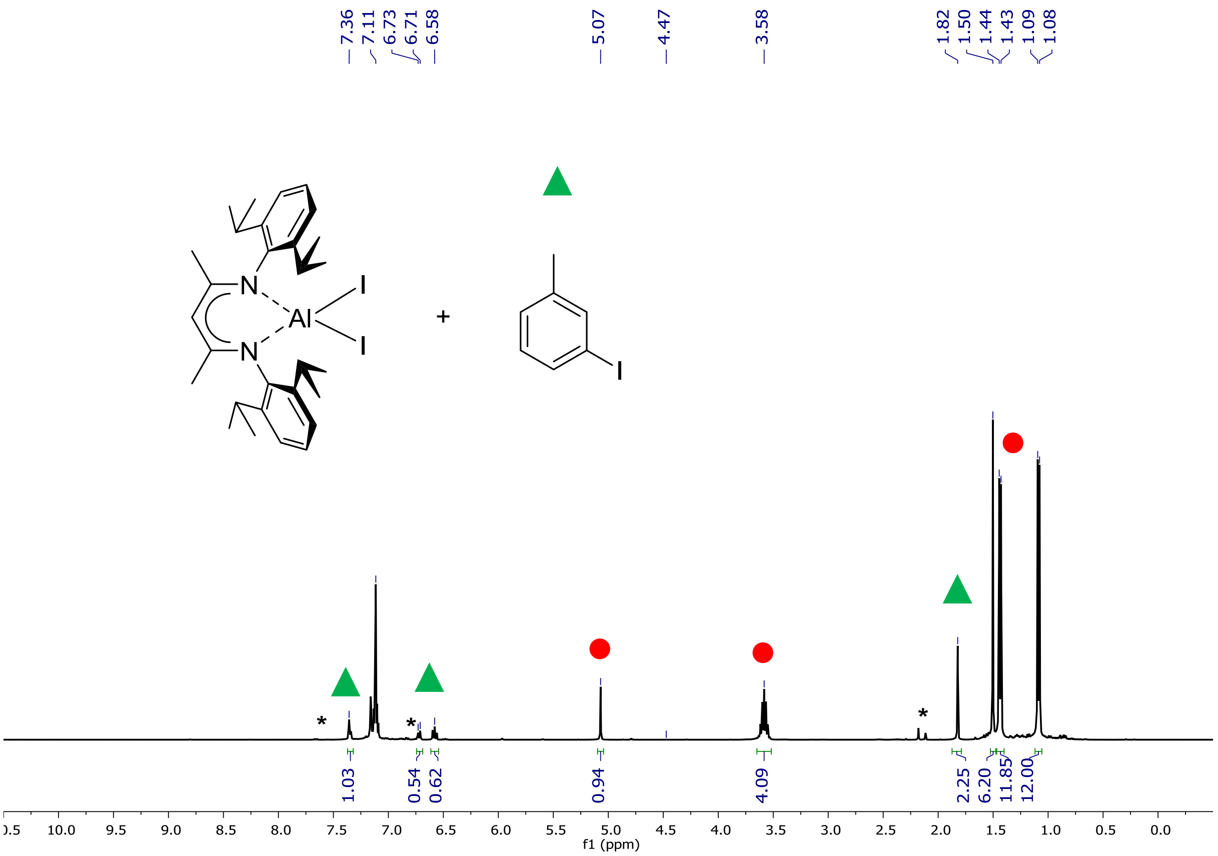

Figure S16: ^{1}H NMR spectrum after 24 h in refluxing C_6D_6. The complex (^{DIPP}BDI)Al(m-tolyl)I is fully converted to (^{DIPP}BDI)AlI_2. * indicates small amounts of the *o*-tolyl impurity.

1.7 Variable temperature ^{1}H NMR of [(^{DIPP}BDI)CaH]$_2$ and (^{DIPP}BDI)Al in methylcyclohexane-d$_{14}$

[(^{DIPP}BDI)CaH]$_2$ (1.5 mg, 1.6 μmol) and (^{DIPP}BDI)Al (1.4 mg, 3.1 μmol) were dissolved in 0.5 mL methylcyclohexane-d$_{14}$ and a ^{1}H NMR spectrum of the resulting mixture was recorded at room temperature. The sample was then cooled to 223 K and another ^{1}H NMR was measured. The sample was then allowed to warm to room temperature and another ^{1}H NMR spectrum was recorded.

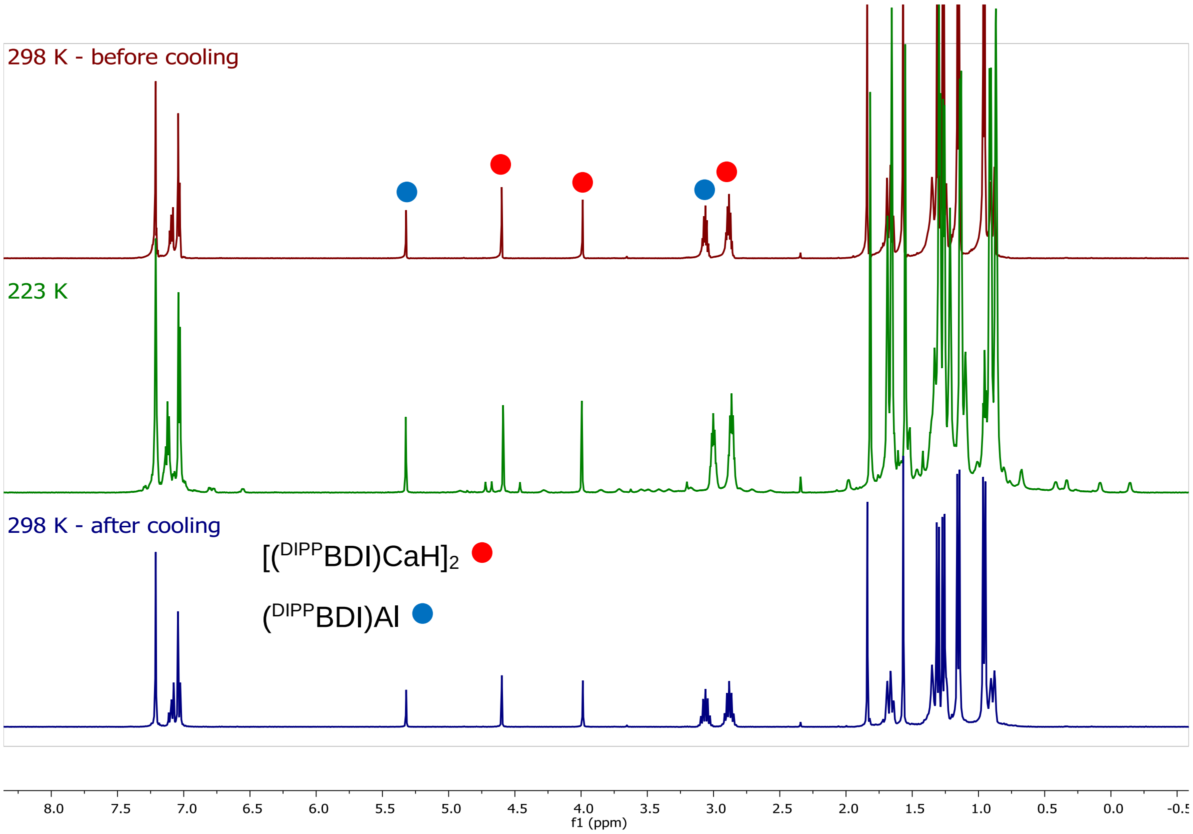

Figure S17: Variable temperature ^{1}H NMR of (^{DIPP}BDI)Al and [(^{DIPP}BDI)CaH]$_2$ in methylcyclohexane-d$_{14}$.

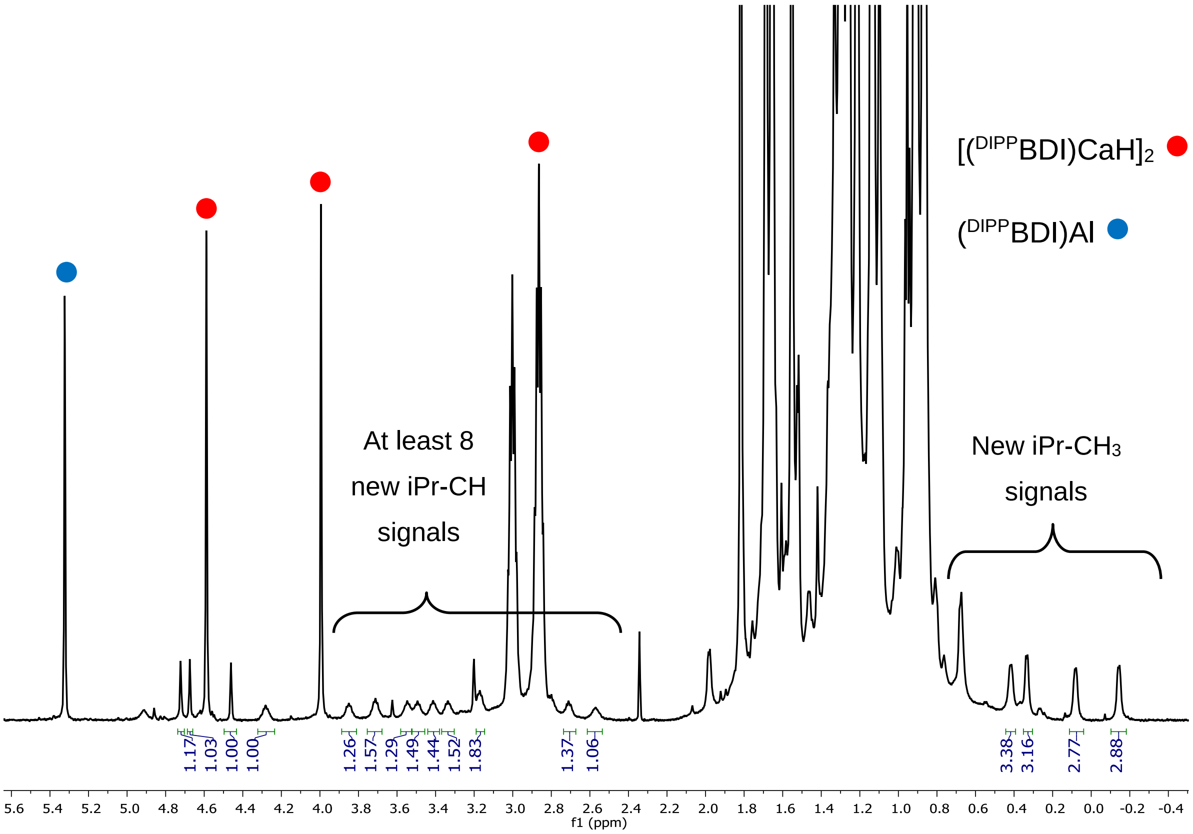

Figure S18: ^{1}H NMR spectrum of (^{DIPP}BDI)Al and [(^{DIPP}BDI)CaH]$_2$ in methylcyclohexane-d$_{14}$ at 223 K.

1.8 Kinetic Studies by NMR Spectroscopy

A 10.9 mM stock solution of [(^{DIPP}BDI)CaH]$_2$ in dry C_6H_6 was prepared. A quantity of 53.7 µl of the [(^{DIPP}BDI)CaH]$_2$ stock solution was added to a solution (^{DIPP}BDI)Al (5.2 mg, 11.7 µmol) in C_6H_6 (446 µl) and thoroughly mixed. The mixture was then transferred to a J. Young NMR tube containing a capillary insert standard (trimethyl(phenyl)silane in C_6D_6). The sealed tube was transported to the NMR spectrometer and the first proton NMR spectrum was recorded circa 5 min after mixing. The reaction was followed by proton NMR in 30 min steps at 25°C.

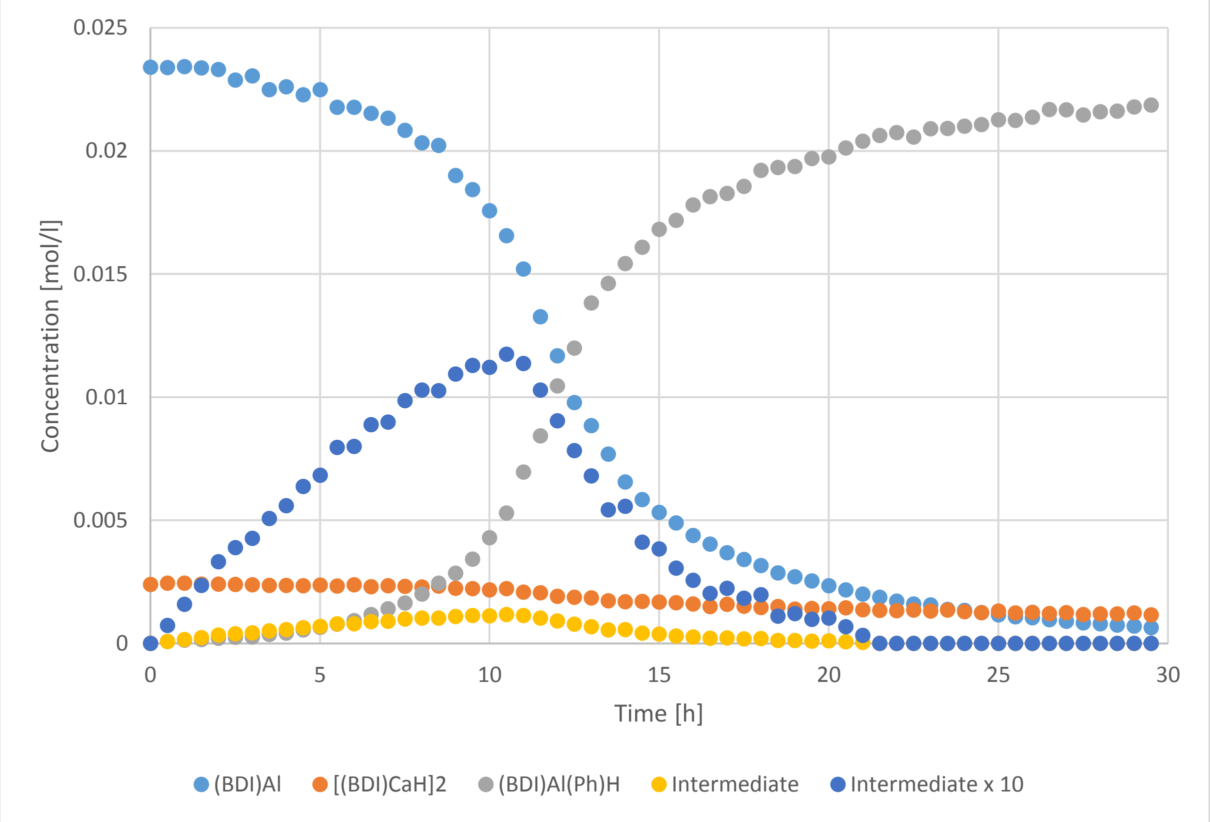

Figure S19: Conversion vs. time plot for the [(^{DIPP}BDI)CaH]$_2$ catalyzed reaction of (^{DIPP}BDI)Al with benzene at 20 °C. Intermediate x 10 represents the intermediate concentration multiplied by a factor of 10.

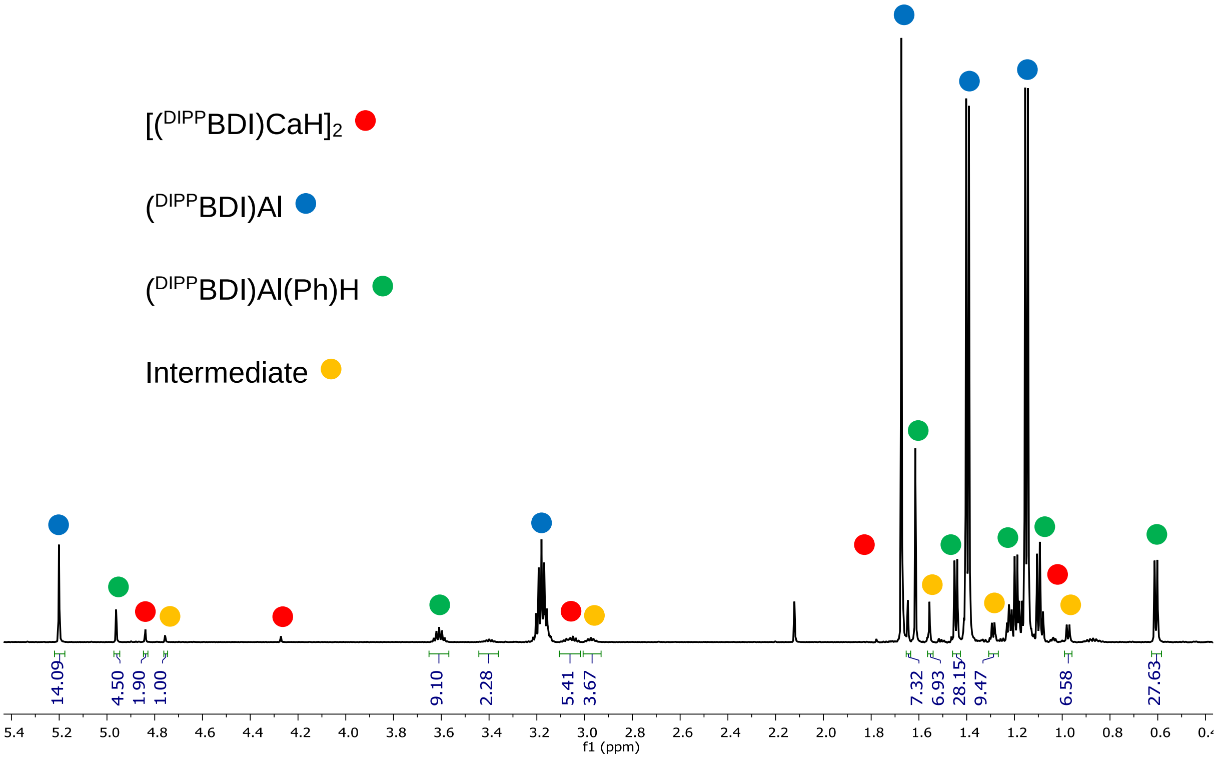

Figure S20: ^{1}H NMR spectrum of the reaction mixture 10.5 h for the $[(^{DIPP}BDI)CaH]_2$ catalyzed reaction of $(^{DIPP}BDI)Al$ with benzene at 20 °C.

1.9 Single Crystal X-Ray Diffraction

Structure Determination of (^{DIPP}BDI)Al(Ph)H

A colorless crystal of (^{DIPP}BDI)Al(Ph)H was embedded in inert perfluoropolyalkylether (viscosity 1800 cSt; ABCR GmbH) and mounted using a Hampton Research CryoLoop. The crystal was then flash cooled to 100.0(1) K in a nitrogen gas stream and kept at this temperature during the experiment. The crystal structure was measured on a SuperNova diffractometer with Atlas S2 detector using a CuKα microfocus source. The measured data was processed with the CrysAlisPro (v40.18b) software package.[S7] Using Olex2,[S8] the structure was solved with the ShelXT [S9] structure solution program using Intrinsic Phasing and refined with the ShelXL [S10] refinement package using Least Squares minimization. Except of the hydride at aluminum, all hydrogen atoms were placed in ideal positions and refined as riding atoms with relative isotropic displacement parameters. The position of the hydride was observed from difference Fourier maps and refined.

Disorder of one isopropyl group of the ligand over two positions was observed, which was modeled with the help of similarity restraints (SADI) and rigid bond restraints (RIGU [S11]). The relative contributions of the two alternative orientations were refined to 0.874(7) and 0.126(7).

Table S1 contains a summary of crystal data and refinement details. The crystal structure data has been deposited with the Cambridge Crystallographic Data Centre. CCDC 1941420 contains the supplementary crystallographic data for complex (^{DIPP}BDI)Al(Ph)H. This data can be obtained free of charge from The Cambridge Crystallographic Data Centre via www.ccdc.cam.ac.uk/data_request/cif.

Structure Determination of (^{DIPP}BDI)Al(*m*-tolyl)H

A colorless crystal of (^{DIPP}BDI)Al(*m*-tolyl)H was embedded in inert perfluoropolyalkylether (viscosity 1800 cSt; ABCR GmbH) and mounted using a Hampton Research CryoLoop. The crystal was then flash cooled to 100.0(1) K in a nitrogen gas stream and kept at this temperature during the experiment. The crystal structure was measured on a SuperNova diffractometer with Atlas S2 detector using a CuKα microfocus source. The measured data was processed with the CrysAlisPro (v40.18b) software package.[S7] Using Olex2,[S8] the structure was solved with the ShelXT [S9] structure solution program using Intrinsic Phasing and refined with the ShelXL [S10] refinement package using Least Squares minimization. Except of the hydride at aluminum, all hydrogen atoms were placed in ideal positions and refined as riding atoms with relative isotropic displacement parameters. The position of the hydride was observed from difference Fourier maps and refined.

In the final stages of the structure refinement it became obvious that the crystal under investigation contained a small amount of (^{DIPP}BDI)Al(o-tolyl)H as impurity. The resulting disorder of the tolyl group was modeled with the help of similarity restraints (SADI, SIMU) and rigid bond restraints (RIGU).[S11] The relative contributions of the two alternative orientations were refined to 0.912(4) (*m*-tolyl) and 0.088(4) (*o*-tolyl) .
Table S1 contains a summary of crystal data and refinement details. The crystal structure data has been deposited with the Cambridge Crystallographic Data Centre. CCDC 1941421 contains the supplementary crystallographic data for complex (^{DIPP}BDI)Al(*m*-tolyl)H. This data can be obtained free of charge from The Cambridge Crystallographic Data Centre via www.ccdc.cam.ac.uk/data_request/cif.

Structure Determination of (^{DIPP}BDI)Al(2,5-dimethylphenyl)H

A colorless crystal of (^{DIPP}BDI)Al(2,5-dimethylphenyl)H was embedded in inert perfluoropolyalkylether (viscosity 1800 cSt; ABCR GmbH) and mounted using a Hampton Research CryoLoop. The crystal was then flash cooled to 100.0(1) K in a nitrogen gas stream and kept at this temperature during the experiment. The crystal structure was measured on a SuperNova diffractometer with Atlas S2 detector using a CuKα microfocus source. The measured data was processed with the CrysAlisPro (v40.18b) software package.[S7] Using Olex2,[S8] the structure was solved with the ShelXT [S9] structure solution program using Intrinsic Phasing and refined with the ShelXL [S10] refinement package using Least Squares minimization. Most hydrogen atoms were placed in ideal positions and refined as riding atoms with relative isotropic displacement parameters. The positions of the hydride at aluminum and of the hydrogen atom at C11 were observed from difference Fourier maps and refined.
Disorder of co-crystallized *n*-hexane over two positions was observed, which was modeled with the help of similarity restraints (SADI, SIMU) and rigid bond restraints (RIGU [S11]). The relative contributions of the two alternative orientations were refined to 0.902(5) and 0.098(5).
Table S1 contains a summary of crystal data and refinement details. The crystal structure data has been deposited with the Cambridge Crystallographic Data Centre. CCDC 1941422 contains the supplementary crystallographic data for complex (^{DIPP}BDI)Al(2,5-dimethylphenyl)H. This data can be obtained free of charge from The Cambridge Crystallographic Data Centre via www.ccdc.cam.ac.uk/data_request/cif.

Structure Determination of (^{DIPP}BDI)Al(H)Zn(^{DIPP}BDI)

A yellow crystal of (^{DIPP}BDI)Al(H)Zn(^{DIPP}BDI) was embedded in inert perfluoropolyalkylether (viscosity 1800 cSt; ABCR GmbH) and mounted using a Hampton Research CryoLoop. The crystal was then flash cooled to 100.0(1) K in a nitrogen gas stream and kept at this temperature during the experiment. The crystal structure was measured on a SuperNova diffractometer with Atlas S2 detector using a CuKα microfocus source. The measured data was processed with the CrysAlisPro (v40.18b) software package.[S7] Using Olex2,[S8] the structure was solved with the ShelXT [S9] structure solution program using Intrinsic Phasing and refined with the ShelXL [S10] refinement package using Least Squares minimization. Except of the hydride at aluminum, all hydrogen atoms were placed in ideal positions and refined as riding atoms with relative isotropic displacement parameters. The position of the hydride was observed from difference Fourier maps and refined.
In the crystal, disorder of the central Zn-Al(H) fragment over two positions was observed. This induces additional disorder in 4 isopropyl groups and one methyl group, which was modeled with the help of similarity restraints (SADI, SIMU) and rigid bond restraints (RIGU [S11]). The relative contributions of the two alternative orientations of the molecule were refined to 0.8107(14) and 0.1893(14).
Table S2 contains a summary of crystal data and refinement details. The crystal structure data has been deposited with the Cambridge Crystallographic Data Centre. CCDC 1941423 contains the supplementary crystallographic data for complex (^{DIPP}BDI)Al(H)Zn(^{DIPP}BDI) This data can be obtained free of charge from The Cambridge Crystallographic Data Centre via www.ccdc.cam.ac.uk/data_request/cif.

Structure Determination of (^{DIPP}BDI)Al(H)Mg(^{DIPP}BDI)

A red crystal of (^{DIPP}BDI)Al(H)Mg(^{DIPP}BDI) was embedded in inert perfluoropolyalkylether (viscosity 1800 cSt; ABCR GmbH) and mounted using a Hampton Research CryoLoop. The crystal was then flash cooled to 100.0(1) K in a nitrogen gas stream and kept at this temperature during the experiment. The crystal structure was measured on a SuperNova diffractometer with Atlas S2 detector using a CuKα microfocus source. The measured data was processed with the CrysAlisPro (v40.18b) software package.[S7] Using Olex2,[S8] the structure was solved with the ShelXT [S9] structure solution program using Intrinsic Phasing and refined with the ShelXL [S10] refinement package using Least Squares minimization. Except of the hydride at aluminum, all hydrogen atoms were placed in ideal positions and refined as riding atoms with relative isotropic displacement parameters. The position of the hydride was observed from difference Fourier maps and refined.

Disorder of one isopropyl group of the ligand over two positions was observed and successfully modeled. The relative contributions of the two alternative orientations were refined to 0.703(15) and 0.297(15).

Table S2 contains a summary of crystal data and refinement details. The crystal structure data has been deposited with the Cambridge Crystallographic Data Centre. CCDC 1941424 contains the supplementary crystallographic data for complex (^{DIPP}BDI)Al(H)Mg(^{DIPP}BDI). This data can be obtained free of charge from The Cambridge Crystallographic Data Centre via www.ccdc.cam.ac.uk/data_request/cif

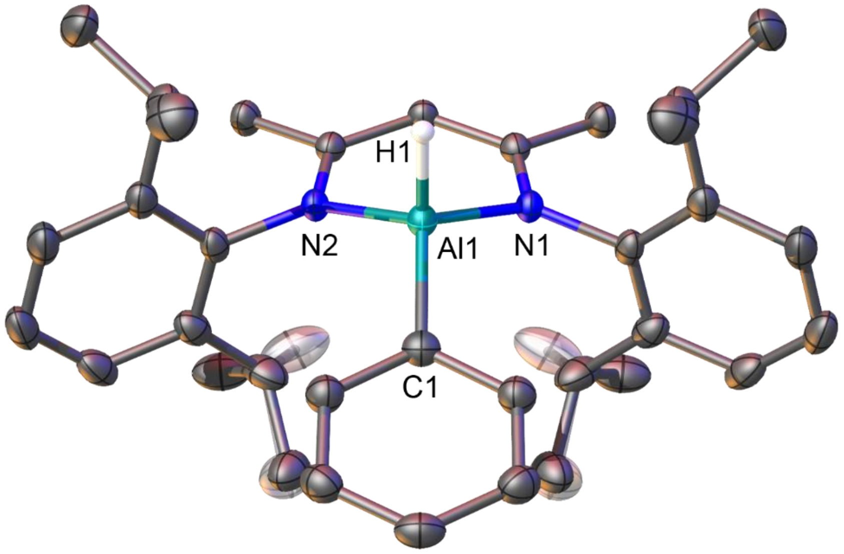

Figure S21: The crystal structure of (^{DIPP}BDI)Al(Ph)H (50% probability ellipsoids). The disorder is shown with 30% transparency.

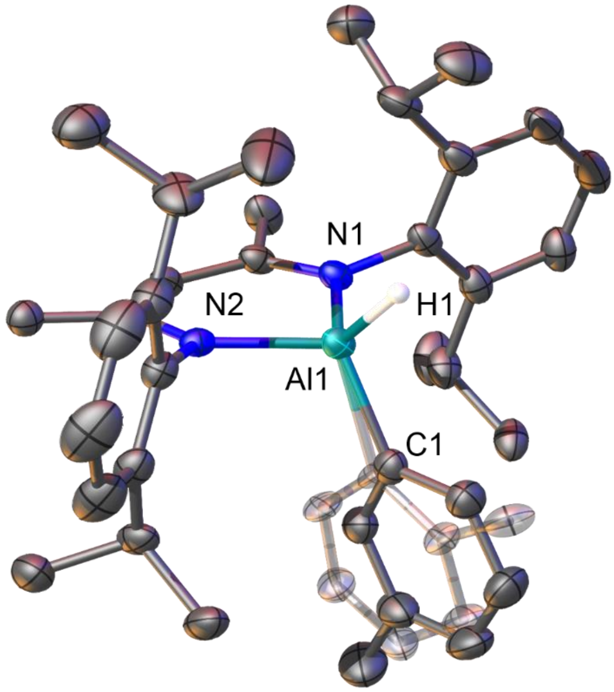

Figure S22: The crystal structure of (^{DIPP}BDI)Al(*m*-tolyl)H (50% probability ellipsoids). The disorder is shown with 30% transparency.

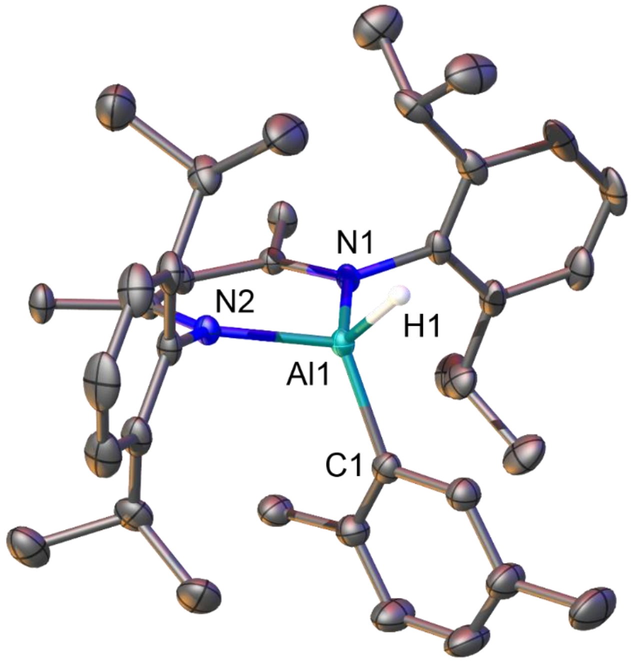

Figure S23: The crystal structure of (^{DIPP}BDI)Al(2,5-dimethylphenyl)H (50% probability ellipsoids).

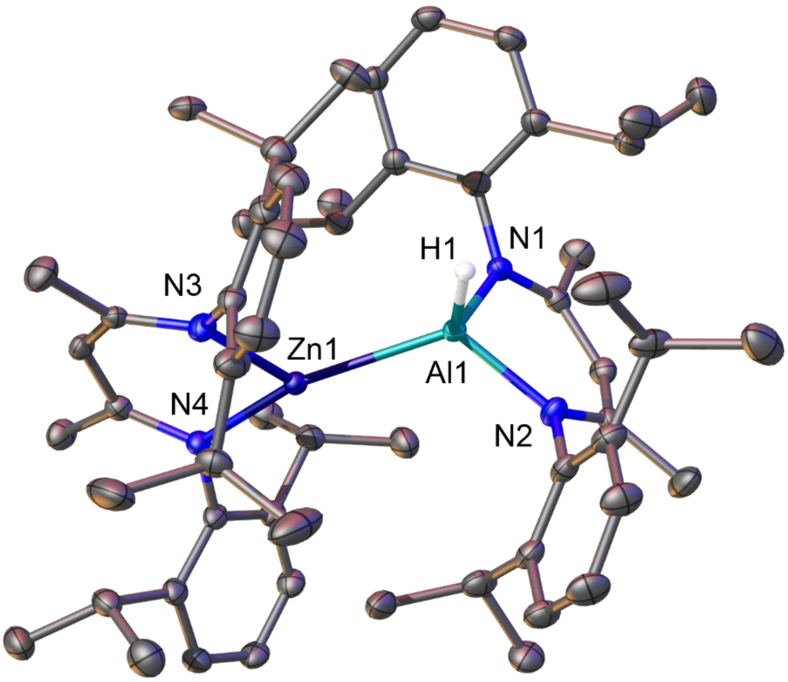

Figure S24: The crystal structure of (^{DIPP}BDI)Al(H)Zn(^{DIPP}BDI) (50% probability ellipsoids). The disorder is omitted for clarity.

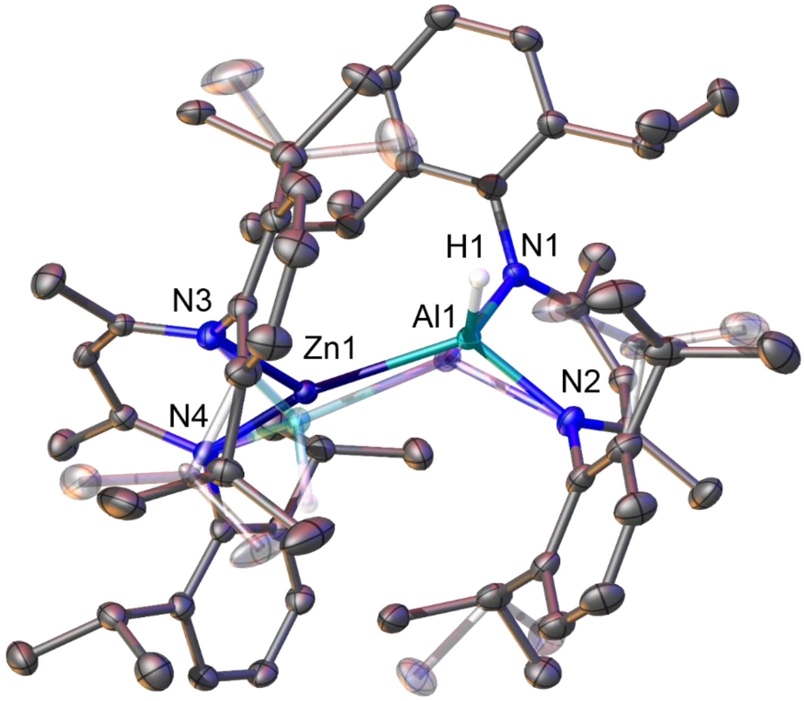

Figure S25: The crystal structure of (^{DIPP}BDI)Al(H)Zn(^{DIPP}BDI) (50% probability ellipsoids). The disorder is shown with 30% transparency.

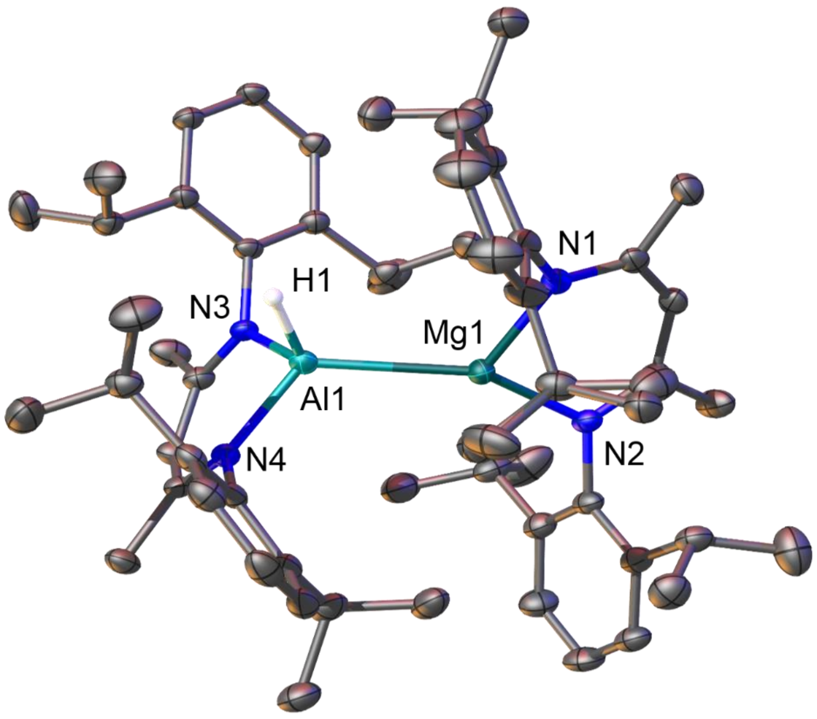

Figure S26: The crystal structure of (^{DIPP}BDI)Al(H)Mg(^{DIPP}BDI) (50% probability ellipsoids).

Table S1: Crystal data and structure refinement for compound (^{DIPP}BDI)Al(Ph)H, (^{DIPP}BDI)Al(m-tolyl)H and (^{DIPP}BDI)Al(2,5-dimethylphenyl)H.

**Identification code**	(^{DIPP}BDI)Al(Ph)H	(^{DIPP}BDI)Al(m-tolyl)H	(^{DIPP}BDI)Al(2,5-dimethylphenyl)H ·*n*-Hexane
**Empirical formula**	$C_{35}H_{47}AlN_2$	$C_{36}H_{49}AlN_2$	$C_{43}H_{65}AlN_2$
**Formula weight**	522.72	536.75	636.95
**Temperature/K**	100.0(1)	100.0(1)	100.0(1)
**Crystal system**	orthorhombic	orthorhombic	orthorhombic
**Space group**	Pnma	$Pna2_1$	Pnma
**a/Å**	16.5626(3)	16.6292(2)	14.5876(2)
**b/Å**	20.9046(3)	12.7285(2)	23.2280(4)
**c/Å**	9.01452(17)	15.5352(2)	11.8538(2)
**α/°**	90	90	90
**β/°**	90	90	90
**γ/°**	90	90	90
**Volume/Å^3**	3121.13(9)	3288.25(8)	4016.56(13)
**Z**	4	4	4
**ρ_{calc}g/cm^3**	1.112	1.084	1.053
**μ/mm^{-1}**	0.736	0.710	0.645
**F(000)**	1136.0	1168.0	1400.0
**Crystal size/mm^3**	0.516 × 0.218 × 0.118	0.196 × 0.176 × 0.087	0.274 × 0.063 × 0.054
**Radiation**	CuKα (λ = 1.54184)	CuKα (λ = 1.54184)	CuKα (λ = 1.54184)
**2Θ range for data collection/°**	8.46 to 147.13	8.748 to 145.844	8.374 to 147.372
**Index ranges**	-12 ≤ h ≤ 20, -24 ≤ k ≤ 25, -4 ≤ l ≤ 10	-20 ≤ h ≤ 20, -12 ≤ k ≤ 15, -19 ≤ l ≤ 19	-15 ≤ h ≤ 17, -17 ≤ k ≤ 27, -9 ≤ l ≤ 14
**Reflections collected**	7078	26881	9196
**Independent reflections**	3136 [R_{int} = 0.0242, R_{sigma} = 0.0288]	6454 [R_{int} = 0.0364, R_{sigma} = 0.0256]	3998 [R_{int} = 0.0278, R_{sigma} = 0.0294]
**Data/restraints/parameters**	3136/15/207	6454/585/432	3998/106/267
**Goodness-of-fit on F^2**	1.022	1.047	1.072
**Final R indexes [I>=2σ (I)]**	R_1 = 0.0406, wR_2 = 0.1082	R_1 = 0.0345, wR_2 = 0.0888	R_1 = 0.0437, wR_2 = 0.1185
**Final R indexes [all data]**	R_1 = 0.0447, wR_2 = 0.1126	R_1 = 0.0361, wR_2 = 0.0902	R_1 = 0.0531, wR_2 = 0.1248
**Largest diff. peak/hole / e Å^{-3}**	0.35/-0.26	0.14/-0.23	0.41/-0.36
**Flack parameter**	-	-0.08(2)	-
**CCDC number**	1941420	1941421	1941422

Table S2: Crystal data and structure refinement for compound (^{DIPP}BDI)Al(H)Zn(^{DIPP}BDI) and (^{DIPP}BDI)Al(H)Mg(^{DIPP}BDI).

**Identification code**	**(^{DIPP}BDI)Al(H)Zn(^{DIPP}BDI)**	**(^{DIPP}BDI)Al(H)Mg(^{DIPP}BDI)**
**Empirical formula**	$C_{58}H_{83}AlN_4Zn$	$C_{58}H_{83}AlMgN_4$
**Formula weight**	928.63	887.57
**Temperature/K**	100.0(1)	100.0(1)
**Crystal system**	triclinic	monoclinic
**Space group**	P-1	$P2_1/n$
**a/Å**	11.93991(19)	15.91598(13)
**b/Å**	12.63426(19)	17.44284(17)
**c/Å**	21.0771(3)	19.46920(16)
**α/°**	90.3215(11)	90
**β/°**	106.1832(13)	94.4631(7)
**γ/°**	111.8064(14)	90
**Volume/Å^3**	2813.71(8)	5388.65(8)
**Z**	2	4
**ρ_{calc}g/cm^3**	1.096	1.094
**μ/mm^{-1}**	1.029	0.727
**F(000)**	1004.0	1936.0
**Crystal size/mm^3**	0.47 × 0.248 × 0.183	0.297 × 0.182 × 0.141
**Radiation**	CuKα (λ = 1.54184)	CuKα (λ = 1.54184)
**2Θ range for data collection/°**	7.594 to 147.37	6.814 to 145.192
**Index ranges**	-14 ≤ h ≤ 14, -15 ≤ k ≤ 15, -26 ≤ l ≤ 25	-19 ≤ h ≤ 19, -19 ≤ k ≤ 21, -21 ≤ l ≤ 23
**Reflections collected**	42848	30507
**Independent reflections**	11140 [R_{int} = 0.0226, R_{sigma} = 0.0189]	10365 [R_{int} = 0.0244, R_{sigma} = 0.0251]
**Data/restraints/parameters**	11140/198/722	10365/0/622
**Goodness-of-fit on F^2**	1.107	1.033
**Final R indexes [I>=2σ (I)]**	R_1 = 0.0413, wR_2 = 0.0959	R_1 = 0.0414, wR_2 = 0.1070
**Final R indexes [all data]**	R_1 = 0.0432, wR_2 = 0.0965	R_1 = 0.0476, wR_2 = 0.1114
**Largest diff. peak/hole / e Å^{-3}**	0.36/-0.37	0.56/-0.44
**CCDC number**	1941423	1941424

2. Computational Details

General

All calculations were carried out using Gaussian 16A.[S12] All methods were used as implemented. All structures were fully optimized on a ωB97XD/6-31+G** level of theory.[S13-S15] All structures were characterized as true minima (Nimag=0) or transition states (Nimag=1) by frequency calculations on the same level of theory except the minima for (^{DIPP}BDI)Zn-Al(H)(^{DIPP}BDI), (^{DIPP}BDI)Mg-Al(H)(^{DIPP}BDI), (^{DIPP}BDI)Ca-Al(H)(^{DIPP}BDI) and (^{DIPP}BDI)Ca-Al(μ-H)(^{DIPP}BDI), for which frequency calculations were carried out at ωB97XD/6-31G* level of theory due to convergence issues at higher levels of theory. Energies were determined at a ωB97XD/6-311+G** level of theory. Solvation effects were approximated using a PCM field of benzene.[S16] Charges were calculated via NBO Analyses.[S17] We calculated three different pathways for the reaction between (^{Dipp}BDI)Al and benzene catalyzed by CaH_2. The latter Ca hydride species was used as a model system in order to save on calculational costs. Charges were calculated using NBO analyses. All structures were evaluated using Molecule 2.3.[S18] Topological analyses were carried out with AIMAll17 using the wave function of the optimization.[S19,S20]

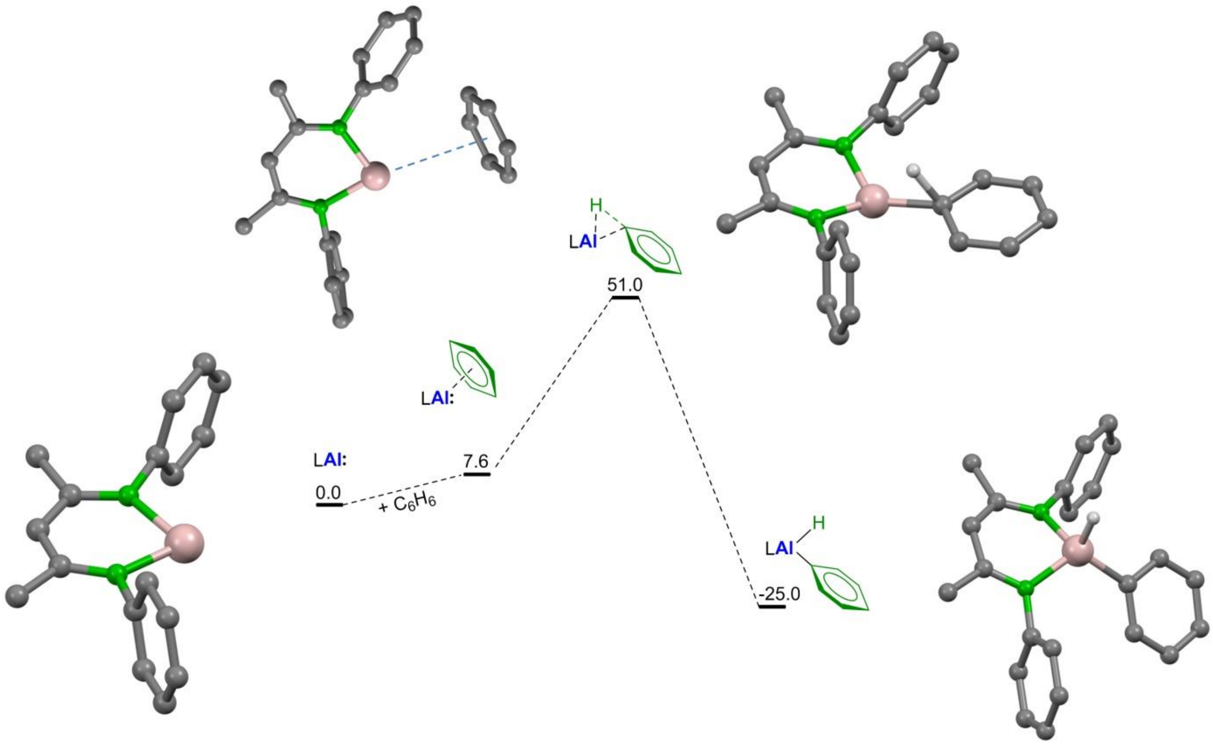

Figure S27: Direct oxidative addition of (^{DIPP}BDI)Al to the C-H bond in benzene. ωB97XD/6-311+G**//ωB97XD/6-31+G**, ΔG values at 298 K and 1 bar given in kcal/mol.

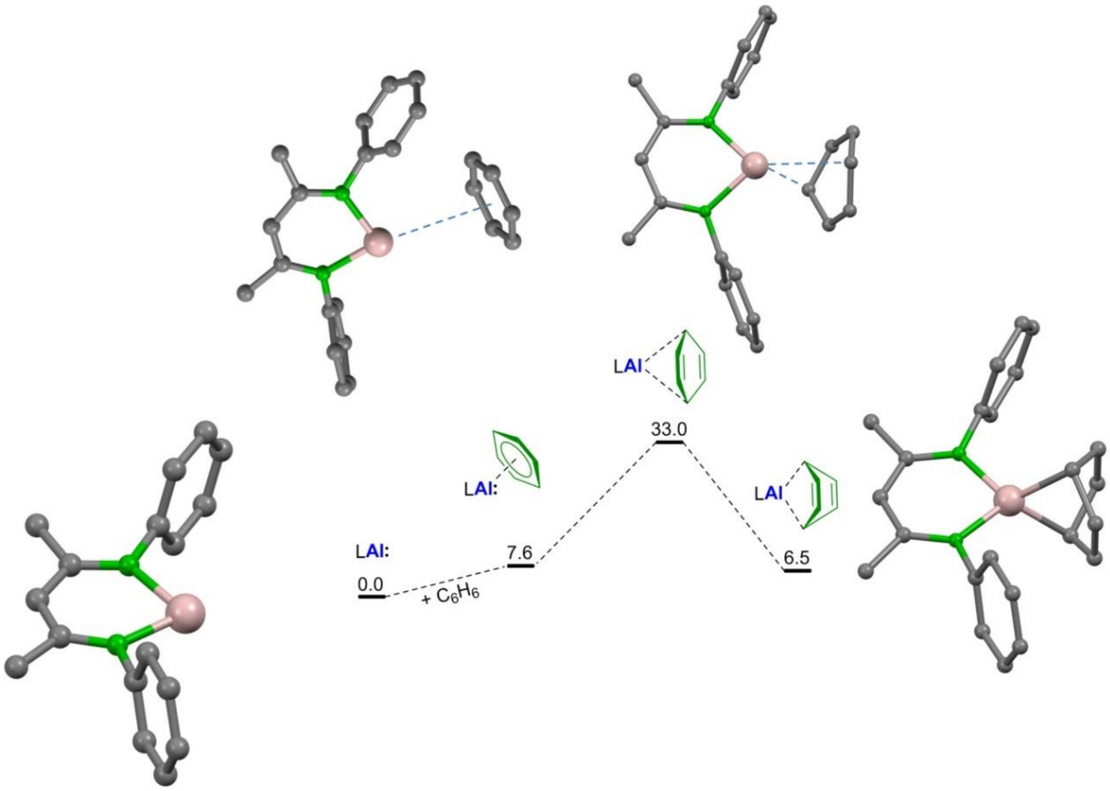

Figure S28: Reduction of benzene by (^{DIPP}BDI)Al. ωB97XD/6-311+G**//ωB97XD/6-31+G**, ΔG values at 298 K and 1 bar given in kcal/mol.

XYZ Coordinates

	147		
LMg-Al(H)L			
Al	1.335556	0.414239	-0.220647
H	2.315674	0.213481	1.045032
N	2.403876	-0.270397	-1.720891
N	1.564951	2.287567	-0.784651
C	3.308669	-0.307539	-4.002350
H	4.277745	-0.730466	-3.724813
H	3.447178	0.368646	-4.846447
H	2.681080	-1.146026	-4.318486
C	2.668099	0.406548	-2.835928
C	2.413351	1.776903	-2.981507
H	2.677612	2.218154	-3.934174
C	2.000842	2.673698	-1.976285
C	2.068668	4.143027	-2.318402
H	1.085080	4.493195	-2.644187
H	2.776067	4.315371	-3.130395
H	2.356645	4.744817	-1.454212
C	3.033922	-1.545507	-1.537073
C	4.334759	-1.566694	-0.992722
C	4.974930	-2.797348	-0.847577
H	5.981417	-2.829979	-0.439533
C	4.349779	-3.982225	-1.216620
H	4.866281	-4.930540	-1.103710
C	3.051469	-3.947886	-1.704671
H	2.552795	-4.879655	-1.957745
C	2.366735	-2.740254	-1.858243
C	5.049075	-0.292575	-0.564811
H	4.355174	0.540323	-0.698488
C	6.283506	-0.010855	-1.432204
H	7.027901	-0.809489	-1.337518
H	6.756476	0.927367	-1.123435
H	6.019855	0.079392	-2.490416
C	5.418599	-0.335264	0.922790
H	4.524695	-0.485597	1.534649
H	5.881649	0.612503	1.219017
H	6.134115	-1.136529	1.139534
C	0.919108	-2.758371	-2.318504
H	0.589390	-1.718393	-2.396389
C	0.741677	-3.417812	-3.690624
H	1.300556	-2.890507	-4.469841
H	-0.315379	-3.421463	-3.972875
H	1.083566	-4.458179	-3.682220
C	0.032349	-3.453378	-1.274783
H	0.283785	-4.515626	-1.180266
H	-1.027194	-3.386912	-1.546317

H	0.163344	-3.006317	-0.283757
C	1.161128	3.272188	0.178616
C	2.037977	3.597272	1.232109
C	1.630017	4.555292	2.163045
H	2.299931	4.828092	2.973121
C	0.389875	5.172276	2.068458
H	0.092309	5.916769	2.800528
C	-0.473056	4.820955	1.039329
H	-1.452339	5.288064	0.982466
C	-0.114625	3.866042	0.086341
C	3.421330	2.976209	1.353941
H	3.475266	2.141886	0.652204
C	4.512286	3.983002	0.964586
H	4.366596	4.351972	-0.056026
H	5.499825	3.511358	1.015518
H	4.515169	4.847578	1.638271
C	3.662320	2.395551	2.751661
H	3.653088	3.171589	3.525276
H	4.639253	1.903805	2.790527
H	2.898981	1.649911	2.992789
C	-1.126526	3.477211	-0.981161
H	-0.651482	2.767039	-1.663754
C	-1.618870	4.674762	-1.806836
H	-2.211340	5.362178	-1.193349
H	-2.259667	4.325619	-2.623044
H	-0.794231	5.249507	-2.237343
C	-2.329888	2.771099	-0.348914
H	-2.005595	1.922466	0.262801
H	-3.014465	2.405517	-1.118156
H	-2.885277	3.450099	0.307913
Mg	-1.103372	-0.646119	0.119936
N	-1.343850	-1.779875	1.847601
N	-2.966724	-1.188602	-0.559359
C	-2.291903	-3.619794	3.194406
H	-1.832348	-4.597452	3.014085
H	-3.338762	-3.790160	3.456538
H	-1.780464	-3.153932	4.038085
C	-2.198217	-2.774423	1.943099
C	-3.084512	-3.132638	0.889022
H	-3.619917	-4.063967	1.038904
C	-3.481928	-2.379275	-0.221252
C	-4.595327	-2.956455	-1.069917
H	-5.534509	-2.423071	-0.902312
H	-4.756046	-4.010961	-0.841076
H	-4.356480	-2.851844	-2.132553
C	-0.459357	-1.388588	2.897868
C	0.690362	-2.138997	3.210600
C	1.587138	-1.625960	4.150805
H	2.481172	-2.193083	4.394435

C	1.377049	-0.397740	4.757952
H	2.095262	-0.010390	5.474011
C	0.247136	0.341433	4.430884
H	0.091805	1.307331	4.899916
C	-0.680733	-0.133329	3.505226
C	1.026281	-3.456389	2.531138
H	0.215479	-3.704081	1.840230
C	1.148409	-4.610409	3.536764
H	2.003612	-4.462438	4.204945
H	1.301475	-5.558367	3.009838
H	0.254154	-4.702790	4.159823
C	2.311423	-3.326626	1.704386
H	2.257161	-2.484724	1.009685
H	2.492935	-4.236622	1.124888
H	3.180578	-3.158841	2.349630
C	-1.941463	0.664174	3.213073
C	-3.708893	-0.324492	-1.417841
C	-3.247537	-0.037665	-2.719186
C	-3.920394	0.921463	-3.481268
H	-3.562335	1.153366	-4.480700
C	-5.044211	1.573713	-2.996277
H	-5.556436	2.312777	-3.604433
C	-5.506935	1.268145	-1.722559
H	-6.383065	1.784270	-1.339669
C	-4.854634	0.337526	-0.914073
C	-2.064883	-0.761090	-3.344797
H	-1.695442	-1.486578	-2.613301
C	-0.915103	0.196300	-3.684258
H	-1.231323	0.966206	-4.396423
H	-0.078319	-0.349172	-4.134701
H	-0.538528	0.697891	-2.787214
C	-2.506649	-1.548748	-4.587362
H	-3.286086	-2.276310	-4.339849
H	-1.659945	-2.086014	-5.025306
H	-2.907009	-0.882088	-5.358374
C	-5.373596	0.119920	0.502618
H	-4.800488	-0.687343	0.965341
C	-6.855192	-0.284094	0.527704
H	-7.500160	0.534482	0.190191
H	-7.157693	-0.542680	1.547612
H	-7.055680	-1.146901	-0.114069
C	-5.158350	1.376586	1.357100
H	-4.097089	1.623442	1.433763
H	-5.549731	1.222925	2.367944
H	-5.672556	2.241780	0.924281
C	-1.728605	2.179227	3.250590
H	-1.555796	2.542918	4.269019
H	-0.877048	2.480014	2.632669
H	-2.283386	0.407180	2.202478

C	-3.062322	0.228606	4.168606
H	-3.285940	-0.836689	4.051390
H	-3.978849	0.794992	3.978282
H	-2.761994	0.399883	5.208259
H	-2.618631	2.693233	2.872994

	147		
LCa-Al(H)L			
Al	-2.007943	0.011351	0.468112
H	-2.994245	0.635014	1.595586
N	-3.029354	0.416429	-1.200178
N	-2.555943	-1.899190	0.396774
C	-4.233475	-0.027722	-3.313012
H	-4.249369	1.054889	-3.436531
H	-5.261471	-0.400012	-3.333565
H	-3.711657	-0.473013	-4.166675
C	-3.556333	-0.464760	-2.032164
C	-3.535251	-1.855053	-1.800914
H	-3.960428	-2.471838	-2.584026
C	-3.120553	-2.516566	-0.635943
C	-3.377614	-4.003722	-0.564578
H	-2.511203	-4.539316	-0.170313
H	-3.629259	-4.401487	-1.548655
H	-4.210729	-4.210613	0.113221
C	-3.182054	1.822270	-1.414929
C	-4.286662	2.479844	-0.839213
C	-4.411514	3.856801	-1.031407
H	-5.264532	4.378534	-0.607070
C	-3.468525	4.572370	-1.759044
H	-3.586474	5.642211	-1.901972
C	-2.369307	3.913624	-2.294339
H	-1.628721	4.479173	-2.853572
C	-2.198294	2.538660	-2.122966
C	-5.350835	1.719668	-0.062153
H	-4.972877	0.709603	0.113915
C	-6.646815	1.608796	-0.878087
H	-7.082149	2.597890	-1.061738
H	-7.387638	1.008661	-0.339024
H	-6.467962	1.137626	-1.849351
C	-5.614412	2.340316	1.313789
H	-4.688721	2.388996	1.893259
H	-6.330721	1.725691	1.869021
H	-6.036334	3.348653	1.235143
C	-0.982173	1.850615	-2.722482
H	-0.965415	0.831377	-2.324233
C	-1.074386	1.757103	-4.250231
H	-1.958357	1.194388	-4.563007
H	-0.188279	1.257766	-4.656201

H	-1.134713	2.754252	-4.700064
C	0.325610	2.536643	-2.308213
H	0.440786	3.520513	-2.774978
H	1.201237	1.945903	-2.604550
H	0.355153	2.705593	-1.226542
C	-2.339281	-2.617894	1.614537
C	-3.387592	-2.705036	2.553941
C	-3.159978	-3.401336	3.741936
H	-3.959731	-3.481533	4.473093
C	-1.930429	-3.989294	4.011043
H	-1.775456	-4.532319	4.938287
C	-0.896087	-3.864832	3.093131
H	0.068470	-4.314905	3.313370
C	-1.073942	-3.172224	1.893728
C	-4.742949	-2.052713	2.316241
H	-4.717257	-1.575495	1.332860
C	-5.885695	-3.078474	2.318224
H	-5.719066	-3.880934	1.593512
H	-6.832298	-2.588456	2.067604
H	-6.003245	-3.541958	3.304105
C	-5.012848	-0.949832	3.349196
H	-5.064242	-1.360159	4.364363
H	-5.970446	-0.461944	3.136603
H	-4.228284	-0.190289	3.313685
C	0.097772	-3.036658	0.932336
H	-0.230800	-2.374319	0.123398
C	0.512621	-4.367125	0.294236
H	0.825804	-5.089809	1.056138
H	1.347986	-4.213216	-0.395768
H	-0.312546	-4.809930	-0.270442
C	1.305714	-2.386349	1.626060
H	1.011640	-1.479044	2.172072
H	2.101337	-2.166870	0.899334
H	1.755951	-3.051983	2.369246
Ca	1.072474	0.315723	0.074743
N	2.709669	1.967515	0.607787
N	2.886171	-0.308096	-1.391286
C	4.385591	3.711423	0.165641
H	3.779705	4.597619	-0.052261
H	5.317869	3.790228	-0.395375
H	4.608373	3.730120	1.235488
C	3.619524	2.458184	-0.210260
C	3.942700	1.882955	-1.466460
H	4.616995	2.476911	-2.072854
C	3.672055	0.597824	-1.973833
C	4.329318	0.260378	-3.300519
H	4.745164	-0.749827	-3.304049
H	5.118139	0.970131	-3.553250
H	3.571085	0.293736	-4.091515

C	2.512074	2.487917	1.916339
C	1.729057	3.637999	2.144058
C	1.511802	4.046855	3.461909
H	0.909190	4.930582	3.651703
C	2.034403	3.338775	4.534879
H	1.853781	3.674597	5.551165
C	2.767232	2.180341	4.301876
H	3.147918	1.615727	5.146838
C	3.005239	1.729412	3.003869
C	1.063989	4.397820	1.006589
H	1.476653	4.020973	0.066340
C	1.347314	5.904957	1.055390
H	0.873390	6.375676	1.923191
H	0.947292	6.392486	0.160296
H	2.420849	6.111370	1.110373
C	-0.449628	4.130735	0.993606
H	-0.675875	3.059062	0.941866
H	-0.926235	4.612759	0.133639
H	-0.919437	4.516142	1.905036
C	3.794595	0.453004	2.749772
C	2.967526	-1.667031	-1.805928
C	2.092488	-2.198392	-2.774451
C	2.188953	-3.554515	-3.100685
H	1.516525	-3.972524	-3.844781
C	3.129617	-4.377556	-2.499931
H	3.195155	-5.425914	-2.773627
C	3.976031	-3.851659	-1.529693
H	4.698847	-4.503505	-1.048961
C	3.903375	-2.510212	-1.156511
C	1.054996	-1.343104	-3.485346
H	1.222681	-0.300845	-3.190964
C	-0.367766	-1.731621	-3.063734
H	-0.570543	-2.787589	-3.272346
H	-1.112098	-1.135846	-3.601622
H	-0.539740	-1.572722	-1.992658
C	1.204153	-1.417699	-5.011543
H	2.222357	-1.170755	-5.326717
H	0.513035	-0.722515	-5.498699
H	0.974771	-2.420855	-5.385826
C	4.828962	-1.959439	-0.079992
H	4.336470	-1.075663	0.337728
C	6.168665	-1.488150	-0.665080
H	6.678427	-2.313351	-1.175552
H	6.824350	-1.122414	0.132477
H	6.032679	-0.673450	-1.379756
C	5.076073	-2.951941	1.062083
H	4.139553	-3.354737	1.459727
H	5.608891	-2.454915	1.879141
H	5.697745	-3.795525	0.743495

C	3.663099	-0.588047	3.865068
H	4.204242	-0.288737	4.769152
H	2.616592	-0.758267	4.137015
H	3.391787	0.001022	1.834889
C	5.271137	0.758403	2.460439
H	5.381816	1.382940	1.570085
H	5.830439	-0.167729	2.290219
H	5.725714	1.281610	3.308907
H	4.087964	-1.540354	3.532897

	147		
LCa(μ-H)AlL			
Al	1.748269	-0.086691	-0.247644
N	2.850328	0.561194	1.259622
N	2.776809	-1.765962	-0.346026
C	3.595409	-1.632494	1.909187
H	4.060657	-2.196784	2.707187
C	3.444419	-0.252695	2.127716
C	2.859483	1.969011	1.529278
C	3.369419	-2.320505	0.707934
C	2.776161	-2.483074	-1.585838
C	3.793321	2.779699	0.854197
C	1.931845	2.523561	2.429090
C	4.023808	0.302087	3.408187
H	4.654823	1.172701	3.209659
H	4.612489	-0.454374	3.927913
H	3.222417	0.637057	4.072788
C	3.869714	-3.742321	0.617677
H	3.057703	-4.418035	0.333540
H	4.289200	-4.070091	1.569304
H	4.638462	-3.831676	-0.155576
C	4.694476	2.189283	-0.222366
H	4.103821	1.421295	-0.736767
C	3.849677	-2.301699	-2.475972
C	1.694685	-3.321245	-1.911273
C	2.998339	4.680298	2.139381
H	3.066704	5.734450	2.390053
C	3.852052	4.133990	1.186935
H	4.569047	4.778984	0.690440
C	2.031106	3.882413	2.734878
H	1.328403	4.326642	3.434484
C	4.993580	-1.350250	-2.159024
H	4.847700	-0.975672	-1.141835
C	5.932347	1.497235	0.365383
H	6.542107	2.209166	0.933572
H	6.551822	1.085746	-0.439665
H	5.660544	0.670419	1.026593
C	1.720322	-3.998513	-3.131346

H	0.893533	-4.650986	-3.398985
C	4.963107	-0.138822	-3.100046
H	5.099097	-0.444812	-4.143378
H	5.769660	0.557678	-2.847031
H	4.009066	0.392521	-3.023573
C	5.106747	3.215479	-1.281463
H	4.237296	3.747704	-1.678730
H	5.602549	2.709400	-2.114667
H	5.813800	3.953105	-0.885418
C	3.832691	-2.996053	-3.686744
H	4.652328	-2.866908	-4.388404
C	0.762786	1.710816	2.965732
H	0.935277	0.659564	2.719122
C	2.782490	-3.845007	-4.014201
H	2.786947	-4.378304	-4.959856
C	0.486778	-3.442802	-0.995038
H	0.723568	-2.944261	-0.050846
C	-0.507144	2.151906	2.224610
H	-0.799804	3.169965	2.504078
H	-1.361740	1.499304	2.436467
H	-0.313692	2.189332	1.146282
C	-0.701964	-2.692533	-1.615189
H	-0.370901	-1.700134	-1.955973
H	-1.529852	-2.627065	-0.895928
H	-1.085521	-3.203854	-2.504930
C	6.359951	-2.045905	-2.200132
H	6.389986	-2.910043	-1.529063
H	7.147149	-1.350050	-1.891126
H	6.604379	-2.396643	-3.208792
C	0.099261	-4.889049	-0.671440
H	-0.220683	-5.434816	-1.566095
H	-0.729279	-4.900820	0.043804
H	0.939361	-5.434370	-0.230412
C	0.560058	1.815410	4.479933
H	1.452735	1.508281	5.033161
H	-0.267947	1.168679	4.789185
H	0.311344	2.836422	4.788151
Ca	-1.201143	0.142506	-0.245007
H	0.535101	-0.799665	0.844538
N	-2.927354	-0.606043	1.265614
N	-2.797338	1.882423	-0.550163
C	-4.142566	1.496730	1.429811
H	-4.908787	1.970258	2.033542
C	-3.835678	0.175816	1.831239
C	-2.856064	-1.991109	1.593531
C	-3.771276	2.226509	0.280052
C	-2.643937	2.574273	-1.787780
C	-3.547226	-2.904377	0.758038
C	-2.047682	-2.466251	2.648980

C	-4.662174	-0.317751	3.008255
H	-4.626161	-1.402037	3.118948
H	-5.704837	-0.008218	2.906533
H	-4.275498	0.134465	3.927626
C	-4.595959	3.462755	-0.019588
H	-3.960817	4.350958	-0.078360
H	-5.362322	3.627545	0.738327
H	-5.083474	3.359813	-0.994616
C	-4.375640	-2.403225	-0.419730
H	-3.863327	-1.517458	-0.817069
C	-3.214867	2.000889	-2.945034
C	-1.854597	3.739755	-1.887162
C	-2.693479	-4.742505	2.091212
H	-2.636647	-5.808137	2.290921
C	-3.460131	-4.268993	1.032523
H	-3.989108	-4.980014	0.406853
C	-1.988843	-3.844102	2.877885
H	-1.380455	-4.219558	3.695789
C	-3.979808	0.687619	-2.869180
H	-4.142907	0.460554	-1.811519
C	-5.776619	-1.944946	0.012022
H	-6.324789	-2.775624	0.470502
H	-6.346282	-1.596181	-0.856431
H	-5.732327	-1.124752	0.729896
C	-1.678958	4.322978	-3.143300
H	-1.068613	5.217397	-3.232284
C	-3.135859	-0.444423	-3.474684
H	-2.975872	-0.282859	-4.546009
H	-3.617818	-1.417724	-3.343302
H	-2.136318	-0.498230	-3.024276
C	-4.496529	-3.420065	-1.559343
H	-3.519708	-3.798152	-1.876610
H	-4.980700	-2.952186	-2.422523
H	-5.117732	-4.276172	-1.275642
C	-3.014014	2.619441	-4.179308
H	-3.448727	2.183531	-5.074941
C	-1.275748	-1.540099	3.578971
H	-1.351424	-0.526665	3.173454
C	-2.256602	3.779272	-4.283537
H	-2.105908	4.249462	-5.250296
C	-1.157152	4.340865	-0.676674
H	-1.542465	3.830396	0.210131
C	0.213481	-1.902022	3.660201
H	0.361338	-2.890303	4.109563
H	0.741745	-1.177220	4.290685
H	0.678126	-1.891687	2.671649
C	0.358152	4.094329	-0.744235
H	0.597620	3.033063	-0.878199
H	0.851489	4.437309	0.171709

H	0.798570	4.634008	-1.590704
C	-5.360577	0.750398	-3.530529
H	-5.964712	1.554123	-3.099433
H	-5.894105	-0.194823	-3.381985
H	-5.289246	0.923726	-4.609662
C	-1.449257	5.837523	-0.504167
H	-1.013781	6.428988	-1.316671
H	-1.012738	6.198193	0.433139
H	-2.524232	6.043436	-0.483118
C	-1.887378	-1.539275	4.989256
H	-2.952332	-1.297515	4.975605
H	-1.376637	-0.810442	5.628174
H	-1.777114	-2.524117	5.456882

	147		
LZn-<br>Al(H)L			
Al	1.246771	0.410050	-0.287631
H	2.198064	0.337033	1.000127
N	2.283985	-0.415279	-1.723260
N	1.422915	2.210697	-1.034731
C	3.163530	-0.665237	-4.002661
H	4.150437	-1.027719	-3.703283
H	3.264879	-0.078762	-4.916181
H	2.556533	-1.550170	-4.213669
C	2.524528	0.151383	-2.905529
C	2.253659	1.496452	-3.182097
H	2.500537	1.843867	-4.177064
C	1.848472	2.482403	-2.263655
C	1.917022	3.912715	-2.741538
H	0.923346	4.253020	-3.045061
H	2.583424	3.997629	-3.600399
H	2.259503	4.583605	-1.951094
C	2.961894	-1.646699	-1.427528
C	4.275128	-1.570241	-0.919793
C	4.963489	-2.758581	-0.676759
H	5.979370	-2.717608	-0.293570
C	4.373386	-3.993759	-0.914848
H	4.926571	-4.908682	-0.725856
C	3.064168	-4.050869	-1.370260
H	2.595043	-5.018812	-1.524009
C	2.332875	-2.887620	-1.621861
C	4.952914	-0.239040	-0.628121
H	4.235806	0.557341	-0.839550
C	6.174075	-0.008351	-1.528547
H	6.944671	-0.768600	-1.358805
H	6.617403	0.971075	-1.320261
H	5.902984	-0.035103	-2.588438
C	5.329895	-0.124247	0.853875

H	4.443139	-0.231050	1.485134
H	5.775290	0.857176	1.050464
H	6.061916	-0.886198	1.144168
C	0.882408	-2.997914	-2.056102
H	0.510953	-1.982154	-2.217141
C	0.713216	-3.776533	-3.365731
H	1.249360	-3.303827	-4.194402
H	-0.346097	-3.831740	-3.634531
H	1.083667	-4.802903	-3.271894
C	0.036537	-3.635878	-0.945862
H	0.342483	-4.671194	-0.758374
H	-1.023447	-3.643827	-1.218767
H	0.137355	-3.088679	-0.003233
C	1.040717	3.296049	-0.172726
C	1.933366	3.723598	0.829415
C	1.546885	4.786086	1.649858
H	2.227506	5.137985	2.419299
C	0.316517	5.409481	1.493317
H	0.036410	6.235668	2.139557
C	-0.558282	4.962557	0.512606
H	-1.528639	5.439161	0.406825
C	-0.222315	3.901494	-0.329525
C	3.314822	3.110416	1.004409
H	3.357464	2.200845	0.402924
C	4.405570	4.058065	0.486813
H	4.248870	4.307592	-0.567786
H	5.391036	3.588380	0.579565
H	4.422263	4.994516	1.055941
C	3.571956	2.693471	2.456135
H	3.568298	3.552518	3.136291
H	4.550422	2.211103	2.541719
H	2.813046	1.980039	2.789274
C	-1.241930	3.420300	-1.350058
H	-0.773442	2.657637	-1.978350
C	-1.749693	4.544041	-2.265968
H	-2.355695	5.266752	-1.708974
H	-2.382903	4.123027	-3.053457
H	-0.934055	5.098296	-2.739169
C	-2.432194	2.764458	-0.646067
H	-2.108433	1.939149	-0.004313
H	-3.142394	2.371380	-1.377173
H	-2.955848	3.493385	-0.017028
Zn	-0.963274	-0.594527	0.153193
N	-1.120337	-1.531099	1.992297
N	-2.748385	-1.266881	-0.407611
C	-2.023968	-3.189011	3.584797
H	-1.555852	-4.177892	3.533819
H	-3.068200	-3.332387	3.872966
H	-1.514036	-2.609541	4.355493

C	-1.947780	-2.521871	2.229160
C	-2.819126	-3.044848	1.234032
H	-3.317337	-3.972775	1.492030
C	-3.222913	-2.438645	0.046990
C	-4.238410	-3.179722	-0.798510
H	-5.049962	-2.533151	-1.137282
H	-4.660804	-4.031408	-0.263198
H	-3.734281	-3.550202	-1.699610
C	-0.249984	-0.985656	2.980997
C	0.916384	-1.663776	3.379065
C	1.797311	-1.018366	4.250216
H	2.706442	-1.527182	4.558540
C	1.547972	0.265011	4.711106
H	2.252854	0.754521	5.376187
C	0.394910	0.926724	4.305312
H	0.208505	1.934203	4.662330
C	-0.516279	0.321118	3.441972
C	1.278134	-3.048319	2.866822
H	0.466831	-3.400461	2.223838
C	1.439947	-4.061358	4.009255
H	2.301975	-3.813527	4.638011
H	1.604528	-5.065891	3.604837
H	0.557226	-4.090007	4.654678
C	2.546912	-2.996002	2.009250
H	2.448414	-2.267416	1.201769
H	2.754765	-3.973494	1.564108
H	3.417248	-2.704870	2.607822
C	-1.810213	1.026503	3.068834
C	-3.567157	-0.503951	-1.290571
C	-3.256166	-0.389806	-2.657559
C	-4.059639	0.419056	-3.465674
H	-3.820293	0.524311	-4.520444
C	-5.173004	1.070466	-2.953914
H	-5.799120	1.676825	-3.601190
C	-5.475378	0.945262	-1.602875
H	-6.341446	1.466066	-1.205361
C	-4.672181	0.192687	-0.746420
C	-2.087923	-1.134727	-3.280368
H	-1.679098	-1.796640	-2.511655
C	-0.975290	-0.171143	-3.711423
H	-1.333240	0.533987	-4.470171
H	-0.127989	-0.720811	-4.137202
H	-0.605341	0.406828	-2.859428
C	-2.541002	-2.013421	-4.454148
H	-3.311139	-2.725674	-4.141043
H	-1.694640	-2.575931	-4.860673
H	-2.955240	-1.412941	-5.270872
C	-4.994792	0.154208	0.744447
H	-4.120569	-0.251438	1.262477

C	-6.185763	-0.765539	1.052168
H	-7.086265	-0.423463	0.528869
H	-6.396689	-0.764880	2.126884
H	-5.990997	-1.797835	0.752673
C	-5.255817	1.554380	1.314360
H	-4.396076	2.211067	1.161401
H	-5.450644	1.490002	2.389122
H	-6.130369	2.028652	0.857413
C	-1.679681	2.548125	2.992926
H	-1.546164	3.000237	3.981774
H	-0.834777	2.843778	2.364103
H	-2.111418	0.681841	2.072462
C	-2.923778	0.604827	4.038712
H	-3.100679	-0.474483	3.992031
H	-3.861281	1.114897	3.799641
H	-2.647985	0.859190	5.068169
H	-2.588659	2.978581	2.560828

	74		
LMgH			
Mg	0.000000	0.000012	-0.939902
N	-1.467549	0.000002	0.471549
N	1.467549	0.000008	0.471549
C	-2.470600	-0.000065	2.706309
C	-1.270382	-0.000020	1.786464
C	0.000000	-0.000007	2.392986
C	1.270382	0.000013	1.786464
C	2.470600	0.000028	2.706309
C	-2.801719	-0.000011	-0.042288
C	-3.427907	-1.226184	-0.334450
C	-4.691483	-1.202510	-0.926425
C	-5.322462	-0.000030	-1.221896
C	-4.691537	1.202461	-0.926347
C	-3.427961	1.226156	-0.334377
C	-2.722520	-2.545674	-0.058785
C	-3.640290	-3.597080	0.575141
C	-2.077358	-3.086156	-1.344435
C	-2.722617	2.545655	-0.058643
C	-3.640447	3.597054	0.575205
C	-2.077355	3.086152	-1.344237
C	2.801719	0.000015	-0.042288
C	3.427918	1.226186	-0.334442
C	4.691493	1.202505	-0.926417
C	5.322461	0.000022	-1.221896
C	4.691527	-1.202466	-0.926356
C	3.427951	-1.226153	-0.334385
C	2.722538	2.545681	-0.058773
C	2.077362	3.086159	-1.344417
C	3.640318	3.597087	0.575135

C	2.722599	-2.545649	-0.058654
C	3.640419	-3.597045	0.575212
C	2.077352	-3.086149	-1.344254
H	-1.917022	2.348389	0.655087
H	-5.189712	2.137073	-1.166412
H	1.916996	-2.348377	0.655065
H	5.189693	-2.137081	-1.166426
H	-1.916869	-2.348401	0.654880
H	6.303916	0.000024	-1.685787
H	0.000000	-0.000016	3.475783
H	5.189631	2.137123	-1.166537
H	-5.189613	-2.137131	-1.166551
H	1.916895	2.348413	0.654902
H	3.058828	-4.478880	0.862567
H	3.096650	0.878022	2.517584
H	-6.303916	-0.000037	-1.685786
H	-3.058679	-4.478923	0.862430
H	1.406511	-2.352440	-1.805963
H	3.096652	-0.877971	2.517612
H	2.168971	0.000044	3.753960
H	-3.096637	-0.878066	2.517571
H	-1.501869	-3.995131	-1.139016
H	-3.058859	4.478888	0.862570
H	-2.168971	-0.000091	3.753959
H	4.134046	-3.206250	1.470588
H	2.844770	-3.327196	-2.088056
H	4.418550	3.932416	-0.118501
H	-3.096666	0.877927	2.517624
H	-1.406549	-2.352436	-1.806173
H	1.501879	-3.995123	-1.138787
H	-1.406512	2.352440	-1.805939
H	-4.418536	-3.932406	-0.118480
H	-4.418616	3.932402	-0.118492
H	4.418602	-3.932392	-0.118469
H	-4.133838	-3.206298	1.470566
H	1.406551	2.352438	-1.806150
H	4.133883	3.206307	1.470552
H	-1.501881	3.995124	-1.138765
H	-2.844765	3.327199	-2.088047
H	3.058711	4.478930	0.862433
H	2.844820	3.327201	-2.088179
H	1.501872	3.995133	-1.138995
H	0.000000	0.000032	-2.658218
H	-2.844824	-3.327197	-2.088189
H	-4.134092	3.206261	1.470572

	74		
LCaH			
Ca	0.000083	-0.180917	-1.336253

N	1.478646	-0.110086	0.538967
N	-1.478375	-0.111396	0.539017
C	2.467962	-0.093603	2.779438
C	1.273867	-0.129807	1.848204
C	0.000228	-0.169769	2.451066
C	-1.273471	-0.131673	1.848245
C	-2.467568	-0.097837	2.779566
C	2.799336	-0.000446	0.021058
C	3.315836	1.276246	-0.289089
C	4.556955	1.363836	-0.921116
C	5.280590	0.222773	-1.245529
C	4.761725	-1.029557	-0.939862
C	3.524923	-1.165091	-0.307801
C	2.525241	2.538419	0.024878
C	3.341595	3.556671	0.830672
C	1.980499	3.178441	-1.261193
C	2.960288	-2.547109	-0.015349
C	3.947636	-3.435584	0.751778
C	2.504496	-3.236965	-1.310472
C	-2.799205	-0.003539	0.021094
C	-3.521240	-1.169098	-0.312225
C	-4.758028	-1.034960	-0.944681
C	-5.280245	0.216882	-1.246424
C	-4.560214	1.358924	-0.917321
C	-3.319364	1.272764	-0.284627
C	-2.953358	-2.550577	-0.023463
C	-2.498886	-3.237623	-1.320543
C	-3.937726	-3.441943	0.744188
C	-2.532794	2.536062	0.034875
C	-3.354186	3.550674	0.840155
C	-1.985003	3.180107	-1.247842
H	2.079290	-2.415360	0.619872
H	5.327694	-1.918499	-1.204419
H	-1.677413	2.246930	0.652626
H	-4.969326	2.335071	-1.163306
H	1.668520	2.248622	0.640512
H	-6.241615	0.301983	-1.742969
H	0.000241	-0.194319	3.534067
H	-5.321238	-1.924652	-1.212610
H	4.963324	2.340328	-1.170299
H	-2.071445	-2.418260	0.610388
H	-2.728873	4.401416	1.129858
H	-3.137538	-0.939025	2.574775
H	6.242126	0.308905	-1.741575
H	2.713696	4.407172	1.115464
H	-1.382305	2.480610	-1.841212
H	-3.050903	0.814408	2.617006
H	-2.163309	-0.136929	3.825950
H	3.051205	0.818298	2.614711

H	1.349866	4.043258	-1.030672
H	3.470629	-4.381293	1.028750
H	2.163697	-0.130366	3.825902
H	-3.757451	3.098096	1.751059
H	-2.800002	3.514144	-1.898658
H	-4.820882	-3.681803	0.142624
H	3.138042	-0.935191	2.576629
H	1.382866	2.475953	-1.856196
H	-1.358219	4.046635	-1.013233
H	1.794096	-2.625876	-1.881208
H	4.185581	3.947849	0.252666
H	4.829662	-3.675739	0.148687
H	-4.196494	3.942147	0.259921
H	3.741937	3.107348	1.744461
H	-1.791024	-2.624304	-1.892061
H	-4.281116	-2.954943	1.662012
H	2.026698	-4.198010	-1.093207
H	3.355587	-3.422485	-1.974484
H	-3.458908	-4.387652	1.018019
H	-3.350968	-3.424079	-1.983013
H	-2.018654	-4.197983	-1.105623
H	0.000374	-0.351356	-3.446732
H	2.797072	3.514186	-1.909165
H	4.292600	-2.946230	1.667760

74

LZnH

Zn	0.000000	0.000052	-0.783901
N	1.455281	0.000095	0.532617
N	-1.455281	-0.000077	0.532617
C	2.474179	0.000103	2.757031
C	1.266727	0.000071	1.848404
C	0.000000	-0.000031	2.458648
C	-1.266727	-0.000113	1.848404
C	-2.474179	-0.000211	2.757030
C	2.783608	0.000167	0.003423
C	3.406573	1.226801	-0.288709
C	4.667913	1.202809	-0.885491
C	5.297549	0.000274	-1.183671
C	4.668193	-1.202315	-0.885114
C	3.406847	-1.226416	-0.288351
C	2.704531	2.547164	-0.009475
C	3.622934	3.587124	0.642401
C	2.073190	3.099471	-1.296557
C	2.705119	-2.546851	-0.008667
C	3.623746	-3.586359	0.643609
C	2.073956	-3.099781	-1.295571
C	-2.783608	-0.000163	0.003423
C	-3.406549	-1.226806	-0.288725

C	-4.667890	-1.202830	-0.885504
C	-5.297549	-0.000304	-1.183671
C	-4.668215	1.202293	-0.885102
C	-3.406870	1.226411	-0.288336
C	-2.704483	-2.547158	-0.009501
C	-2.073127	-3.099442	-1.296585
C	-3.622868	-3.587142	0.642361
C	-2.705167	2.546857	-0.008642
C	-3.623812	3.586341	0.643649
C	-2.074020	3.099811	-1.295543
H	1.890704	-2.348519	0.694534
H	5.165388	-2.137077	-1.126778
H	-1.890744	2.348534	0.694554
H	-5.165427	2.137049	-1.126756
H	1.890182	2.348888	0.693816
H	-6.277418	-0.000358	-1.651011
H	0.000000	-0.000056	3.541273
H	-5.164844	-2.137641	-1.127480
H	5.164883	2.137613	-1.127458
H	-1.890140	-2.348873	0.693796
H	-3.045400	4.469873	0.932302
H	-3.098213	-0.878448	2.563798
H	6.277418	0.000315	-1.651011
H	3.044313	4.470627	0.930726
H	-1.391668	2.374340	-1.750660
H	-3.098295	0.877987	2.563872
H	-2.178930	-0.000241	3.806523
H	3.098229	0.878339	2.563841
H	1.511522	4.016915	-1.090083
H	3.045317	-4.469881	0.932260
H	2.178930	0.000087	3.806523
H	-4.106128	3.185276	1.540809
H	-2.848180	3.331556	-2.035521
H	-4.410339	-3.922514	-0.040806
H	3.098279	-0.878096	2.563830
H	1.390988	2.373654	-1.751350
H	-1.512565	4.017311	-1.088744
H	1.391615	-2.374295	-1.750680
H	4.410418	3.922482	-0.040758
H	4.411309	-3.921806	-0.039415
H	-4.411389	3.921774	-0.039365
H	4.105367	3.186524	1.539705
H	-1.390936	-2.373612	-1.751371
H	-4.105316	-3.186558	1.539665
H	1.512484	-4.017273	-1.088777
H	2.848109	-3.331534	-2.035554
H	-3.044230	-4.470634	0.930685
H	-2.847184	-3.331099	-2.036698
H	-1.511443	-4.016877	-1.090116

H	0.000000	0.000089	-2.326851
H	2.847254	3.331119	-2.036666
H	4.106079	-3.185312	1.540767

73

LAl[I]

Al	-0.000002	-0.224876	-0.978596
N	-1.389161	-0.137133	0.451449
N	1.389159	-0.137144	0.451450
C	-0.000002	-0.204400	2.408410
H	-0.000002	-0.231891	3.490488
C	-1.249421	-0.160683	1.777638
C	-2.714730	-0.010850	-0.092265
C	1.249418	-0.160697	1.777638
C	2.714731	-0.010874	-0.092261
C	-3.217721	1.274513	-0.367307
C	-3.453079	-1.166898	-0.404298
C	-2.474853	-0.124989	2.657682
H	-3.053317	0.784734	2.472344
H	-2.201545	-0.161352	3.712738
H	-3.134642	-0.967624	2.432232
C	2.474850	-0.125011	2.657684
H	3.134667	-0.967614	2.432193
H	2.201543	-0.161431	3.712739
H	3.053284	0.784740	2.472390
C	-2.422910	2.534595	-0.053604
H	-1.526942	2.239478	0.500519
C	3.217753	1.274488	-0.367260
C	3.453053	-1.166928	-0.404337
C	-5.218530	0.249672	-1.277963
H	-6.193459	0.350735	-1.744691
C	-4.476113	1.380254	-0.961984
H	-4.879448	2.363272	-1.188299
C	-4.706280	-1.010951	-0.999188
H	-5.289216	-1.891161	-1.255028
C	2.422968	2.534575	-0.053514
H	1.527023	2.239462	0.500649
C	-3.208479	3.507596	0.835908
H	-4.089481	3.903729	0.319823
H	-2.578143	4.358626	1.113695
H	-3.551330	3.023639	1.755935
C	4.706256	-1.010990	-0.999225
H	5.289170	-1.891206	-1.255095
C	1.957148	3.223888	-1.343879
H	2.813062	3.558577	-1.940435
H	1.343165	4.100359	-1.110441
H	1.363595	2.542016	-1.961101
C	-1.957145	3.223900	-1.343993
H	-1.363629	2.542020	-1.961240
H	-1.343140	4.100364	-1.110586
H	-2.813082	3.558600	-1.940510
C	4.476143	1.380218	-0.961938
H	4.879502	2.363235	-1.188222
C	-2.914066	-2.563830	-0.133003
H	-1.995620	-2.461886	0.452765

C	5.218533	0.249630	-1.277958
H	6.193463	0.350685	-1.744686
C	2.914011	-2.563857	-0.133082
H	1.995547	-2.461910	0.452658
C	-2.545971	-3.271173	-1.445399
H	-3.432880	-3.414192	-2.072845
H	-2.112176	-4.256149	-1.241825
H	-1.819872	-2.685235	-2.017505
C	2.545952	-3.271175	-1.445501
H	1.819883	-2.685218	-2.017625
H	2.112132	-4.256147	-1.241957
H	3.432880	-3.414203	-2.072917
C	3.208582	3.507568	0.835965
H	3.551489	3.023601	1.755967
H	2.578258	4.358592	1.113797
H	4.089553	3.903713	0.319835
C	3.890284	-3.414400	0.690959
H	4.806265	-3.632509	0.131728
H	3.428462	-4.372118	0.951902
H	4.179988	-2.911801	1.619314
C	-3.890376	-3.414349	0.691019
H	-4.180120	-2.911723	1.619347
H	-3.428567	-4.372061	0.952007
H	-4.806332	-3.632473	0.131755

12

C_6H_6

C	-1.376989	0.218157	-0.000041
H	-2.449334	0.388097	-0.000273
C	-0.877436	-1.083260	0.000065
H	-1.560282	-1.927396	0.000017
C	0.499580	-1.301433	0.000040
H	0.888148	-2.315261	-0.000071
C	0.877382	1.083291	0.000034
H	1.560573	1.927140	0.000114
C	-0.499526	1.301441	0.000009
H	-0.888513	2.315098	0.000041
C	1.376993	-0.218158	-0.000026
H	2.449379	-0.387900	-0.000317

3

CaH_2

Ca	0.000001	0.000128	-0.000002
H	1.098489	-0.000455	-1.793997
H	-1.098516	-0.002107	1.794040

76

A1

Al	-0.017718	-0.680204	-0.538266
N	-1.410936	0.108112	0.597648
N	1.408868	-0.003801	0.629924
C	-0.014512	0.215586	2.554406
H	-0.022022	0.345285	3.628862
C	-1.259392	0.287348	1.908835
C	-2.707654	0.336990	0.026677

C	1.244884	0.188952	1.937303
C	2.710131	0.213524	0.066421
C	-2.963503	1.570046	-0.604825
C	-3.685756	-0.671768	0.080958
C	-2.459226	0.614009	2.766621
H	-3.033416	1.441160	2.340880
H	-2.153271	0.876505	3.779609
H	-3.133582	-0.245679	2.817454
C	2.450265	0.442444	2.809945
H	3.191139	-0.352244	2.688259
H	2.164853	0.506587	3.860248
H	2.941813	1.376484	2.522665
C	-1.855531	2.601789	-0.778442
H	-0.932929	2.039108	-0.966803
C	3.057972	1.507799	-0.361051
C	3.601848	-0.863701	-0.080535
C	-5.249560	0.841251	-0.982400
H	-6.245491	1.047074	-1.362175
C	-4.251916	1.804126	-1.088501
H	-4.482942	2.748551	-1.568875
C	-4.958504	-0.394469	-0.421532
H	-5.729585	-1.158584	-0.383050
C	2.085714	2.673374	-0.259459
H	1.183310	2.318121	0.246230
C	-1.628390	3.444292	0.484994
H	-2.540388	3.987204	0.758314
H	-0.835880	4.180335	0.308390
H	-1.322566	2.831342	1.336405
C	4.857654	-0.619643	-0.638867
H	5.559313	-1.440643	-0.759107
C	1.670035	3.152670	-1.656229
H	2.533905	3.515651	-2.223811
H	0.953606	3.976596	-1.575163
H	1.204574	2.343383	-2.227687
C	-2.078020	3.513611	-1.988861
H	-2.283312	2.934335	-2.893784
H	-1.183514	4.115851	-2.169709
H	-2.907530	4.211311	-1.828570
C	4.323891	1.706096	-0.914628
H	4.610000	2.698881	-1.250986
C	-3.350784	-2.070342	0.577066
H	-2.378919	-2.033513	1.076969
C	5.223458	0.655985	-1.049780
H	6.203924	0.829705	-1.482059
C	3.206098	-2.278759	0.311103
H	2.228535	-2.232673	0.799554
C	-3.197699	-3.003655	-0.635579
H	-4.165086	-3.220197	-1.100645
H	-2.744058	-3.959972	-0.346800
H	-2.598582	-2.499819	-1.408166
C	3.051064	-3.145270	-0.948789
H	2.477885	-2.596030	-1.710051
H	2.577350	-4.107695	-0.714028
H	4.019316	-3.367630	-1.409269
C	2.650655	3.832942	0.571130

H	2.947888	3.504274	1.571720
H	1.896129	4.618475	0.684320
H	3.527802	4.281470	0.092240
C	4.188711	-2.928715	1.292467
H	5.183286	-3.048427	0.849876
H	3.832641	-3.921955	1.584488
H	4.298510	-2.329254	2.200931
C	-4.371851	-2.641912	1.566713
H	-4.513281	-1.979058	2.425523
H	-4.031219	-3.612454	1.941077
H	-5.350832	-2.794383	1.100460
Ca	-0.109115	-3.303437	-1.879178
H	-0.085677	-2.327164	0.225752
H	-0.175065	-5.226670	-2.742457

76
A2

Al	-0.002036	0.565184	-0.737666
H	-0.075309	-0.575166	-1.884146
N	1.417459	-0.070580	0.492060
N	-1.428653	0.037764	0.523902
C	2.450725	-0.315166	2.712890
H	3.122816	-1.075730	2.309861
H	2.129482	-0.614339	3.711051
H	3.027283	0.610062	2.802658
C	1.259351	-0.091102	1.810065
C	0.012140	0.073474	2.444919
H	0.025856	0.067937	3.527329
C	-1.255133	0.007322	1.839116
C	-2.452781	-0.131563	2.748033
H	-3.101892	0.743443	2.651313
H	-2.146803	-0.226834	3.790034
H	-3.055341	-1.001078	2.473836
C	2.711375	-0.341226	-0.063423
C	2.974286	-1.633681	-0.566285
C	4.248156	-1.897527	-1.071162
H	4.479712	-2.885725	-1.452667
C	5.230731	-0.914581	-1.109421
H	6.214959	-1.143565	-1.506089
C	4.939774	0.364059	-0.659434
H	5.702378	1.135834	-0.717519
C	3.682328	0.676410	-0.136323
C	1.896403	-2.710716	-0.561797
H	0.949735	-2.202272	-0.768548
C	1.767394	-3.405150	0.801628
H	2.709847	-3.887088	1.086718
H	0.991776	-4.178018	0.755190
H	1.484292	-2.705983	1.592406
C	2.094838	-3.752203	-1.667345
H	2.224759	-3.277185	-2.644250
H	1.218282	-4.402938	-1.724474
H	2.960396	-4.396916	-1.476848
C	3.402807	2.109651	0.295722
H	2.407993	2.131382	0.754884
C	4.413346	2.638703	1.323424

H	4.498288	1.976788	2.189152
H	4.111883	3.629331	1.679114
H	5.412507	2.736022	0.886360
C	3.377667	3.037942	-0.929784
H	4.368769	3.116773	-1.388429
H	3.076830	4.057591	-0.648998
H	2.705331	2.641040	-1.701101
C	-2.712920	-0.271161	-0.027971
C	-2.992450	-1.611870	-0.358931
C	-4.234758	-1.914074	-0.916766
H	-4.470157	-2.944520	-1.168073
C	-5.176671	-0.921668	-1.159447
H	-6.137481	-1.175537	-1.596298
C	-4.880128	0.397306	-0.844341
H	-5.618238	1.170111	-1.041531
C	-3.652512	0.746857	-0.276586
C	-1.986972	-2.724946	-0.104699
H	-1.065689	-2.265693	0.262642
C	-2.482780	-3.696178	0.976002
H	-2.693856	-3.179156	1.916976
H	-1.723453	-4.459680	1.175772
H	-3.399168	-4.207868	0.661108
C	-1.638584	-3.474953	-1.394972
H	-2.518782	-3.957121	-1.834124
H	-0.906189	-4.260367	-1.182111
H	-1.209312	-2.792651	-2.134125
C	-3.371780	2.207079	0.047880
H	-2.392011	2.248194	0.538878
C	-4.406752	2.809504	1.008448
H	-5.398441	2.856416	0.546612
H	-4.122443	3.829722	1.286445
H	-4.498582	2.219804	1.924658
C	-3.296306	3.046671	-1.236859
H	-2.587736	2.604511	-1.948776
H	-3.011622	4.085329	-1.014965
H	-4.266660	3.088495	-1.742294
Ca	0.054979	3.703887	-1.139192
H	0.056472	5.827954	-1.221361

	88		
A3			
Al	-0.370606	-0.369673	-0.915029
N	-2.016989	-0.872636	0.063884
N	0.684666	-1.677570	0.140932
C	-1.218081	-3.069175	0.613138
H	-1.525290	-4.057258	0.931748
C	-2.239080	-2.103663	0.500713
C	-3.075822	0.090500	0.062810
C	0.162758	-2.836893	0.539281
C	2.078439	-1.442959	0.338792
C	-3.116545	1.057465	1.091241
C	-4.006579	0.107239	-0.991996
C	-3.629469	-2.518500	0.921067
H	-4.108874	-1.743633	1.524318
H	-3.605806	-3.450546	1.487016

H	-4.255003	-2.666854	0.035358
C	1.080485	-3.965459	0.949724
H	1.652374	-4.309833	0.081610
H	0.514699	-4.806932	1.350670
H	1.808868	-3.639210	1.696335
C	-2.051188	1.083027	2.181581
H	-1.100881	0.835827	1.685405
C	2.495615	-0.823492	1.533615
C	3.008736	-1.796410	-0.656526
C	-5.097742	2.003449	0.055965
H	-5.887870	2.747854	0.056767
C	-4.145010	2.000570	1.069118
H	-4.198980	2.758176	1.842621
C	-5.017171	1.071864	-0.968359
H	-5.745127	1.101386	-1.774056
C	1.494636	-0.441040	2.613700
H	0.494218	-0.581937	2.197094
C	-2.279519	0.012549	3.258773
H	-3.252393	0.154314	3.742809
H	-1.504193	0.085027	4.030196
H	-2.240151	-0.998659	2.848037
C	4.363896	-1.553769	-0.417938
H	5.096195	-1.824389	-1.173310
C	1.618362	1.029232	3.024825
H	2.598453	1.256437	3.458588
H	0.860408	1.278798	3.773058
H	1.472120	1.693772	2.165592
C	-1.891859	2.458999	2.838585
H	-1.758871	3.262133	2.104625
H	-1.017208	2.457133	3.495996
H	-2.756366	2.708884	3.463717
C	3.859749	-0.598946	1.728983
H	4.200660	-0.142211	2.654775
C	-3.902332	-0.842401	-2.176947
H	-3.159985	-1.609677	-1.940391
C	4.793375	-0.964764	0.765871
H	5.851257	-0.789151	0.935728
C	2.560265	-2.382384	-1.985886
H	1.508604	-2.667974	-1.891494
C	-3.383165	-0.092945	-3.412921
H	-4.078723	0.700929	-3.709311
H	-3.267236	-0.782758	-4.255346
H	-2.406097	0.356525	-3.213671
C	2.630139	-1.310914	-3.084004
H	1.994505	-0.456142	-2.830638
H	2.275143	-1.715206	-4.037079
H	3.660135	-0.957153	-3.220125
C	1.615849	-1.355151	3.840753
H	1.460853	-2.403550	3.571255
H	0.864472	-1.088174	4.591322
H	2.605162	-1.267887	4.304162
C	3.357670	-3.629703	-2.385392
H	4.401816	-3.391046	-2.615907
H	2.920203	-4.082370	-3.280646
H	3.356074	-4.380204	-1.588411

C	-5.229606	-1.545851	-2.491403
H	-5.653065	-2.032151	-1.606776
H	-5.076269	-2.309322	-3.260737
H	-5.979399	-0.843926	-2.872259
Ca	0.194773	2.717514	-0.523840
H	-0.552546	-1.229028	-2.298985
H	-0.560916	4.593807	0.170582
C	3.395512	2.675164	0.061532
C	3.021196	4.020338	0.029431
C	2.612320	4.603175	-1.173875
C	2.583730	3.838454	-2.343010
C	2.951325	2.489679	-2.306653
C	3.351993	1.906527	-1.104219
H	3.706556	2.211784	0.992048
H	3.029053	4.613140	0.938096
H	2.295701	5.639974	-1.190916
H	2.910413	1.888471	-3.209211
H	2.270135	4.291691	-3.278364
H	3.614570	0.855165	-1.065642

88

A4*

Al	-0.000017	-0.351432	-0.251323
N	1.441604	0.548233	-1.109260
N	-1.441663	0.548261	-1.109193
C	-0.000060	1.462388	-2.813281
H	-0.000077	1.991346	-3.757210
C	1.263811	1.219098	-2.245103
C	2.743488	0.473429	-0.505117
C	-1.263911	1.219108	-2.245053
C	-2.743527	0.473446	-0.505012
C	3.128559	1.464182	0.418328
C	3.579038	-0.621932	-0.792996
C	2.469253	1.772179	-2.961444
H	3.013849	2.467029	-2.316794
H	2.180363	2.290598	-3.875471
H	3.165349	0.967056	-3.212076
C	-2.469388	1.772106	-2.961400
H	-3.165158	0.966836	-3.212478
H	-2.180493	2.290950	-3.875185
H	-3.014354	2.466524	-2.316599
C	2.221761	2.623949	0.802603
H	1.266224	2.488569	0.289322
C	-3.128522	1.464142	0.418526
C	-3.579138	-0.621850	-0.792963
C	5.238968	0.298791	0.715127
H	6.213960	0.234712	1.188040
C	4.389041	1.356942	1.009258
H	4.707527	2.112891	1.721120
C	4.828822	-0.685662	-0.174210
H	5.486980	-1.524012	-0.384878
C	-2.221663	2.623853	0.802826
H	-1.266106	2.488382	0.289608
C	2.809835	3.974033	0.368795
H	3.761366	4.174763	0.873475

H	2.119793	4.785157	0.623165
H	2.990156	4.012659	-0.710035
C	-4.828904	-0.685574	-0.174138
H	-5.487106	-1.523876	-0.384851
C	-1.926298	2.613494	2.308530
H	-2.835541	2.777395	2.897726
H	-1.220311	3.412631	2.556701
H	-1.479879	1.661485	2.609067
C	1.926320	2.613565	2.308294
H	1.479799	1.661586	2.608773
H	1.220398	3.412759	2.556462
H	2.835555	2.777358	2.897530
C	-4.388991	1.356914	1.009484
H	-4.707423	2.112825	1.721412
C	3.134858	-1.759367	-1.699407
H	2.134442	-1.526451	-2.071491
C	-5.238976	0.298826	0.715293
H	-6.213956	0.234753	1.188231
C	-3.135028	-1.759240	-1.699463
H	-2.134676	-1.526266	-2.071684
C	3.034211	-3.064378	-0.893449
H	4.022817	-3.484041	-0.679632
H	2.457542	-3.824633	-1.430886
H	2.577739	-2.874717	0.086878
C	-3.034193	-3.064252	-0.893532
H	-2.577544	-2.874569	0.086706
H	-2.457583	-3.824482	-1.431068
H	-4.022750	-3.483944	-0.679543
C	-2.809614	3.973957	0.368922
H	-2.989791	4.012565	-0.709934
H	-2.119560	4.785051	0.623358
H	-3.761198	4.174744	0.873478
C	-4.054213	-1.949762	-2.912418
H	-5.074750	-2.202878	-2.606050
H	-3.683703	-2.762941	-3.544722
H	-4.105537	-1.044077	-3.523768
C	4.053877	-1.949844	-2.912494
H	4.105006	-1.044181	-3.523891
H	3.683358	-2.763090	-3.544707
H	5.074488	-2.202836	-2.606265
Ca	0.000053	-3.373923	0.741659
H	-0.000087	-1.855600	-1.092081
H	0.000041	-5.467409	0.466951
C	0.000059	-0.955548	1.588432
C	1.215805	-1.299145	2.318747
C	1.198286	-1.516024	3.692501
C	0.000123	-1.585236	4.412887
C	-1.198070	-1.516065	3.692549
C	-1.215646	-1.299191	2.318793
H	0.000024	0.616622	1.285905
H	2.184039	-1.202181	1.825883
H	2.143407	-1.672076	4.209020
H	-2.143166	-1.672148	4.209105
H	0.000147	-1.774967	5.480132
H	-2.183904	-1.202255	1.825968

88
A5

Al	0.066854	0.121490	-0.764123
N	1.736828	-1.038876	-0.281295
N	-1.070939	-1.348927	-0.172798
C	0.574528	-3.119606	-0.353497
H	0.702735	-4.194596	-0.382939
C	1.763325	-2.349213	-0.328849
C	2.932016	-0.295725	-0.041121
C	-0.720586	-2.651247	-0.165654
C	-2.423566	-1.040353	0.189956
C	3.755239	0.120323	-1.100950
C	3.211881	0.083278	1.289870
C	3.060566	-3.125290	-0.348376
H	3.273651	-3.418687	-1.382372
H	2.974408	-4.039469	0.243327
H	3.903785	-2.537613	0.015803
C	-1.784232	-3.698339	0.081430
H	-2.346975	-3.480122	0.993364
H	-1.333953	-4.686730	0.174721
H	-2.507038	-3.722506	-0.737728
C	3.499461	-0.292690	-2.542187
H	2.582258	-0.886707	-2.563118
C	-3.458195	-1.119536	-0.760072
C	-2.680700	-0.616015	1.509022
C	5.156360	1.296243	0.499380
H	6.023623	1.914940	0.708605
C	4.865999	0.917411	-0.803088
H	5.517364	1.242720	-1.609773
C	4.331456	0.875359	1.538376
H	4.566841	1.171377	2.556131
C	-3.195912	-1.492120	-2.213005
H	-2.178495	-1.887928	-2.280411
C	4.648645	-1.154564	-3.086468
H	5.577337	-0.577653	-3.160464
H	4.405630	-1.524004	-4.088346
H	4.846249	-2.016014	-2.441895
C	-3.999461	-0.355087	1.884782
H	-4.219755	-0.043019	2.900906
C	-3.259939	-0.253228	-3.118170
H	-4.248381	0.217641	-3.063223
H	-3.074070	-0.535941	-4.159681
H	-2.512688	0.486686	-2.825087
C	3.265827	0.924280	-3.445708
H	2.426300	1.519117	-3.077177
H	3.026861	0.600104	-4.464027
H	4.154204	1.564353	-3.495219
C	-4.758257	-0.815923	-0.348591
H	-5.569926	-0.863196	-1.068632
C	2.334190	-0.405132	2.431791
H	1.361614	-0.653960	2.000238
C	-5.036511	-0.457614	0.964218
H	-6.056732	-0.241775	1.266202
C	-1.533959	-0.392703	2.484118

H	-0.653378	-0.149669	1.882365
C	2.106873	0.649560	3.517794
H	3.020654	0.871185	4.078772
H	1.367350	0.289901	4.240775
H	1.745073	1.597389	3.103009
C	-1.774381	0.794119	3.425123
H	-2.132379	1.677861	2.880351
H	-0.849474	1.058656	3.947149
H	-2.522818	0.572259	4.193073
C	-4.158282	-2.572972	-2.726770
H	-4.200599	-3.439696	-2.059655
H	-3.838491	-2.918552	-3.714796
H	-5.177989	-2.186814	-2.831265
C	-1.186016	-1.659531	3.275474
H	-2.049915	-2.007575	3.852770
H	-0.364989	-1.461854	3.973411
H	-0.866815	-2.465486	2.608765
C	2.900681	-1.691171	3.051334
H	2.988581	-2.486990	2.307304
H	2.247200	-2.051640	3.853364
H	3.895750	-1.512597	3.474363
Ca	0.016696	3.116509	0.968876
H	0.735739	1.101690	0.482636
H	0.471480	4.535919	2.450295
C	-1.297051	1.756669	-0.995238
C	-0.690496	2.754861	-1.798380
C	-1.085487	4.097894	-1.797447
C	-2.141417	4.513970	-0.980766
C	-2.819588	3.554774	-0.225520
C	-2.406001	2.214871	-0.245080
H	0.537662	0.195851	-2.302864
H	0.146285	2.464383	-2.434209
H	-0.568699	4.819335	-2.424865
H	-3.664902	3.849387	0.390500
H	-2.440581	5.556469	-0.949477
H	-2.965884	1.515324	0.367342

85

LAl(H)C_6H_5

Al	-0.039172	-0.799693	0.696919
N	-1.386633	-0.800367	-0.688345
N	1.424314	-0.949127	-0.539086
C	0.034889	-2.062087	-2.157876
H	0.041550	-2.701933	-3.030935
C	-1.214055	-1.517379	-1.791623
C	-2.657426	-0.197647	-0.402761
C	1.283456	-1.719934	-1.622858
C	2.707637	-0.377028	-0.251772
C	-3.668549	-0.957117	0.211171
C	-2.819948	1.177530	-0.661621
C	-2.383157	-1.783591	-2.706266
H	-3.022147	-2.556862	-2.267947
H	-2.044047	-2.128442	-3.683450
H	-3.001156	-0.891736	-2.831664
C	-0.087757	0.876521	1.744953

H	-0.190250	-2.175185	1.500420
C	-1.294321	1.271168	2.355196
H	-2.185076	0.653268	2.253273
C	-1.406445	2.452866	3.086237
H	-2.358943	2.724188	3.533194
C	2.517000	-2.246460	-2.312671
H	3.193384	-1.437759	-2.597573
H	2.256733	-2.822246	-3.201005
H	3.071701	-2.889690	-1.621162
C	-3.464911	-2.409335	0.618610
H	-2.520680	-2.759153	0.191681
C	3.549311	-0.979417	0.698686
C	3.067914	0.823687	-0.894981
C	-5.077145	1.017065	0.214136
H	-6.025016	1.491634	0.448728
C	0.914993	2.916472	2.664419
H	1.789132	3.550972	2.783571
C	1.013042	1.730876	1.935325
H	1.979370	1.476438	1.506507
C	-4.878706	-0.324213	0.507390
H	-5.673703	-0.889704	0.984725
C	-0.299209	3.284913	3.238394
H	-0.380827	4.208567	3.804191
C	-4.050221	1.759784	-0.357916
H	-4.206120	2.815710	-0.549179
C	3.148105	-2.248905	1.432504
H	2.245587	-2.644941	0.957728
C	-4.583015	-3.326481	0.104172
H	-5.538419	-3.109834	0.593817
H	-4.337578	-4.372483	0.313323
H	-4.734409	-3.219108	-0.974591
C	4.304060	1.392159	-0.585739
H	4.605818	2.313838	-1.075115
C	2.795879	-1.936393	2.894228
H	3.673785	-1.565392	3.435409
H	2.433779	-2.836469	3.400919
H	2.013675	-1.174070	2.964051
C	-3.334443	-2.521392	2.145035
H	-2.502145	-1.917291	2.516155
H	-3.146892	-3.560090	2.436047
H	-4.252397	-2.186505	2.641188
C	4.767788	-0.361871	0.989942
H	5.427274	-0.805466	1.730164
C	-1.680346	2.009221	-1.234082
H	-0.749105	1.578660	-0.853759
C	5.150365	0.809563	0.350245
H	6.103765	1.272611	0.585145
C	2.146920	1.518443	-1.889042
H	1.192852	0.983992	-1.894750
C	-1.713696	3.465275	-0.761296
H	-2.559145	4.018015	-1.185775
H	-0.802579	3.978647	-1.078689
H	-1.768707	3.520045	0.330091
C	1.858503	2.965261	-1.469699
H	1.409956	3.007128	-0.473281

H	1.165502	3.428694	-2.179329
H	2.770246	3.571978	-1.459694
C	4.225161	-3.337904	1.345863
H	4.499482	-3.548053	0.306977
H	3.858353	-4.265289	1.797003
H	5.137252	-3.050393	1.879760
C	2.710006	1.475833	-3.316723
H	3.669479	2.001457	-3.378206
H	2.015575	1.959791	-4.011546
H	2.866390	0.449812	-3.661639
C	-1.634633	1.928158	-2.766307
H	-1.475520	0.902962	-3.112960
H	-0.813237	2.539492	-3.155543
H	-2.569354	2.296821	-3.203837

15
B1

Ca	-1.859518	-0.000140	-0.000127
H	-2.440648	2.044198	0.000126
H	-2.439774	-2.044733	0.000028
C	1.006293	-0.699344	1.208975
C	1.006094	0.698685	1.209431
C	0.998634	1.399324	0.000526
C	1.006398	0.699504	-1.208866
C	1.006603	-0.698527	-1.209322
C	0.999067	-1.399165	-0.000406
H	1.000687	-1.244425	2.147296
H	1.000247	1.243163	2.148104
H	0.964330	2.482676	0.000891
H	1.001222	-1.243018	-2.147988
H	1.000778	1.244596	-2.147181
H	0.964988	-2.482525	-0.000766

15
B2*

Ca	1.633556	-0.091422	-0.000089
H	3.558029	0.762661	-0.000910
H	0.726639	-2.073148	0.000565
C	-0.983330	-0.609248	-1.214080
C	-0.845841	0.769063	-1.206424
C	-0.684237	1.487424	-0.000382
C	-0.845438	0.769881	1.206177
C	-0.982926	-0.608456	1.214573
C	-0.849995	-1.373607	0.000443
H	-1.113080	-1.134844	-2.154379
H	-0.884418	1.312003	-2.147135
H	-0.612064	2.568421	-0.000830
H	-1.112373	-1.133520	2.155212
H	-0.883670	1.313379	2.146578
H	-1.199577	-2.396842	0.000829

15
B3

Ca	1.609412	-0.000192	-0.323396
H	3.676476	-0.000352	-0.704985

H	-1.558812	-0.001683	-2.163613
C	-0.893590	1.224317	-0.502350
C	-0.535842	1.215383	0.833368
C	-0.391229	0.001222	1.562576
C	-0.535701	-1.214105	0.835264
C	-0.893248	-1.225167	-0.500499
C	-1.594757	-0.000929	-1.069503
H	-0.958688	2.168985	-1.036091
H	-0.252410	2.152637	1.313129
H	-0.149947	0.002051	2.618837
H	-0.958151	-2.170662	-1.032811
H	-0.252314	-2.150516	1.316666
H	-2.668190	-0.000939	-0.796351

	88		
B4			
Al	0.725232	0.283885	-0.691572
N	2.471879	0.204495	0.263349
N	0.250656	1.865910	0.432770
C	2.365495	2.259897	1.498818
H	2.915776	2.929399	2.147647
C	3.015113	1.081628	1.108508
C	3.202952	-0.995574	-0.041020
C	1.041116	2.609934	1.207672
C	-1.149004	2.189399	0.377028
C	2.898843	-2.177172	0.659492
C	4.156786	-0.985803	-1.076996
C	4.386758	0.819364	1.681489
H	4.423486	-0.163083	2.160182
H	4.658137	1.582119	2.412005
H	5.139090	0.809691	0.887356
C	0.494062	3.861964	1.849415
H	-0.039221	4.473760	1.117356
H	1.294770	4.452016	2.297167
H	-0.226870	3.604379	2.632128
C	1.837675	-2.227554	1.750266
H	1.402781	-1.228154	1.849155
C	-1.989418	1.717253	1.406546
C	-1.669351	2.881466	-0.728716
C	4.548726	-3.347040	-0.680932
H	5.075972	-4.263134	-0.928530
C	3.593615	-3.342197	0.326187
H	3.377852	-4.263288	0.860511
C	4.818547	-2.176305	-1.379160
H	5.553692	-2.191284	-2.178462
C	-1.437304	0.913278	2.575428
H	-0.363607	1.108134	2.649969
C	2.438423	-2.596335	3.113347
H	2.866580	-3.604597	3.101670
H	1.665052	-2.570301	3.888219
H	3.231800	-1.899655	3.401943
C	-3.051266	3.081737	-0.793503
H	-3.473479	3.609129	-1.644470
C	-1.607785	-0.587680	2.309547

H	-2.665617	-0.856105	2.221283
H	-1.166932	-1.175415	3.122830
H	-1.125703	-0.886742	1.372462
C	0.701534	-3.186326	1.367914
H	0.241828	-2.884811	0.421351
H	-0.076966	-3.184079	2.137565
H	1.067455	-4.213272	1.259796
C	-3.360555	1.944064	1.299551
H	-4.028239	1.568079	2.068109
C	4.444217	0.266796	-1.892460
H	3.985778	1.117360	-1.379532
C	-3.893122	2.617707	0.206423
H	-4.964950	2.774273	0.134137
C	-0.782533	3.417897	-1.841559
H	0.253784	3.168121	-1.594401
C	3.791352	0.164079	-3.279128
H	4.216951	-0.671522	-3.846081
H	3.956999	1.083382	-3.851207
H	2.712567	-0.001528	-3.195248
C	-1.109152	2.754777	-3.186379
H	-1.012472	1.667232	-3.115385
H	-0.424670	3.110919	-3.963624
H	-2.130841	2.989355	-3.505579
C	-2.058744	1.305580	3.920894
H	-1.994594	2.384762	4.094553
H	-1.534422	0.796357	4.735833
H	-3.113039	1.016981	3.983609
C	-0.872269	4.947159	-1.944591
H	-1.880000	5.268854	-2.229071
H	-0.176642	5.318794	-2.704086
H	-0.625929	5.428626	-0.992878
C	5.944856	0.561275	-2.017754
H	6.435222	0.592004	-1.039443
H	6.098242	1.528605	-2.506684
H	6.456907	-0.194992	-2.621936
Ca	-4.768843	-3.452115	-1.874258
H	-5.762883	-4.822368	-3.126637
H	-2.928741	-0.404681	-2.196751
C	-2.462963	-2.157544	-1.007470
C	-2.893332	-2.966034	0.024732
C	-4.139703	-2.754982	0.686042
C	-4.959953	-1.697234	0.198475
C	-4.559567	-0.874873	-0.837542
C	-3.078610	-0.774983	-1.177071
H	-1.517090	-2.365763	-1.500360
H	-2.310030	-3.852441	0.276888
H	-4.446236	-3.369064	1.524488
H	-5.231525	-0.098420	-1.194081
H	-5.976097	-1.603731	0.583380
H	-2.575427	-0.040666	-0.525350

88

B5*

Al	0.042623	0.086992	-0.441792
N	-1.335037	-1.217588	0.141510

N	1.465446	-1.079508	0.210071
C	0.142164	-3.043092	0.637667
H	0.183572	-4.086763	0.922372
C	-1.135990	-2.490759	0.465721
C	-2.663188	-0.680655	0.246918
C	1.357139	-2.357057	0.592158
C	2.772886	-0.483157	0.216226
C	-3.009996	-0.043254	1.457218
C	-3.555135	-0.742603	-0.834676
C	-2.302254	-3.430114	0.667857
H	-2.560201	-3.877442	-0.298117
H	-3.190988	-2.921627	1.042917
H	-2.034538	-4.238351	1.350366
C	2.585569	-3.126229	1.022378
H	3.175476	-3.431329	0.153815
H	2.298165	-4.024158	1.570551
H	3.235503	-2.515088	1.652183
C	-2.037866	-0.011430	2.631392
H	-1.027966	-0.086696	2.217779
C	3.092737	0.413231	1.255016
C	3.668707	-0.749671	-0.833801
C	-5.161917	0.508810	0.486750
H	-6.136976	0.977602	0.577459
C	-4.271631	0.540331	1.556182
H	-4.564016	1.037851	2.475574
C	-4.803394	-0.128814	-0.692266
H	-5.508741	-0.157599	-1.518231
C	2.086562	0.719845	2.360391
H	1.106166	0.827765	1.881523
C	-2.243623	-1.219969	3.557074
H	-3.259493	-1.223441	3.968189
H	-1.536304	-1.183536	4.392536
H	-2.087749	-2.165030	3.031288
C	4.900982	-0.091685	-0.826632
H	5.606678	-0.268416	-1.632992
C	2.356650	2.042801	3.080960
H	3.240015	1.983549	3.726976
H	1.502661	2.291449	3.719115
H	2.499693	2.864093	2.373179
C	-2.091561	1.286671	3.443702
H	-1.999478	2.168429	2.803542
H	-1.264512	1.302917	4.161308
H	-3.019735	1.375370	4.018715
C	4.343666	1.029687	1.226589
H	4.620922	1.724827	2.011497
C	-3.229664	-1.490576	-2.120435
H	-2.235643	-1.933600	-2.008348
C	5.241451	0.786344	0.192621
H	6.205564	1.285451	0.182068
C	3.341845	-1.715385	-1.969473
H	2.472266	-2.309003	-1.676576
C	-3.176087	-0.566290	-3.343618
H	-4.130822	-0.049789	-3.493511
H	-2.965610	-1.150042	-4.245921
H	-2.392571	0.185575	-3.237219

C	2.951391	-0.987939	-3.264424
H	1.998643	-0.466355	-3.152240
H	2.844396	-1.709130	-4.081813
H	3.715605	-0.257533	-3.553095
C	1.968679	-0.416340	3.387466
H	1.593896	-1.338958	2.939155
H	1.269520	-0.131564	4.181630
H	2.940117	-0.624547	3.849895
C	4.497056	-2.691709	-2.240473
H	5.345743	-2.189737	-2.716903
H	4.166649	-3.484898	-2.918654
H	4.863889	-3.156540	-1.319767
C	-4.238319	-2.624758	-2.365588
H	-4.354056	-3.265070	-1.486521
H	-3.914102	-3.247031	-3.206191
H	-5.227929	-2.225027	-2.612504
Ca	-0.658306	4.523607	-1.500697
H	-0.945754	6.573695	-1.957501
H	-0.187865	1.433973	-3.341050
C	-1.247687	2.049235	-1.511034
C	-0.880897	1.947793	-0.002456
C	0.268537	2.828943	0.337796
C	1.316512	2.654326	-0.530768
C	1.160790	1.597266	-1.525791
C	-0.169280	1.445080	-2.249468
H	-2.257178	1.726342	-1.761818
H	-1.747097	2.047391	0.649871
H	0.347821	3.394195	1.265550
H	2.038972	1.378239	-2.126054
H	2.294583	3.106216	-0.372557
H	-0.327033	-0.043212	-2.172364

88
B6

Al	-0.046050	-0.359991	-1.011585
N	-1.490994	-1.251718	-0.099384
N	1.329871	-1.380548	-0.131822
C	-0.159285	-3.254896	0.064116
H	-0.205055	-4.327986	0.195253
C	-1.386019	-2.571523	0.082770
C	-2.768552	-0.635548	0.130746
C	1.121018	-2.675306	0.094458
C	2.631640	-0.820012	0.094393
C	-3.034491	-0.090984	1.404236
C	-3.704769	-0.560048	-0.913421
C	-2.629100	-3.386293	0.341978
H	-3.202212	-2.976510	1.177572
H	-2.376832	-4.424373	0.558447
H	-3.282782	-3.356502	-0.535226
C	2.290079	-3.564993	0.427986
H	3.051803	-3.504758	-0.354565
H	1.973828	-4.601880	0.541745
H	2.765275	-3.237495	1.356845
C	-1.952773	-0.035773	2.478181
H	-1.013935	0.181990	1.956475

C	2.935106	-0.310644	1.368728
C	3.540226	-0.721442	-0.974394
C	-5.252899	0.505929	0.616020
H	-6.226987	0.943704	0.811057
C	-4.297401	0.458782	1.626248
H	-4.538349	0.880160	2.595648
C	-4.948199	0.019481	-0.646174
H	-5.686349	0.091947	-1.439208
C	1.965526	-0.419302	2.535620
H	1.078169	-0.958572	2.190963
C	-1.769385	-1.361948	3.230377
H	-2.704693	-1.670013	3.711584
H	-1.010680	-1.242348	4.011913
H	-1.434746	-2.168858	2.574931
C	4.756231	-0.074235	-0.746489
H	5.468455	0.026169	-1.560526
C	1.505686	0.963919	3.010698
H	2.354507	1.577100	3.332038
H	0.829968	0.858892	3.865489
H	0.983826	1.507880	2.219502
C	-2.179417	1.098058	3.482337
H	-2.351352	2.052114	2.976999
H	-1.297769	1.211615	4.118050
H	-3.029840	0.894781	4.142764
C	4.165694	0.321180	1.549371
H	4.417199	0.731070	2.523516
C	-3.376244	-1.030986	-2.321905
H	-2.465469	-1.635012	-2.280187
C	5.068898	0.449073	0.501841
H	6.017610	0.953042	0.658794
C	3.223495	-1.267457	-2.359518
H	2.290949	-1.835781	-2.298563
C	-3.074934	0.178791	-3.219331
H	-3.959883	0.816599	-3.323020
H	-2.763675	-0.148405	-4.216127
H	-2.267215	0.789668	-2.803570
C	2.992805	-0.129599	-3.364146
H	2.164063	0.513951	-3.055078
H	2.743493	-0.536181	-4.349251
H	3.888874	0.492993	-3.465766
C	2.568110	-1.220028	3.698786
H	2.907502	-2.210021	3.378583
H	1.822178	-1.357195	4.488690
H	3.426414	-0.700344	4.138324
C	4.314838	-2.221872	-2.863699
H	5.259545	-1.697402	-3.042337
H	4.007344	-2.679113	-3.809437
H	4.511896	-3.023864	-2.145164
C	-4.478717	-1.902350	-2.936469
H	-4.741394	-2.741159	-2.283899
H	-4.142051	-2.308938	-3.895095
H	-5.392708	-1.330184	-3.127216
Ca	-0.005236	4.293290	-1.605684
H	-0.429127	5.419115	-3.336728
H	-0.237656	1.854600	-1.941252

C	-0.955728	2.323807	0.082876
C	-0.630216	3.379887	0.889121
C	0.661766	4.018510	0.892356
C	1.700180	3.251676	0.249157
C	1.458277	2.196367	-0.584758
C	0.047083	1.650526	-0.865103
H	-1.953408	1.904048	0.159935
H	-1.404052	3.774666	1.546183
H	0.916251	4.715208	1.681769
H	2.314090	1.665987	-0.988781
H	2.738567	3.530700	0.420726
H	-0.073525	-0.839656	-2.542022

88

B7*

Al	0.290460	-0.496836	-0.866923
N	-0.941229	-1.337014	0.394469
N	1.841114	-0.921445	0.227070
C	0.762789	-2.869866	1.123133
H	0.936444	-3.831830	1.589199
C	-0.575405	-2.486752	0.971165
C	-2.291321	-0.869919	0.480709
C	1.902970	-2.076290	0.877536
C	3.009899	-0.093054	0.122210
C	-2.633185	0.011066	1.529335
C	-3.228679	-1.209053	-0.520801
C	-1.624246	-3.460860	1.453682
H	-1.259995	-4.047863	2.298376
H	-1.850636	-4.152736	0.634705
H	-2.555641	-2.967221	1.733938
C	3.229787	-2.596029	1.372577
H	3.798722	-2.997772	0.527648
H	3.090553	-3.391517	2.105307
H	3.833401	-1.799662	1.813099
C	-1.665413	0.294140	2.670585
H	-0.653816	0.222567	2.261102
C	3.154263	0.969730	1.035200
C	3.948375	-0.315271	-0.899475
C	-4.829806	0.294188	0.522710
H	-5.807657	0.764291	0.519219
C	-3.910537	0.587115	1.524176
H	-4.190507	1.276707	2.313443
C	-4.491901	-0.611228	-0.480898
H	-5.217372	-0.833501	-1.256938
C	2.088568	1.237050	2.085581
H	1.149312	0.875050	1.662065
C	-1.778665	-0.772768	3.771892
H	-2.800362	-0.816445	4.165453
H	-1.104416	-0.528285	4.599131
H	-1.508886	-1.766060	3.409792
C	5.068711	0.518205	-0.955540
H	5.806804	0.366724	-1.737777
C	1.905937	2.726392	2.389708
H	2.751861	3.136822	2.952010
H	1.009979	2.871385	3.002375

H	1.781519	3.304031	1.469820
C	-1.835626	1.683206	3.296255
H	-1.872974	2.476483	2.544381
H	-0.993385	1.888281	3.964155
H	-2.746536	1.745113	3.901640
C	4.293805	1.767948	0.945793
H	4.430594	2.588936	1.641956
C	-2.919354	-2.218510	-1.617959
H	-1.897581	-2.578206	-1.468659
C	5.251607	1.541324	-0.036340
H	6.130859	2.175344	-0.094891
C	3.755601	-1.388861	-1.959906
H	2.910804	-2.017475	-1.663535
C	-2.979023	-1.596927	-3.020079
H	-3.955600	-1.143046	-3.223643
H	-2.802403	-2.365048	-3.779259
H	-2.200932	-0.836108	-3.136490
C	3.386635	-0.746773	-3.305171
H	2.485663	-0.134527	-3.212661
H	3.191792	-1.518711	-4.056698
H	4.199270	-0.108143	-3.669202
C	2.331533	0.443513	3.376700
H	2.322684	-0.635167	3.197528
H	1.546844	0.663927	4.109598
H	3.296882	0.709288	3.821865
C	4.984569	-2.295391	-2.112193
H	5.838905	-1.750461	-2.527548
H	4.760121	-3.121467	-2.794387
H	5.298853	-2.718943	-1.152985
C	-3.871002	-3.421585	-1.522568
H	-3.884444	-3.849383	-0.515606
H	-3.562179	-4.202579	-2.224413
H	-4.897496	-3.135360	-1.775090
Ca	-2.928544	1.885921	-1.597238
H	-4.750423	2.103345	-2.660366
H	-1.252026	0.454923	-1.526063
C	-0.592234	2.273398	-0.056399
C	-1.115530	3.537958	-0.297453
C	-1.189059	4.063830	-1.600625
C	-0.454435	3.364255	-2.605951
C	0.114253	2.135333	-2.372717
C	-0.140044	1.392309	-1.133918
H	-0.501948	1.945454	0.969925
H	-1.458729	4.139205	0.543870
H	-1.563419	5.064235	-1.782743
H	0.702370	1.668573	-3.159055
H	-0.301725	3.844337	-3.569725
H	0.429526	-1.358786	-2.205588

88

B8

Al	0.015793	0.313893	-0.613959
N	-1.377040	-0.959986	-0.863810
N	1.446962	-0.946876	-0.708851
C	0.089623	-2.602696	-1.822092

H	0.126621	-3.533597	-2.372486
C	-1.184464	-2.112136	-1.510648
C	-2.670237	-0.661981	-0.308563
C	1.319387	-2.107176	-1.356619
C	2.734969	-0.581589	-0.173251
C	-2.935590	-1.090170	1.007319
C	-3.597142	0.098418	-1.040402
C	-2.375547	-2.935189	-1.925511
H	-3.104397	-3.025811	-1.117802
H	-2.072326	-3.930305	-2.250755
H	-2.882913	-2.435127	-2.756717
C	2.536458	-2.958775	-1.612110
H	3.097789	-2.545802	-2.455001
H	2.248069	-3.980691	-1.858767
H	3.208710	-2.970493	-0.752175
C	-1.941724	-1.927566	1.800832
H	-1.001742	-1.957176	1.243047
C	2.983540	-0.826647	1.194304
C	3.687974	0.037310	-1.000619
C	-5.085258	0.024253	0.875449
H	-6.027273	0.298476	1.339840
C	-4.159363	-0.739817	1.576859
H	-4.390783	-1.060838	2.588189
C	-4.802490	0.437073	-0.419392
H	-5.528370	1.037706	-0.959242
C	1.911041	-1.420165	2.102950
H	0.952256	-0.997457	1.784893
C	-2.429144	-3.377086	1.942119
H	-3.371719	-3.421921	2.498684
H	-1.688277	-3.976318	2.481781
H	-2.592477	-3.844265	0.966528
C	4.920541	0.380062	-0.437550
H	5.667536	0.871917	-1.052894
C	2.092594	-1.032322	3.573653
H	2.959846	-1.527666	4.023445
H	1.213623	-1.337765	4.147226
H	2.209727	0.048642	3.692681
C	-1.635023	-1.323297	3.175861
H	-1.232759	-0.310372	3.085158
H	-0.896128	-1.942924	3.694049
H	-2.526681	-1.281240	3.809789
C	4.236285	-0.480396	1.700404
H	4.462505	-0.656994	2.745805
C	-3.326772	0.564381	-2.462081
H	-2.350604	0.172913	-2.765678
C	5.200770	0.114014	0.893128
H	6.164776	0.385846	1.311335
C	3.422959	0.370493	-2.461749
H	2.444440	-0.036658	-2.734299
C	-3.261315	2.094119	-2.544146
H	-4.234071	2.546599	-2.323462
H	-2.966939	2.411614	-3.549449
H	-2.549421	2.527627	-1.836513
C	3.368363	1.889745	-2.672601
H	2.684275	2.385588	-1.979130

H	3.055494	2.119649	-3.696524
H	4.351862	2.344233	-2.511290
C	1.802221	-2.946099	1.970857
H	1.513581	-3.253423	0.962785
H	1.040538	-3.328008	2.659725
H	2.755280	-3.426558	2.218310
C	4.471329	-0.250922	-3.397925
H	5.453546	0.210344	-3.251567
H	4.185121	-0.087175	-4.441570
H	4.589366	-1.327196	-3.240083
C	-4.376894	0.020275	-3.442653
H	-4.472815	-1.068298	-3.381507
H	-4.106013	0.284370	-4.469698
H	-5.364150	0.449244	-3.240928
Ca	0.119472	3.882925	-0.512081
H	-1.782351	4.743759	-0.962110
H	2.201220	4.350888	-0.601660
C	-1.388921	1.966606	1.417863
C	-1.502345	2.912169	2.438600
C	-0.363446	3.354748	3.109882
C	0.885024	2.850975	2.747668
C	0.987774	1.906868	1.724218
C	-0.143279	1.422543	1.032146
H	-2.299996	1.667182	0.906678
H	-2.477646	3.316393	2.689210
H	-0.446084	4.100343	3.894419
H	1.982170	1.565531	1.449482
H	1.783541	3.208754	3.240435
H	0.098454	1.405233	-1.817683

88

B9*

Al	0.074329	0.462932	0.424493
N	-1.173466	-0.932985	1.007455
N	1.615492	-0.634753	0.942255
C	0.392581	-2.037247	2.460843
H	0.490282	-2.756835	3.263441
C	-0.903843	-1.812977	1.971443
C	-2.495398	-0.905208	0.448390
C	1.587550	-1.565515	1.891715
C	2.850952	-0.355567	0.267914
C	-3.490731	-0.092500	1.019957
C	-2.751948	-1.670848	-0.709861
C	-2.021416	-2.633363	2.569137
H	-2.711449	-1.984592	3.114802
H	-1.629661	-3.383635	3.256290
H	-2.606051	-3.132071	1.792106
C	2.871251	-2.208567	2.354447
H	2.733212	-2.687466	3.324873
H	3.687529	-1.486912	2.417003
H	3.177116	-2.976607	1.637110
C	-3.207064	0.860688	2.171707
H	-2.158415	0.741159	2.459032
C	3.635747	0.744058	0.663611
C	3.230443	-1.168616	-0.817664

C	-5.062670	-0.925558	-0.627181
H	-6.066404	-0.946013	-1.039991
C	-4.773362	-0.123577	0.465925
H	-5.555641	0.495396	0.896305
C	-4.052683	-1.679842	-1.215040
H	-4.283796	-2.267643	-2.096289
C	3.230256	1.662982	1.805962
H	2.219807	1.384527	2.118004
C	-4.072221	0.585712	3.409119
H	-5.136207	0.724337	3.189575
H	-3.806801	1.276348	4.215865
H	-3.942442	-0.433770	3.782506
C	4.427571	-0.881964	-1.475114
H	4.737525	-1.499383	-2.313645
C	3.181506	3.128040	1.349821
H	4.184521	3.538680	1.191807
H	2.679183	3.754352	2.093780
H	2.648845	3.218925	0.395601
C	-3.399881	2.314421	1.709734
H	-2.922373	2.468107	0.735592
H	-2.982256	3.018444	2.437315
H	-4.461211	2.554014	1.581487
C	4.827460	0.988076	-0.022883
H	5.451745	1.825514	0.275624
C	-1.635849	-2.426169	-1.426033
H	-0.737714	-1.804215	-1.340764
C	5.228380	0.182229	-1.079952
H	6.158634	0.387630	-1.600403
C	2.363804	-2.319837	-1.307070
H	1.513351	-2.420859	-0.626327
C	-1.921440	-2.617593	-2.920316
H	-2.703057	-3.365963	-3.092611
H	-1.020940	-2.973910	-3.428021
H	-2.233183	-1.684458	-3.399479
C	1.800445	-2.011372	-2.700064
H	1.218513	-1.085889	-2.687276
H	1.150484	-2.825528	-3.036306
H	2.603943	-1.898000	-3.435847
C	4.151732	1.508154	3.023837
H	4.134491	0.485921	3.413080
H	3.834500	2.179756	3.828100
H	5.188481	1.753484	2.768416
C	3.115040	-3.657912	-1.315870
H	3.927688	-3.656733	-2.050208
H	2.431906	-4.471993	-1.579506
H	3.552643	-3.885327	-0.339402
C	-1.310268	-3.783865	-0.786364
H	-0.921299	-3.678983	0.228798
H	-0.544333	-4.298927	-1.377162
H	-2.199438	-4.423886	-0.754680
Ca	-0.333700	3.551180	0.067567
H	-0.240420	5.388623	1.136700
H	-0.135092	0.340570	-1.350142
C	0.283511	1.958495	-2.966288
C	0.033372	3.315430	-2.919357

C	-1.214174	3.839594	-2.498338
C	-2.215550	2.902026	-2.161292
C	-1.989862	1.537610	-2.181382
C	-0.693601	0.981205	-2.524189
H	1.243081	1.606412	-3.334144
H	0.803999	3.998705	-3.271280
H	-1.438330	4.897453	-2.586972
H	-2.806566	0.861537	-1.945692
H	-3.215471	3.256267	-1.916769
H	-0.697217	0.022146	-3.043822

88

C1

Al	0.467331	-0.070799	-0.519125
N	-0.087408	-1.893953	0.082422
N	2.322552	-0.513458	0.054633
C	2.067693	-2.850242	0.537433
H	2.591888	-3.757558	0.809128
C	0.678464	-2.941273	0.400061
C	-1.513388	-2.040027	0.176552
C	2.835410	-1.680633	0.442504
C	3.181843	0.639428	0.028444
C	-2.125741	-1.876308	1.436326
C	-2.277278	-2.275694	-0.979587
C	0.036616	-4.280778	0.668775
H	-0.547958	-4.246977	1.593769
H	0.790768	-5.062704	0.764447
H	-0.656409	-4.546956	-0.133670
C	-2.318077	2.566724	0.776304
H	-2.190416	2.720527	1.841960
C	-3.073253	1.487840	0.310254
H	-3.509284	0.783307	1.010252
C	-3.262092	1.306526	-1.061063
H	-3.844778	0.463869	-1.417177
C	4.299188	-1.775972	0.796915
H	4.919689	-1.566044	-0.079347
H	4.547780	-2.769006	1.172470
H	4.562247	-1.031323	1.553186
C	-1.302868	-1.613203	2.691133
H	-0.271207	-1.914746	2.490182
C	3.172174	1.522885	1.123160
C	3.959033	0.902131	-1.115958
C	-4.289660	-2.142671	0.372126
H	-5.372081	-2.179902	0.447424
C	-1.926254	3.271479	-1.504837
H	-1.474900	3.959794	-2.211619
C	-1.738871	3.454443	-0.132154
H	-1.143923	4.286388	0.230353
C	-3.517560	-1.931220	1.509879
H	-4.010111	-1.803320	2.469206
C	-2.694366	2.202357	-1.969444
H	-2.857995	2.072247	-3.033111
C	-3.668931	-2.318925	-0.856892
H	-4.276831	-2.497264	-1.739812
C	2.304983	1.288053	2.352601

H	1.743515	0.360750	2.201203
C	-1.781960	-2.424408	3.900738
H	-2.762569	-2.091148	4.255921
H	-1.078979	-2.306173	4.731205
H	-1.855521	-3.490769	3.664717
C	4.745958	2.054177	-1.131161
H	5.349027	2.278171	-2.006104
C	1.283686	2.420795	2.527616
H	1.780106	3.382890	2.693130
H	0.637392	2.225321	3.389570
H	0.653276	2.512254	1.636861
C	-1.270697	-0.113927	3.014134
H	-0.880803	0.462869	2.168995
H	-0.632621	0.079245	3.883199
H	-2.276295	0.258811	3.241805
C	3.982556	2.659043	1.064378
H	3.994913	3.349957	1.902743
C	-1.637268	-2.500625	-2.341251
H	-0.551589	-2.489082	-2.205419
C	4.767261	2.925711	-0.049062
H	5.388559	3.815396	-0.078023
C	3.923898	-0.006280	-2.336657
H	3.422279	-0.936120	-2.052933
C	-1.990720	-1.373883	-3.321268
H	-3.071838	-1.325920	-3.496317
H	-1.502900	-1.541341	-4.287103
H	-1.659390	-0.405992	-2.933614
C	3.093340	0.636349	-3.457814
H	2.077731	0.861075	-3.116295
H	3.027698	-0.034690	-4.320976
H	3.551940	1.574528	-3.789768
C	3.150071	1.103217	3.620069
H	3.862764	0.280196	3.507691
H	2.506225	0.880465	4.477375
H	3.719354	2.008587	3.856420
C	5.324870	-0.377300	-2.840476
H	5.850366	0.490224	-3.253038
H	5.251611	-1.123751	-3.637718
H	5.946549	-0.792325	-2.040668
C	-2.012888	-3.871595	-2.921523
H	-1.768776	-4.682537	-2.228143
H	-1.470925	-4.048142	-3.856054
H	-3.083778	-3.933905	-3.142435
Ca	-4.837588	3.919376	-0.436839
H	-5.068207	4.604328	1.569220
H	-5.756179	3.974056	-2.358587

88

C2*

Al	0.000019	-0.346503	-0.720222
N	-1.388950	-1.379115	0.181300
N	1.389026	-1.379068	0.181300
C	0.000071	-3.239962	0.786715
H	0.000091	-4.267116	1.129389
C	-1.252377	-2.625912	0.636321

C	-2.669605	-0.725325	0.216368
C	1.252499	-2.625861	0.636336
C	2.669651	-0.725218	0.216323
C	-2.970355	0.085516	1.331659
C	-3.541228	-0.817490	-0.881442
C	-2.477272	-3.442437	0.965274
H	-3.256900	-2.840475	1.435048
H	-2.227975	-4.282829	1.614234
H	-2.895464	-3.839959	0.033739
C	-0.000081	1.938716	-0.002598
H	-0.000103	1.882126	1.083569
C	-1.223915	2.359008	-0.639186
H	-2.149913	2.402858	-0.073873
C	-1.217658	2.442234	-2.012675
H	-2.136170	2.612398	-2.565728
C	2.477413	-3.442352	0.965311
H	2.895451	-3.840110	0.033806
H	2.228171	-4.282588	1.614494
H	3.257129	-2.840317	1.434843
C	-2.008164	0.209294	2.505735
H	-1.004316	0.010551	2.119425
C	2.970403	0.085651	1.331593
C	3.541233	-0.817349	-0.881523
C	-5.057113	0.697450	0.256776
H	-5.989052	1.253841	0.271891
C	1.217511	2.442345	-2.012639
H	2.136024	2.612590	-2.565665
C	1.223735	2.359124	-0.639150
H	2.149718	2.403059	-0.073813
C	-4.180794	0.778395	1.333716
H	-4.437086	1.407090	2.179973
C	-0.000053	2.230322	-2.707842
H	-0.000036	2.222955	-3.792045
C	-4.733130	-0.088793	-0.839499
H	-5.418202	-0.138195	-1.680968
C	2.008278	0.209358	2.505728
H	1.004420	0.010478	2.119508
C	-2.287579	-0.853369	3.579398
H	-3.309774	-0.756067	3.961696
H	-1.596570	-0.731713	4.420026
H	-2.163601	-1.867823	3.192075
C	4.733093	-0.088578	-0.839643
H	5.418131	-0.137954	-1.681140
C	1.992532	1.606093	3.136945
H	2.921129	1.819881	3.676474
H	1.179161	1.672403	3.865332
H	1.837704	2.401611	2.401965
C	-1.992542	1.606011	3.136995
H	-1.837949	2.401578	2.402017
H	-1.179075	1.672421	3.865266
H	-2.921095	1.819638	3.676665
C	4.180801	0.778601	1.333587
H	4.437100	1.407317	2.179826
C	-3.224982	-1.663884	-2.105285
H	-2.307840	-2.225484	-1.901332

C	5.057075	0.697700	0.256606
H	5.988980	1.254148	0.271672
C	3.225002	-1.663803	-2.105327
H	2.307857	-2.225394	-1.901356
C	-2.955521	-0.781253	-3.332250
H	-3.837681	-0.182832	-3.586119
H	-2.705967	-1.400375	-4.200330
H	-2.118630	-0.100851	-3.146855
C	2.955561	-0.781242	-3.332347
H	2.118673	-0.100823	-3.147000
H	2.706011	-1.400416	-4.200392
H	3.837726	-0.182843	-3.586245
C	2.287922	-0.853235	3.579401
H	2.164128	-1.867718	3.192099
H	1.596919	-0.731695	4.420051
H	3.310113	-0.755735	3.961663
C	4.335792	-2.682772	-2.397031
H	5.261063	-2.187879	-2.710155
H	4.030918	-3.353053	-3.206972
H	4.568063	-3.290037	-1.516524
C	-4.335764	-2.682839	-2.397069
H	-4.568075	-3.290136	-1.516594
H	-4.030858	-3.353090	-3.207021
H	-5.261022	-2.187935	-2.710216
Ca	-0.000213	5.017573	-0.488153
H	-0.000246	6.327734	-2.174572
H	-0.000222	4.982254	1.664263

	88		
C3			
Al	0.000006	0.060227	-0.208299
N	-1.419068	-1.194609	0.074971
N	1.419036	-1.194629	0.074976
C	-0.000030	-3.128371	0.279254
H	-0.000042	-4.204836	0.397445
C	-1.256126	-2.510386	0.233295
C	-2.738346	-0.617704	0.140595
C	1.256074	-2.510405	0.233344
C	2.738313	-0.617725	0.140615
C	-3.143127	-0.032014	1.356461
C	-3.543911	-0.570941	-1.008527
C	-2.457332	-3.416828	0.337880
H	-3.331591	-2.905561	0.741378
H	-2.227874	-4.286570	0.955479
H	-2.716212	-3.775717	-0.663894
C	0.000011	1.848187	0.799297
H	-0.000008	2.002578	1.875540
C	-1.229136	2.350080	0.090545
H	-2.063301	2.790662	0.629525
C	-1.228274	2.131242	-1.243840
H	-2.056832	2.387622	-1.897627
C	2.457265	-3.416854	0.338017
H	2.716308	-3.775621	-0.663760
H	2.227716	-4.286669	0.955477
H	3.331457	-2.905631	0.741715

C	-2.280372	-0.122815	2.608875
H	-1.238785	-0.220679	2.289379
C	3.143056	-0.031997	1.356470
C	3.543912	-0.570990	-1.008487
C	-5.179634	0.695210	0.259404
H	-6.130614	1.216660	0.303092
C	1.228365	2.131208	-1.243796
H	2.056956	2.387556	-1.897553
C	1.229200	2.350035	0.090590
H	2.063357	2.790599	0.629599
C	-4.376958	0.616395	1.392077
H	-4.713716	1.079550	2.313650
C	0.000041	1.416470	-1.749811
H	0.000063	1.217334	-2.819400
C	-4.765127	0.102324	-0.924666
H	-5.402720	0.162213	-1.802059
C	2.280272	-0.122753	2.608866
H	1.238686	-0.220580	2.289353
C	-2.623828	-1.380386	3.421860
H	-3.675355	-1.365694	3.729148
H	-2.005396	-1.431310	4.324086
H	-2.452076	-2.294530	2.847191
C	4.765132	0.102263	-0.924608
H	5.402751	0.162120	-1.801985
C	2.359094	1.123907	3.496257
H	3.325060	1.203665	4.005971
H	1.589153	1.073039	4.272415
H	2.199836	2.042032	2.924373
C	-2.359145	1.123844	3.496266
H	-2.199782	2.041961	2.924395
H	-1.589241	1.072914	4.272459
H	-3.325126	1.203677	4.005940
C	4.376906	0.616378	1.392115
H	4.713649	1.079536	2.313693
C	-3.150518	-1.241679	-2.317541
H	-2.198778	-1.759089	-2.163109
C	5.179619	0.695158	0.259467
H	6.130608	1.216588	0.303180
C	3.150575	-1.241802	-2.317484
H	2.198878	-1.759285	-2.163032
C	-2.937888	-0.228066	-3.449603
H	-3.848797	0.349084	-3.640912
H	-2.669808	-0.746201	-4.376035
H	-2.137559	0.473919	-3.211389
C	2.937858	-0.228258	-3.449593
H	2.137462	0.473664	-3.211414
H	2.669822	-0.746460	-4.375999
H	3.848714	0.348965	-3.640930
C	2.623664	-1.380346	3.421847
H	2.451918	-2.294473	2.847148
H	2.005196	-1.431274	4.324047
H	3.675179	-1.365684	3.729177
C	4.193870	-2.290073	-2.736443
H	5.135780	-1.813782	-3.028196
H	3.833304	-2.862555	-3.597109

H	4.417790	-2.988991	-1.925314
C	-4.193714	-2.290034	-2.736542
H	-4.417547	-2.989016	-1.925445
H	-3.833098	-2.862436	-3.597243
H	-5.135676	-1.813827	-3.028261
Ca	0.000084	4.855080	-0.890227
H	-0.000115	5.335514	-2.979075
H	0.000280	5.721490	1.072932

	88		
C4			
Al	0.000003	-0.007444	-0.486945
N	1.426669	0.095414	0.786486
N	-1.426669	0.095412	0.786499
C	0.000005	0.030246	2.727254
H	0.000007	-0.053075	3.806471
C	1.253206	0.057940	2.114103
C	2.747221	0.316497	0.253881
C	-1.253205	0.057921	2.114107
C	-2.747222	0.316471	0.253888
C	3.615521	-0.763757	0.032890
C	3.109154	1.633852	-0.089796
C	2.445559	-0.008013	3.033374
H	3.336484	0.455133	2.608738
H	2.667071	-1.065103	3.220034
H	2.216980	0.458164	3.992894
C	0.000035	-1.328756	-2.131066
H	0.000093	-2.404653	-2.344339
C	1.229852	-0.678963	-2.733438
H	2.030988	-1.264936	-3.171533
C	1.226507	0.663175	-2.684092
H	2.048053	1.275338	-3.043029
C	-2.445548	-0.008049	3.033389
H	-2.217000	0.458223	3.992871
H	-2.666964	-1.065146	3.220130
H	-3.336509	0.454993	2.608719
C	3.267996	-2.183533	0.452371
H	2.316212	-2.153138	0.996203
C	-3.615486	-0.763798	0.032821
C	-3.109183	1.633832	-0.089728
C	5.216441	0.779600	-0.933973
H	6.177598	0.959336	-1.405282
C	-1.226570	0.663070	-2.684161
H	-2.048141	1.275163	-3.043158
C	-1.229798	-0.679066	-2.733510
H	-2.030852	-1.265111	-3.171659
C	4.847605	-0.508430	-0.572859
H	5.531970	-1.331108	-0.760348
C	-0.000074	1.263012	-2.055949
H	-0.000118	2.348418	-1.982626
C	4.354825	1.840977	-0.681221
H	4.657298	2.845993	-0.957201
C	-3.267957	-2.183578	0.452290
H	-2.316224	-2.153169	0.996208
C	4.312795	-2.769417	1.412929

H	5.268502	-2.941835	0.906649
H	3.966117	-3.729128	1.809064
H	4.500475	-2.098867	2.255877
C	-4.354832	1.840947	-0.681205
H	-4.657329	2.845967	-0.957140
C	-3.100469	-3.106303	-0.763712
H	-4.032416	-3.189938	-1.331486
H	-2.834565	-4.127915	-0.460310
H	-2.356841	-2.721499	-1.470831
C	3.100644	-3.106294	-0.763622
H	2.357062	-2.721535	-1.470815
H	2.834756	-4.127908	-0.460217
H	4.032645	-3.189903	-1.331312
C	-4.847547	-0.508482	-0.572980
H	-5.531883	-1.331170	-0.760529
C	2.190826	2.811680	0.207088
H	1.165629	2.433800	0.239133
C	-5.216400	0.779555	-0.934055
H	-6.177540	0.959285	-1.405400
C	-2.190911	2.811664	0.207316
H	-1.165712	2.433792	0.239431
C	2.232376	3.901268	-0.869173
H	3.173577	4.461104	-0.850209
H	1.425459	4.620994	-0.697640
H	2.106001	3.481220	-1.870959
C	-2.232364	3.901335	-0.868864
H	-2.105885	3.481375	-1.870676
H	-1.425471	4.621055	-0.697193
H	-3.173570	4.461166	-0.849944
C	-4.312825	-2.769519	1.412738
H	-4.500597	-2.098998	2.255688
H	-3.966156	-3.729231	1.808876
H	-5.268482	-2.941945	0.906366
C	-2.496076	3.408289	1.589512
H	-3.529703	3.769114	1.634906
H	-1.829575	4.252347	1.796389
H	-2.359585	2.671326	2.385467
C	2.495858	3.408418	1.589265
H	2.359293	2.671520	2.385267
H	1.829329	4.252488	1.796008
H	3.529478	3.769253	1.634729
Ca	0.000008	-3.579323	-0.229353
H	0.000056	-2.885742	1.792790
H	0.000014	-5.300803	-1.473494

	88		
C5			
Al	0.000074	-0.628473	-0.234620
N	1.419303	0.292017	0.816840
N	-1.419308	0.291877	0.816994
C	0.000070	0.567993	2.739726
H	0.000130	0.754901	3.806378
C	1.252108	0.547522	2.113243
C	-1.252052	0.547287	2.113405
C	2.733535	0.452358	0.265276

C	-2.733476	0.452639	0.265428
C	3.670923	-0.594268	0.338818
C	-3.671079	-0.593833	0.338526
C	3.039843	1.665530	-0.381989
C	-3.039489	1.666066	-0.381470
C	2.437513	0.824468	3.011424
C	-2.437420	0.823894	3.011727
H	2.793120	-0.124388	3.425786
H	-2.144081	1.462223	3.846565
H	2.144184	1.463205	3.845953
H	-3.270103	1.287516	2.482637
H	3.270205	1.287803	2.482105
H	-2.793071	-0.125150	3.425618
C	-1.274977	-1.082398	-1.980486
H	-2.361288	-1.068542	-1.944117
C	-0.680799	-2.106752	-2.879266
C	-0.676992	0.254107	-2.300975
H	-1.285530	-2.717494	-3.548054
H	-1.289352	1.115261	-2.539861
C	0.679870	-2.107237	-2.879355
C	0.677775	0.253598	-2.301073
H	1.284092	-2.718390	-3.548224
H	1.290725	1.114279	-2.540127
C	3.364088	-1.912308	1.038644
C	-3.364432	-1.912195	1.037801
H	2.365566	-1.833419	1.478495
H	-2.365848	-1.833632	1.477578
C	5.237636	0.779614	-0.903150
C	-5.237471	0.780826	-0.902985
H	6.212721	0.905673	-1.363322
H	-6.212524	0.907247	-1.363126
C	4.920343	-0.407039	-0.255534
C	-4.920434	-0.406138	-0.255786
H	5.657090	-1.204755	-0.214460
H	-5.657345	-1.203719	-0.215046
C	1.274833	-1.083316	-1.980532
H	2.361167	-1.070172	-1.944305
C	4.302234	1.806864	-0.960262
C	-4.301845	1.807879	-0.959733
H	4.560915	2.730125	-1.468430
H	-4.560296	2.731378	-1.467587
C	4.363446	-2.209008	2.167241
C	-4.363749	-2.209116	2.166391
H	5.363116	-2.409742	1.767127
H	-5.363505	-2.409404	1.766272
H	4.049130	-3.092860	2.731671
H	-4.049625	-3.093286	2.730431
H	4.452369	-1.370365	2.862508
H	-4.452374	-1.370738	2.862013
C	-3.343108	-3.094736	0.056090
C	3.342443	-3.095194	0.057357
H	-4.342398	-3.303740	-0.340477
H	4.341663	-3.304507	-0.339241
H	-2.994783	-4.007369	0.554794
H	2.993993	-4.007596	0.556383

H	-2.710017	-2.876312	-0.809673
H	2.709302	-2.876929	-0.808396
C	2.024342	2.800953	-0.425045
C	-2.023758	2.801288	-0.424125
H	1.033903	2.337681	-0.451628
H	-1.033399	2.337829	-0.450452
C	2.152376	3.693150	-1.664258
C	-2.151252	3.693691	-1.663239
H	3.044247	4.327939	-1.619154
H	-3.042938	4.328747	-1.618245
H	1.286075	4.359007	-1.728815
H	-1.284735	4.359297	-1.727505
H	2.200982	3.111860	-2.590052
H	-2.199840	3.112554	-2.589129
C	-2.084335	3.666281	0.844133
C	2.084669	3.666126	0.843085
H	-3.085840	4.091208	0.975957
H	3.086201	4.090901	0.975193
H	-1.369988	4.493440	0.770676
H	1.370503	4.493409	0.769230
H	-1.833716	3.094316	1.740008
H	1.833606	3.094381	1.738975
Ca	-0.000547	-3.600672	-0.702855
H	-0.000080	-2.005267	0.816889
H	-0.000600	-5.699657	-0.443808

	88		
C6*			
Al	-0.033007	0.441247	-0.278403
N	1.432382	-0.519834	-1.020884
N	-1.393117	-0.679067	-1.052073
C	0.072299	-1.584390	-2.713678
H	0.107492	-2.154648	-3.633196
C	1.304010	-1.233447	-2.151120
C	-1.204069	-1.406487	-2.142137
C	2.741211	-0.416494	-0.428936
C	-2.677908	-0.590084	-0.415540
C	3.613146	0.616015	-0.813319
C	-3.633649	0.333828	-0.870282
C	3.111830	-1.368481	0.543570
C	-2.897571	-1.376655	0.732529
C	2.545483	-1.714896	-2.861306
C	-2.367173	-2.072009	-2.832102
H	3.110577	-0.861777	-3.245077
H	-3.178188	-2.298014	-2.138176
H	2.287143	-2.367991	-3.694843
H	-2.762636	-1.393582	-3.595623
H	3.210300	-2.251495	-2.180318
H	-2.047809	-2.988872	-3.330040
C	-1.212812	2.605685	3.002973
H	-1.990828	3.348665	3.183331
C	-1.288057	1.764571	1.884232
C	-0.185856	2.441335	3.936597
H	-2.194420	1.740287	1.280396
H	-0.110166	3.101596	4.793985

C	-0.181651	0.837414	1.612438
C	0.523149	1.205009	3.914001
H	0.959221	1.588885	1.018922
H	1.093478	0.911556	4.792451
C	3.180113	1.743405	-1.739264
C	-3.391611	1.243400	-2.065749
H	2.157384	1.538246	-2.068228
H	-2.412580	0.999783	-2.488494
C	5.303639	-0.329437	0.643824
C	-5.086886	-0.358619	0.945461
H	6.308255	-0.304353	1.054210
H	-6.029041	-0.268276	1.476963
C	4.898905	0.634313	-0.267887
C	-4.840067	0.427602	-0.170730
H	5.589947	1.421953	-0.554870
H	-5.594967	1.134890	-0.502406
C	0.453376	0.367603	2.834830
H	0.959234	-0.592417	2.854794
C	4.408464	-1.310488	1.055734
C	-4.118442	-1.250535	1.392710
H	4.725736	-2.033993	1.798751
H	-4.312412	-1.844078	2.279756
C	4.058471	1.875582	-2.990500
C	-4.447942	1.040948	-3.161958
H	5.094581	2.111256	-2.725856
H	-5.436594	1.370545	-2.825264
H	3.686759	2.684140	-3.627873
H	-4.190008	1.624569	-4.051511
H	4.069757	0.957328	-3.584152
H	-4.534131	-0.010208	-3.452239
C	-3.344933	2.716654	-1.637000
C	3.166170	3.073286	-0.966758
H	-4.309812	3.045426	-1.236487
H	4.181525	3.446094	-0.795306
H	-3.092941	3.358687	-2.486748
H	2.616156	3.848363	-1.511731
H	-2.589071	2.867899	-0.861553
H	2.720582	2.920485	0.023801
C	2.126099	-2.422906	1.037430
C	-1.836107	-2.346807	1.226271
H	1.139453	-1.948244	1.052522
H	-0.868660	-1.943866	0.916451
C	2.420585	-2.899118	2.464763
C	-1.793025	-2.473969	2.750817
H	3.295904	-3.557295	2.499356
H	-2.666647	-3.004996	3.144948
H	1.569274	-3.474436	2.841459
H	-0.907168	-3.046295	3.045582
H	2.595479	-2.063956	3.148976
H	-1.731085	-1.490382	3.224537
C	-1.987205	-3.721525	0.559831
C	2.028851	-3.640638	0.105212
H	-2.963040	-4.161095	0.794945
H	3.017133	-4.087820	-0.050937
H	-1.209945	-4.405375	0.917466

H	1.380816	-4.401531	0.552877
H	-1.898313	-3.654720	-0.528619
H	1.604235	-3.385941	-0.867831
Ca	0.148129	3.630928	0.695769
H	-0.280556	1.909284	-0.997202
H	0.257220	5.645290	0.062918

73

LAl^{I}

Al	-0.000002	-0.224876	-0.978596
N	-1.389161	-0.137133	0.451449
N	1.389159	-0.137144	0.451450
C	-0.000002	-0.204400	2.408410
H	-0.000002	-0.231891	3.490488
C	-1.249421	-0.160683	1.777638
C	-2.714730	-0.010850	-0.092265
C	1.249418	-0.160697	1.777638
C	2.714731	-0.010874	-0.092261
C	-3.217721	1.274513	-0.367307
C	-3.453079	-1.166898	-0.404298
C	-2.474853	-0.124989	2.657682
H	-3.053317	0.784734	2.472344
H	-2.201545	-0.161352	3.712738
H	-3.134642	-0.967624	2.432232
C	2.474850	-0.125011	2.657684
H	3.134667	-0.967614	2.432193
H	2.201543	-0.161431	3.712739
H	3.053284	0.784740	2.472390
C	-2.422910	2.534595	-0.053604
H	-1.526942	2.239478	0.500519
C	3.217753	1.274488	-0.367260
C	3.453053	-1.166928	-0.404337
C	-5.218530	0.249672	-1.277963
H	-6.193459	0.350735	-1.744691
C	-4.476113	1.380254	-0.961984
H	-4.879448	2.363272	-1.188299
C	-4.706280	-1.010951	-0.999188
H	-5.289216	-1.891161	-1.255028
C	2.422968	2.534575	-0.053514
H	1.527023	2.239462	0.500649
C	-3.208479	3.507596	0.835908
H	-4.089481	3.903729	0.319823
H	-2.578143	4.358626	1.113695
H	-3.551330	3.023639	1.755935
C	4.706256	-1.010990	-0.999225
H	5.289170	-1.891206	-1.255095
C	1.957148	3.223888	-1.343879
H	2.813062	3.558577	-1.940435
H	1.343165	4.100359	-1.110441
H	1.363595	2.542016	-1.961101
C	-1.957145	3.223900	-1.343993
H	-1.363629	2.542020	-1.961240
H	-1.343140	4.100364	-1.110586
H	-2.813082	3.558600	-1.940510
C	4.476143	1.380218	-0.961938

H	4.879502	2.363235	-1.188222
C	-2.914066	-2.563830	-0.133003
H	-1.995620	-2.461886	0.452765
C	5.218533	0.249630	-1.277958
H	6.193463	0.350685	-1.744686
C	2.914011	-2.563857	-0.133082
H	1.995547	-2.461910	0.452658
C	-2.545971	-3.271173	-1.445399
H	-3.432880	-3.414192	-2.072845
H	-2.112176	-4.256149	-1.241825
H	-1.819872	-2.685235	-2.017505
C	2.545952	-3.271175	-1.445501
H	1.819883	-2.685218	-2.017625
H	2.112132	-4.256147	-1.241957
H	3.432880	-3.414203	-2.072917
C	3.208582	3.507568	0.835965
H	3.551489	3.023601	1.755967
H	2.578258	4.358592	1.113797
H	4.089553	3.903713	0.319835
C	3.890284	-3.414400	0.690959
H	4.806265	-3.632509	0.131728
H	3.428462	-4.372118	0.951902
H	4.179988	-2.911801	1.619314
C	-3.890376	-3.414349	0.691019
H	-4.180120	-2.911723	1.619347
H	-3.428567	-4.372061	0.952007
H	-4.806332	-3.632473	0.131755

85

Uncatalyzed1

Al	-0.186579	-0.193472	0.607696
N	0.982332	-1.108968	-0.746328
N	-1.759533	-0.699002	-0.532770
C	-0.695404	-2.185005	-2.087005
H	-0.863651	-2.882480	-2.897762
C	0.635202	-1.954590	-1.718788
C	2.371992	-0.773524	-0.606554
C	-1.822555	-1.527912	-1.573279
C	-2.964182	-0.040659	-0.108387
C	2.911699	0.221557	-1.447166
C	3.150905	-1.384806	0.390511
C	1.706989	-2.671062	-2.505345
H	2.244907	-1.966436	-3.147805
H	1.273459	-3.450020	-3.133893
H	2.447541	-3.118070	-1.837212
C	1.582044	3.451212	1.443615
H	1.406968	4.106511	0.594908
C	2.663568	2.572443	1.433937
H	3.330415	2.532125	0.578630
C	2.880398	1.723269	2.516233
H	3.713531	1.027784	2.494948
C	-3.148564	-1.791383	-2.245529
H	-3.838935	-2.282247	-1.553307
H	-3.023368	-2.425297	-3.124029
H	-3.621431	-0.852538	-2.546820

C	2.065414	0.904419	-2.513845
H	1.187117	0.281226	-2.702819
C	-3.217065	1.271147	-0.549157
C	-3.831741	-0.687044	0.793227
C	5.021703	0.013775	-0.269715
H	6.053144	0.324387	-0.134255
C	0.931187	2.627033	3.615883
H	0.248026	2.637889	4.459531
C	0.714851	3.477834	2.533711
H	-0.135503	4.153320	2.535459
C	4.240653	0.599539	-1.260124
H	4.673787	1.369538	-1.891711
C	2.014694	1.751469	3.607441
H	2.176219	1.080013	4.445645
C	4.477221	-0.971645	0.541433
H	5.093725	-1.430295	1.309842
C	-2.268869	2.013578	-1.480700
H	-1.422926	1.354672	-1.699925
C	2.800058	1.063007	-3.850276
H	3.623755	1.781406	-3.782472
H	2.109014	1.432552	-4.614621
H	3.214409	0.110551	-4.196226
C	-4.971826	-0.005336	1.219862
H	-5.650727	-0.484589	1.919149
C	-1.708365	3.272009	-0.803514
H	-2.506597	3.981073	-0.558542
H	-1.001009	3.780528	-1.466928
H	-1.185462	3.014133	0.123191
C	1.548242	2.256647	-2.007404
H	0.992612	2.141804	-1.071474
H	0.885827	2.717424	-2.748670
H	2.379726	2.945124	-1.816354
C	-4.375531	1.909697	-0.100720
H	-4.591639	2.920331	-0.436005
C	2.601171	-2.481115	1.290129
H	1.570156	-2.679919	0.982196
C	-5.251355	1.280396	0.772986
H	-6.145131	1.793794	1.113827
C	-3.532121	-2.075105	1.340932
H	-2.720618	-2.505616	0.746950
C	2.565335	-2.035128	2.757897
H	3.574406	-1.826854	3.132451
H	2.130509	-2.821391	3.384173
H	1.962544	-1.129696	2.872463
C	-3.035569	-1.981432	2.791603
H	-2.152281	-1.338986	2.864003
H	-2.773903	-2.974351	3.173194
H	-3.812567	-1.560927	3.439991
C	-2.936177	2.354152	-2.819930
H	-3.314389	1.455446	-3.317380
H	-2.217460	2.836756	-3.490777
H	-3.778874	3.040627	-2.683208
C	-4.731855	-3.025921	1.236109
H	-5.550078	-2.720028	1.896544
H	-4.435860	-4.038007	1.530180

H	-5.127411	-3.069107	0.216186
C	3.387866	-3.789854	1.130286
H	3.412850	-4.119305	0.086671
H	2.927393	-4.584126	1.726816
H	4.423631	-3.677773	1.468746

85
Uncatalyzed2*

Al	-0.000002	0.451331	0.322012
N	-1.415233	0.708664	-0.895148
N	1.415218	0.708661	-0.895164
C	-0.000015	1.626057	-2.612466
H	-0.000018	2.126888	-3.571582
C	-1.259261	1.293303	-2.081960
C	-2.711202	0.267854	-0.450356
C	1.259237	1.293297	-2.081974
C	2.711188	0.267849	-0.450374
C	-3.109932	-1.049452	-0.735216
C	-3.494606	1.126186	0.340329
C	-2.484809	1.623047	-2.891731
H	-3.087101	0.727961	-3.066777
H	-2.215199	2.065517	-3.850521
H	-3.117995	2.325233	-2.339769
C	0.000016	-0.821016	1.784071
H	0.000002	-1.329354	0.538753
C	-1.224746	-1.225855	2.454049
H	-2.186363	-0.893578	2.071048
C	-1.196507	-2.052920	3.560659
H	-2.146677	-2.336316	4.009543
C	2.484775	1.623043	-2.891760
H	3.117891	2.325342	-2.339859
H	2.215148	2.065396	-3.850599
H	3.087143	0.727989	-3.066699
C	-2.231816	-2.018289	-1.512782
H	-1.280278	-1.520239	-1.721795
C	3.109906	-1.049465	-0.735218
C	3.494602	1.126185	0.340296
C	-5.148139	-0.638201	0.514000
H	-6.101781	-0.993235	0.891730
C	1.196577	-2.052918	3.560634
H	2.146757	-2.336311	4.009499
C	1.224792	-1.225856	2.454022
H	2.186403	-0.893589	2.070994
C	-4.347276	-1.476420	-0.250084
H	-4.680295	-2.488958	-0.457771
C	0.000041	-2.507654	4.130297
H	0.000051	-3.147878	5.005188
C	-4.717389	0.648361	0.812969
H	-5.336737	1.286898	1.435432
C	2.231783	-2.018305	-1.512771
H	1.280236	-1.520265	-1.721761
C	-2.863065	-2.402011	-2.858301
H	-3.817885	-2.919248	-2.714012
H	-2.198464	-3.074351	-3.410582
H	-3.050215	-1.523740	-3.484300

C	4.717386	0.648357	0.812933
H	5.336743	1.286898	1.435383
C	1.915913	-3.266618	-0.676872
H	2.817728	-3.859815	-0.489893
H	1.204693	-3.904728	-1.210757
H	1.481129	-2.998716	0.290886
C	-1.915916	-3.266592	-0.676882
H	-1.481150	-2.998682	0.290880
H	-1.204674	-3.904681	-1.210762
H	-2.817717	-3.859814	-0.489913
C	4.347252	-1.476434	-0.250092
H	4.680262	-2.488976	-0.457769
C	-3.009838	2.509026	0.745997
H	-2.137703	2.756631	0.131957
C	5.148126	-0.638210	0.513975
H	6.101770	-0.993247	0.891701
C	3.009851	2.509032	0.745959
H	2.137699	2.756634	0.131942
C	-2.554047	2.495724	2.214117
H	-3.398967	2.284852	2.878632
H	-2.133565	3.467239	2.494264
H	-1.793457	1.726915	2.393304
C	2.554109	2.495761	2.214095
H	1.793540	1.726943	2.393332
H	2.133620	3.467276	2.494231
H	3.399056	2.284922	2.878586
C	2.863010	-2.402009	-2.858306
H	3.050141	-1.523729	-3.484296
H	2.198403	-3.074348	-3.410583
H	3.817836	-2.919241	-2.714040
C	4.058795	3.600298	0.500001
H	4.933467	3.474740	1.146431
H	3.633012	4.585713	0.713785
H	4.407127	3.595629	-0.537988
C	-4.058781	3.600301	0.500085
H	-4.407130	3.595658	-0.537899
H	-3.632990	4.585709	0.713885
H	-4.933443	3.474732	1.146527

85
Uncatalyzed3

Al	-0.039172	-0.799693	0.696919
N	-1.386633	-0.800367	-0.688345
N	1.424314	-0.949127	-0.539086
C	0.034889	-2.062087	-2.157876
H	0.041550	-2.701933	-3.030935
C	-1.214055	-1.517379	-1.791623
C	-2.657426	-0.197647	-0.402761
C	1.283456	-1.719934	-1.622858
C	2.707637	-0.377028	-0.251772
C	-3.668549	-0.957117	0.211171
C	-2.819948	1.177530	-0.661621
C	-2.383157	-1.783591	-2.706266
H	-3.022147	-2.556862	-2.267947
H	-2.044047	-2.128442	-3.683450

H	-3.001156	-0.891736	-2.831664
C	-0.087757	0.876521	1.744953
H	-0.190250	-2.175185	1.500420
C	-1.294321	1.271168	2.355196
H	-2.185076	0.653268	2.253273
C	-1.406445	2.452866	3.086237
H	-2.358943	2.724188	3.533194
C	2.517000	-2.246460	-2.312671
H	3.193384	-1.437759	-2.597573
H	2.256733	-2.822246	-3.201005
H	3.071701	-2.889690	-1.621162
C	-3.464911	-2.409335	0.618610
H	-2.520680	-2.759153	0.191681
C	3.549311	-0.979417	0.698686
C	3.067914	0.823687	-0.894981
C	-5.077145	1.017065	0.214136
H	-6.025016	1.491634	0.448728
C	0.914993	2.916472	2.664419
H	1.789132	3.550972	2.783571
C	1.013042	1.730876	1.935325
H	1.979370	1.476438	1.506507
C	-4.878706	-0.324213	0.507390
H	-5.673703	-0.889704	0.984725
C	-0.299209	3.284913	3.238394
H	-0.380827	4.208567	3.804191
C	-4.050221	1.759784	-0.357916
H	-4.206120	2.815710	-0.549179
C	3.148105	-2.248905	1.432504
H	2.245587	-2.644941	0.957728
C	-4.583015	-3.326481	0.104172
H	-5.538419	-3.109834	0.593817
H	-4.337578	-4.372483	0.313323
H	-4.734409	-3.219108	-0.974591
C	4.304060	1.392159	-0.585739
H	4.605818	2.313838	-1.075115
C	2.795879	-1.936393	2.894228
H	3.673785	-1.565392	3.435409
H	2.433779	-2.836469	3.400919
H	2.013675	-1.174070	2.964051
C	-3.334443	-2.521392	2.145035
H	-2.502145	-1.917291	2.516155
H	-3.146892	-3.560090	2.436047
H	-4.252397	-2.186505	2.641188
C	4.767788	-0.361871	0.989942
H	5.427274	-0.805466	1.730164
C	-1.680346	2.009221	-1.234082
H	-0.749105	1.578660	-0.853759
C	5.150365	0.809563	0.350245
H	6.103765	1.272611	0.585145
C	2.146920	1.518443	-1.889042
H	1.192852	0.983992	-1.894750
C	-1.713696	3.465275	-0.761296
H	-2.559145	4.018015	-1.185775
H	-0.802579	3.978647	-1.078689
H	-1.768707	3.520045	0.330091

C	1.858503	2.965261	-1.469699
H	1.409956	3.007128	-0.473281
H	1.165502	3.428694	-2.179329
H	2.770246	3.571978	-1.459694
C	4.225161	-3.337904	1.345863
H	4.499482	-3.548053	0.306977
H	3.858353	-4.265289	1.797003
H	5.137252	-3.050393	1.879760
C	2.710006	1.475833	-3.316723
H	3.669479	2.001457	-3.378206
H	2.015575	1.959791	-4.011546
H	2.866390	0.449812	-3.661639
C	-1.634633	1.928158	-2.766307
H	-1.475520	0.902962	-3.112960
H	-0.813237	2.539492	-3.155543
H	-2.569354	2.296821	-3.203837

3. References

[S1] M. Stender, R. J. Wright, B. E. Eichler, J. Prust, M. M. Olmstead, H. W. Roesky and P. P. Power, *J. Chem. Soc., Dalton Trans.*, 2001, 3465–3469.

[S4] C. Cui, H. W. Roesky. H.-G. Schmidt, M. Noltemeyer, H. Hao and F. Cimpoesu, *Angew. Chem. Int. Ed.*, 2000, **39**, 4274–4276.

[S3] A. S. S. Wilson, M. S. Hill, M. F. Mahon, C. Dinoi, L. Maron, *Science*, 2017, **358**, 1168-1171.

[S4] S. P. Green, C. Jones, A. Stasch, *Angew. Chem. Int. Ed.*, 2008, **47**, 9079–90.

[S5] J. Spielmann, D. Piesik, B. Wittkamp, G. Jansen, S. Harder, *Chem.Commun.*, 2009, 3455–3456

[S6] T. N. Hooper, M. Garçon, A. J. P. White, M. R. Crimmin, *Chem. Sci.*, 2018, **9**, 5435-5440

[S6] T. N. Hooper, M. Garçon, A. J. P. White, M. R. Crimmin, *Chem. Sci.*, 2018, **9**, 5435-5440

[S7] Rigaku Oxford Diffraction, **2018**, CrysAlisPro Software system, version 1.171.39.46, Rigaku Corporation, Oxford, UK.

[S8] O. V. Dolomanov, L. J. Bourhis, R.J. Gildea, J. A. K. Howard and H. Puschmann, *J. Appl. Cryst.,* 2009, **42**, 339–341.

[S9] G. M. Sheldrick, *Acta Cryst. A,* 2015, **71**, 3–8.

[S10] G. M. Sheldrick, *Acta Cryst. C,* 2015, **71**, 3–8.

[S11] A. Thorn, B. Dittrich and G. M. Sheldrick, *Acta Cryst. A,* 2012, **68**, 448–451.

[S12] M. J. Frisch, G. W. Trucks, H. B. Schlegel, G. E. Scuseria, M. A. Robb, J. R. Cheeseman, G. Scalmani, V. Barone, B. Mennucci, G. A. Petersson, H. Nakatsuji, M. Caricato, X. Li, P. H. Hratchian, A. F. Izmaylof, J. Bloino, G. Zheng, J. L. Sonnenberg, M. Hada, M. Ehara, K. Toyota, R. Fukuda, J. Hasegawa, M. Ishida, T. Makajima, Y. Honda, O. Kitao, H. Nakai, T. Vreven, J. A. Montgomery, J. E. Peralta, F. Ogilaro, M. Bearpark, J. J. Heyd, E. Brothers, K. N. Kudin, V. N. Staroverov, T. Keith, R. Kobayashi, J. Normand, K. Raghavachari, A. Rendell, J. C. Burant, S. S. Iyengar, J. Tomasi, M. Cossi, N. Rega, J. M. Millam, M. Klene, J. E. Knox, J. E. Cross, V. Bakken, C. Adamo, J. Jaramillo, R. Gomperts, R. E. Stratmann, O. Yazyev, A. J. Austin, R. Cammi, C. Pomelli, J. W. Ochterski, R. L. Martin, K. Morokuma, V. G. Zakrezewski, G. A. Voth, P. Salvador, J. J. Dannenberg, S. Dapprich, A. D. Daniels, O. Farkas, J. B. Foresman, J. V. Ortiz, J. Cioslowski, D. J. Fox, Gaussian, Gaussian, Inc., Wallingford CT, 2013.

[S13] J. Chai, M. Head-gordon, Phys. Chem. Chem. Phys. 2008, 6615–6620.

[S14] W. J. Hehre, L. Radom, P. v. R. Schleyer, J. A. Pople, Ab Initio Molecular Orbital Theory by W. J. Hehre, L. Radom, P. v. R. Schleyer, and J. A. Pople, John Wiley, New York, 548pp. Price: $79.95 (1986), John Wiley, New York, 1986.

[S15] T. Clark, J. Chandrasekhar, G. W. Spitznagel, P. v. R. Schleyer, J. Comp. Chem. 1983, 4, 294–301.

[S16] E. F. Silva, H. F. Svendsen, K. M. Merz, J. Phys. Chem. A 2009, 6404–6409.

[S17] A. E. Reed, R. B. Weinstock, F. Weinhold, J. Chem. Phys. 1985, 83, 735–746.

[S18] N. van Eikema Hommes, Molecule, Erlangen, 2016.

[S19] T. A. Keith, AIMAll (Version 17.01.25), TK Gristmill Software, Overland Park KS USA, 2017.

[S20] R. F. W. Bader, Chem. Rev. 1991, 91, 893–928.
